# *In-Silico* Analysis of Binding Site Features and Substrate Selectivity in Plant Flavonoid-3-O Glycosyltransferases (F3GT) through Molecular Modeling, Docking and Dynamics Simulation Studies

**DOI:** 10.1371/journal.pone.0092636

**Published:** 2014-03-25

**Authors:** Ranu Sharma, Priyabrata Panigrahi, C.G. Suresh

**Affiliations:** Division of Biochemical Sciences, CSIR-National Chemical Laboratory, Pune, Maharashtra, India; National Institute for Medical Research, Medical Research Council, London, United Kingdom

## Abstract

Flavonoids are a class of plant secondary metabolites that act as storage molecules, chemical messengers, as well as participate in homeostasis and defense processes. They possess pharmaceutical properties important for cancer treatment such as antioxidant and anti-tumor activities. The drug-related properties of flavonoids can be improved by glycosylation. The enzymes glycosyltransferases (GTs) glycosylate acceptor molecules in a regiospecific manner with the help of nucleotide sugar donor molecules. Several plant GTs have been characterized and their amino acid sequences determined. However, three-dimensional structures of only a few are reported. Here, phylogenetic analysis using amino acid sequences have identified a group of GTs with the same regiospecific activity. The structures of these closely related GTs were modeled using homologous GT structures. Their substrate binding sites were elaborated by docking flavonoid acceptor and UDP-sugar donor molecules in the modeled structures. Eight regions near the acceptor binding site in the N- and C- terminal domain of GTs have been identified that bind and specifically glycosylate the 3-OH group of acceptor flavonoids. Similarly, a conserved motif in the C-terminal domain is known to bind a sugar donor substrate. In certain GTs, the substitution of a specific glutamine by histidine in this domain changes the preference of sugar from glucose to galactose as a result of changed pattern of interactions. The molecular modeling, docking, and molecular dynamics simulation studies have revealed the chemical and topological features of the binding site and thus provided insights into the basis of acceptor and donor recognition by GTs.

## Introduction

**Figure 1:**
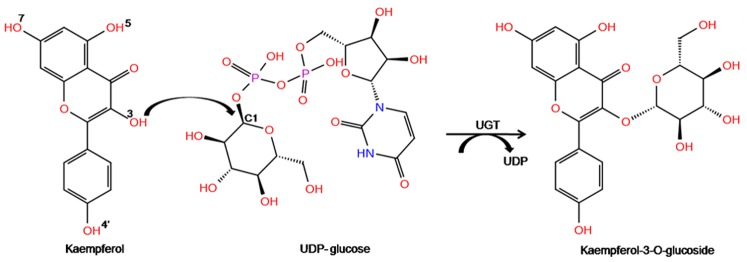
Reaction mechanism of UGT. Reaction catalyzed by UGT is shown with Kaempferol and UDP-glucose as substrates that react to form Kaempferol-3-O-glucoside along with the release of UDP moiety. The numbering of the OH groups- 7-OH, 5-OH, 3-OH and 4′-OH in flavonoids is shown.

Glycosyltransferases (GTs; EC 2.4.x.y) belong to the transferase family of enzymes which catalyze the transfer of sugar moiety from an activated nucleotide sugar donor molecule to a saccharide or nonsaccharide acceptor substrate to synthesize oligosaccharides, polysaccharides or glycoconjugates [1] (Figure 1). Sugar donors are activated nucleotide molecules possessing a variant sugar moiety, and an invariant UDP group. The acceptor molecules are generally flavonoids consisting of flavanones, flavanonol, flavans, flavones, flavonols, and anthocyanidins. Flavonoids, a class of plant secondary metabolites, play a prominent role as anti-cancer and antioxidant agents [2]. UDP-glycosyltransferases (UGTs) are GTs which transfer the glycosyl group (glucose, galactose, xylose, rhamnose, etc) from a nucleoside diphosphate sugar donor (UDP-sugar) to a diverse array of acceptor substrates [3]. In addition to the specific sugars they transfer, some UGTs are also highly specific to acceptor molecules and they catalyze glycosylation of only one or two types of molecules [4], whereas some others catalyze glycosylation of a broad range of acceptors [5]. Flavonoids possess a polyphenolic structure bearing multiple -OH groups (7-OH, 5-OH, 3-OH, 4′-OH etc) on the phenyl rings (Figure 1). Some UGTs specifically glycosylate only one of these -OH groups, whereas others glycosylate multiple -OH groups.

**Figure 2:**
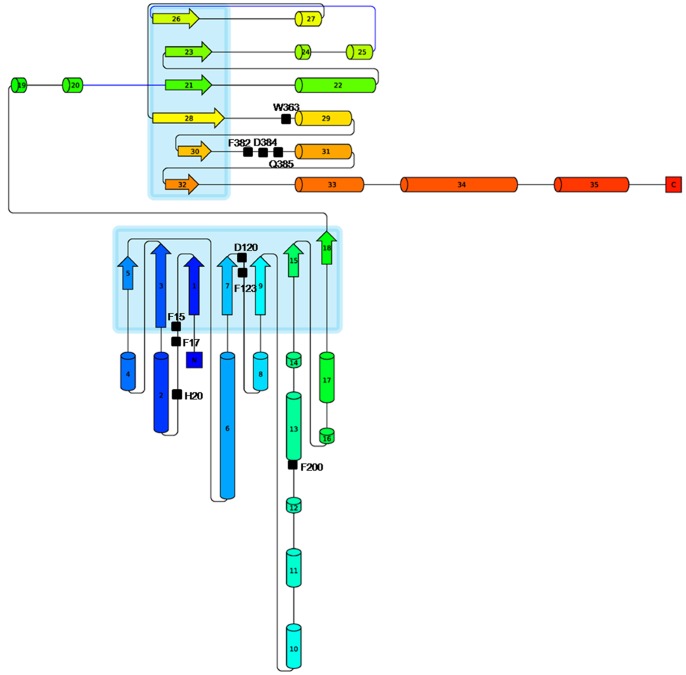
Topology diagram of UGT. Topology diagram of UGT from *F. ananassa* shows CTD and NTD having an architecture of Rossmann fold. An arrangement of alternate β/α/β secondary structure elements can be seen in the diagram along with the key catalytic and binding site residues of acceptor and sugar donor shown by square mark and labeled.

On the basis of amino acid sequence similarity, GTs have been classified into 94 CAZy families [6]. The amino acid sequence is poorly conserved across GTs [7], [8]. However, despite lack of sequence conservation, available three-dimensional crystal structures of plant UGTs show highly conserved secondary and tertiary structures [8], [9], [10]. Only two types of structural folds of GTs have been identified, namely GT-A and GT-B. Both have a Rossmann-like fold architecture, a characteristic feature of nucleotide binding proteins. The Rossmann fold has an arrangement of alternate β/α/β secondary structure elements which form a sandwich structure with nucleotide binding site between the layers [5] (Figure 2). The GT-A fold has a single Rossmann domain that requires a divalent cation such as Mg^2+^ or Mn^2+^ for its catalytic activity [11]. The GT-B fold has two Rossmann domains connected by a linker region, and the substrate binding pocket is in a narrow cleft between the domains. The C-terminal domain (CTD) of GT-B proteins has a long stretch of 44 amino acid residues called Plant Secondary Product Glycosyltransferase [12] or Putative Plant Secondary Glycosyltransferase (PSPG) motif [3] (Figure S1). This motif plays a crucial role in sugar recognition by facilitating the binding of a sugar donor molecule in the active site. For example, in the *Vitis vinifera* UGT crystal structure (PDB-2C1Z), out of the ten residues interacting with UDP-sugar, seven interact with the invariant part of the donor. The remaining three interact with the variant part of the donor. The conserved Trp residue is the 22^nd^ amino acid of the PSPG motif, while the last two residues, 43^rd^ and 44^th^ amino acids of PSPG, are Asp/Glu/Ser and Gln/Glu/His/Asn. These last two residues are aspartic acid (D) or glutamic acid (E) in the first place and glutamine (Q) at the second place in four available plant GT structures. The residues in the above three sites W, D/E, and Q are positioned to form hydrogen bonds with the sugar part of the donor [13]. The remaining amino acids of PSPG box, which are not involved in the protein-ligand interactions, help in maintaining the 3-D structure of the protein.

Contrary to the existence of a conserved CTD binding site, there are pronounced variations in the loops and helix regions of the N-terminal domain (NTD) near the acceptor binding site, presumably to accommodate a wide range of acceptor molecules. Even the reaction mechanism of these enzymes is conserved. The transfer of sugar to an acceptor substrate occurs by an oxycarbenium ion formation. It catalyzes the transfer of glucose moiety from a sugar donor to an acceptor molecule with a direct displacement S_N_^2^ mechanism. The conserved histidine residue, close to the acceptor and sugar donor, acts as a catalytic base that facilitates the deprotonation of the acceptor molecule (Figure S1, Region N1). The deprotonated acceptor, which turns into a nucleophilic oxyanion, attacks the C1 carbon of the sugar and forms the respective β-glucosides. A neighboring aspartic acid residue interacts with the conserved histidine by forming a hydrogen bond and helps to balance the charge on the histidine subsequent to deprotonation of the acceptor substrate [3] (Figure S1, Region N4).

A phylogenetic, sequence and structural analysis of GT-B family of enzymes from various plant sources has been carried out to identify motifs and crucial amino acids which play an important role in the binding and recognition of specific acceptors, also to identify amino acid residues involved in maintaining the 3-D structure.

## Materials and Methods

### Sequence Comparison and Phylogenetic Analysis

Protein sequences of plant GTs of sequence length 430–480 amino acids were retrieved from UniProtKB/Swiss-Prot database (http://www.uniprot.org/) using query search. A dataset of 101 protein sequences of plant GTs was obtained. Protein sequences were initially aligned using **MU**ltiple **S**equence **C**omparison by **L**og- **E**xpectation (MUSCLE) [14] employing the Unweighted Pair Group Method with Arithmetic Mean (UPGMA) based clustering method with gap open and gap extension penalties set to −2.9 and 0. This alignment was used to build the phylogenetic tree using the Neighbor Joining (NJ) algorithm of Molecular Evolutionary Genetics Analysis 5.1 (MEGA) [15] with the Dayhoff substitution matrix (PAM250) and bootstrap value set to 1000. A well defined cluster for flavonoid-3-O-GT (F3GT) was observed in the dendrogram comprising thirty protein sequences. Multiple sequence alignment (MSA) of these thirty F3GT protein sequences was done to analyze the extent of conservation among the sequences using the ClustalX program [16]. A percent identity matrix of F3GTs was prepared with the help of ClustalX program.

### Molecular Modeling of F3GT Proteins

A search using Basic Local Alignment Search Tool (BLAST) algorithm [17] was carried out against the Protein Data Bank (PDB) [18] to select the high resolution crystal structures of homologous proteins. The sequence identity cut off was set to ≥30% (E-value cut off = 1). Homology modeling of F3GT protein sequences was carried out using the Composite/Chimeric model type of Prime 3.1 [19], by taking structures homologous to the target proteins as templates, in order to study their structural features, binding mode and affinity with the substrates.

### Model Validation and Refinement

The initial models were evaluated for the stereochemical quality of the protein backbone and side chains using PROCHECK [20]. Environment of the atoms in the protein model was checked by ERRAT server [21]. Verify3D plot [22] showed the compatibility of the 3-D structure with respect to the protein sequence. Errors in the model structures were also checked with ProSA server [23]. After model validation, initial models were refined using impref minimization of protein preparation wizard [19] and Impact 5.8 [19] minimization. These energy minimized final models were further used for the binding studies with their substrates.

### Ligand Design, Protein and Ligand Preparation

The 2-D sketcher utility of Maestro 9.3 was used to design the structures of ligand molecules in two-dimensions, which were then converted into three-dimensional structures in mol2 format. Before proceeding with the docking studies, water and other hetero atom groups from the protein structures were removed using protein preparation utility of Maestro. Hydrogens were added subsequently to carry out restrained minimization of the models. The minimization was done using impref utility of Maestro in which the heavy atoms were restrained such that the strains generated upon protonation could be relieved. The root mean square deviation (RMSD) of the atomic displacement for terminating the minimization was set as 0.3 Å. Similarly, ligands were refined with the help of LigPrep 2.5 [24] to define their charged state and enumerate their stereo isomers. The processed receptors and ligands were further used for the docking studies using Glide 5.8 [25].

### Modeling Enzyme-ligand Interactions

Reported wet lab experiments have shown that some of the F3GT enzymes have high binding affinity and specificity for 3-OH group of flavonoids, more specifically flavonols and anthocyanidins, which include kaempferol (kmp), myricetin, quercetin, delphinidin and many more. Therefore, kmp was docked in the acceptor binding site cavity of the prepared receptor molecules. A grid was made either by taking the reference ligand or by selecting the active site residues involved in the binding of the substrate. Flexible ligand docking was carried out using the standard precision option. The sugar donor was docked in the active site by creating a grid around the bound reference ligand i.e. U2F (Uridine-5′-Diphosphate-2-deoxy-2-fluoro-alpha-D-glucose). Final products of the reaction were also docked in the *V. vinifera* GT crystal structure. A total of 20 poses generated were scored on the basis of their glide score and E-model values. The hydrogen bond interactions between the protein and ligands were visualized using PyMOL [26].

### Molecular Dynamics Simulations to Assess the Stability of Docked Complexes

The docked complexes were subjected to molecular dynamics simulations using the GROningen Machine for Chemical Simulations V4.5.4 (GROMACS) [27], [28]. GROMOS96 43a1 force field was applied on 31 docked complexes placed in the centre of the dodecahedron box solvated in water. Topology files and other force field parameter files for the ligands were created using PRODRG2 server [29]. Protein was immersed in dodecahedron water box of SPC216 water model with distance between the solute and the box was set to 10 Å. The initial simulation cell dimensions set up were approximately 90×90×90 Å for all the simulations (Table S1). The system was initially energy minimized by steepest descent minimization for 50,000 steps until a tolerance of 10 kJ/mol to avoid the high energy interactions and steric clashes. Total negative charges on the docked structures were balanced by suitable number of Na+ ions to make the whole system neutral using genion program of GROMACS. After adding ions, the system was again energy minimized by steepest descent minimization retaining the same parameters. The V-rescale, a modified Berendsen thermostat, temperature coupling [30] and Parrinello-Rahman pressure coupling [31] methods were used to keep the system stable at 300 K temperature and pressure of 1 bar. The Particle Mesh Ewald (PME) method [32] was selected to compute long range electrostatic interactions. A cut off distance of 9 Å and 14 Å was set for Coulombic and van der Waals interactions. Rotational constraint was applied to bonds by LINCS algorithm [33].

The analysis was performed on the ensemble of system configurations extracted at 2 ps time intervals from the simulation and was equilibrated for 2 ns time period, to guarantee a completely equilibrated system. The analysis was carried out relative to the last 3 ns time period on the C-alpha atoms of the structures. No positional constraints were applied on the system. Periodic boundary conditions were applied in all three directions. The trajectories were visualized using Visual Molecular Dynamics program (VMD) [34].

## Results and Discussion

### Amino Acid-based Phylogenetic Analysis of GTs

After creating a dataset of 101 protein sequences of plant GTs, a dendrogram was built using neighbor joining method of MEGA in order to study their evolutionary relation (Table S2). Bootstrapping (value: 1000) was performed to examine how often a particular cluster in a tree appeared on re-sampling amino acids.

**Figure 3:**
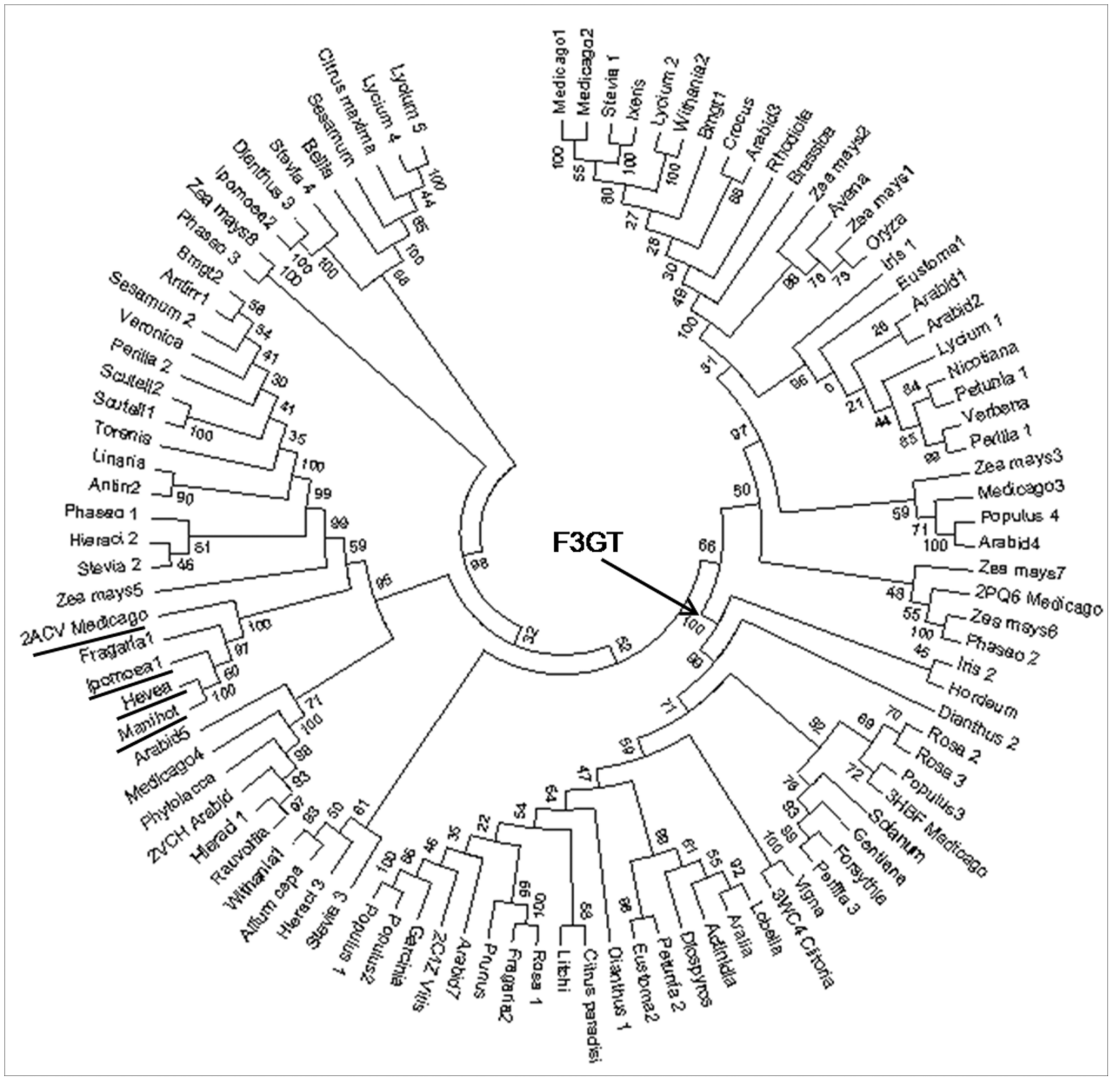
Phylogenetic tree of 101 UGTs. Dendrogram of 101 UGT protein sequences shows distinct clusters of F3GT members marked with an arrow. The underlined taxon names are annotated to be flavonoid-3-OH glycosyltransferases and they form a separate clade.

The GT sequences clustered into few distinct groups in the output of phylogenetic analysis. Out of them, the maximum number of GTs clustered in the F3GT specific clade with a significantly high bootstrap value (Figure 3). The members of the remaining clusters showed mixed specificity/activity for different -OH groups of acceptors. Here, the focus of study is the F3GT specific cluster, as the members of this group are closely related to each other showing high sequence identity and present in significant numbers. The percentage identity matrix revealed a significant level of conservation with approximate 40% sequence identity among them (Table S3).

### Molecular Models of F3GT Proteins

The molecular structures of F3GT proteins were modeled in order to study, from a structural point of view, the role of conserved regions in the close vicinity of acceptor and their interactions that favor formation of protein-ligand complex. BLAST search against PDB identified homologous structures to be used as templates to build the homology models (Table S4). Crystal structures of GTs from *V. vinifera* (PDB-2C1Z), *Medicago truncatula* (PDB-3HBF) and *Clitoria ternatea* (PDB-3WC4) of resolution 1.90 Å, 2.10 Å and 1.85 Å, respectively, shared an identity of ≥40% (except *Iris_2* and *Hordeum*) and query coverage of ∼>90% with the target proteins, and were thus used for modeling studies. Secondary structure prediction by Psipred showed the similarity between the templates and the target with respect to the arrangement of secondary structure elements. Very few gaps were present in the alignment of the target and template sequences (Figure S2). The bound ligands of 2C1Z i.e., kmp and U2F were incorporated in the target models during the modeling procedure. Few N-terminal residues (4–5 amino acids) couldn’t be modeled for some of the target sequences because of the absence of corresponding regions in the templates (Missing residues in 2C1Z: 1–5, 3HBF: 1–11).

### Model Validation and Refinement

The initial models were evaluated for their stereochemical parameters. More than 99% of all residues were in the allowed regions of Ramachandran plot. The goodness factor of the models, a log odd score calculated based on the stereochemical parameters, were in the range of 0 to −0.4. Verify3D plot for the models showed that more than 95% of the residues had a positive 3D-1D averaged score, which revealed that the models were folded correctly. RMSD of the C-alpha atoms of the models and the templates were close to 1 Å. The z-score value calculated by ProSA server is within the z-scores of experimentally determined PDB structures. The ERRAT results also showed that the overall quality score of the models was good (Table 1).

**Table 1:** Model evaluation statistics of 30 F3GT protein sequences.

No	Protein source	Ramachandran plot	ProSA	ERRAT	Verify3D
1	Populas_1	100*, 0.0** (0.0)***	−10.7	85.05	98.20
2	Populas_2	100, 0.0 (−0.4)	−11.9	83.22	100.00
3	Aralia	100, 0.0 (0.0)	−10.75	90.13	99.33
4	Fragaria2	99.5, 0.5 (−0.2)	−11.17	87.67	92.37
5	2C1Z_Vitis				
6	Actinidia	100,0.0 (0.0)	−11.19	84.06	100
7	Eustoma	99.5, 0.5 (0.0)	−11.47	88.86	97.33
8	Lobelia	99.7, 0.3 (−0.1)	−10.51	79.8	97.10
9	Rosa_1	98.8, 0.2 (0.0)	−11.37	90.11	91.09
10	Petunia_2	99.5, 0.5 (0.0)	−11.29	81.73	98.66
11	Litchi	99.7, 0.3 (0.0)	−11.68	81.79	97.98
12	Arabid7	99.5, 0.5 (0.0)	−10.88	99.63	97.78
13	Citrus_paradisi	99.5, 0.5 (−0.1)	−11.77	83.18	99.55
14	Rosa_2	99.5, 0.5 (−0.1)	−11.13	81.7	99.77
15	Garcinia	99.5, 0.5 (−0.2)	−10.71	89.70	96.44
16	Prunus	98, 2.0 (−0.1)	−10.72	83.90	99.33
17	Diospyros	99.7, 0.3 (0.6)	−10.79	81.75	95.31
18	Perilla_3	99.5, 0.5 (−0.1)	−11.26	81.79	97.97
19	Forsythia	98.5, 1.5 (0.0)	−12.05	84.2	100
20	Solanum	98.2, 1.8 (−0.1)	−11.13	77.93	97.48
21	Gentiana	98.7, 1.3 (0.0)	−10.85	83.52	98.43
22	3HBF_Medicago				
23	Vigna	99.7, 0.3 (−0.2)	−10	70.27	99.11
24	Populas3	99.7, 0.3 (−0.3)	−10.62	81.10	99.10
25	Rosa_3	99.2, 0.8 (0.1)	−10.73	71.66	100.00
26	Dianthus_1	99.7, 0.3 (0.0)	−12.06	84.93	99.11
27	Dianthus_2	99.0, 1.0 (−0.1)	−10.74	88.24	98.66
28	Iris_2	98.4, 1.6 (−0.1)	−11.43	72.74	94.13
29	3WC4_Clitoria				
30	Hordeum	98.7, 1.3 (−0.1)	−10.66	87.96	100.00

*Represent percent of the total residue present in allowed region of Ramachandran plot.

**Represent percent of the total residue present in disallowed region of Ramachandran plot.

***Number in brackets denotes the value of goodness factor for the models. The values in the fourth, fifth and sixth column represent the Z-score, ERRAT and Verify3D scores of F3GT models.

The model refinement phase involved preprocessing the initial models by adding hydrogens, assigning bond order, and filling missing loops and side chains. Next, the models were subjected to restrained minimization by applying the constraint to converge the non-hydrogen atoms to an RMSD of 0.3 Å using OPLS 2005 [35] force field. After that, the models were further subjected to 500 steps of steepest descent energy minimization followed by 1000 steps of conjugate gradient energy minimization using the same force field. These energy minimized models were further used for docking and molecular dynamics studies.

### Sequence Conservation and Structural Integrity of F3GT Enzymes

MSA of the F3GT proteins was carried out using ClustalX program (Figure S1). Sequence alignment and construction of percent identity matrix showed higher sequence conservation of both CTD and NTD (Figure S1 and Table S3). The residue numbers used in this analysis are with respect to the sequence of transferase from *V. vinifera* source (PDB-2C1Z). An analysis of the conserved residues using the *V. vinifera* GT structure revealed that these residues interacted through hydrophobic, π-π stacking and hydrogen bonding interactions for maintaining the required framework of interacting residues in the binding site.

**Figure 4:**
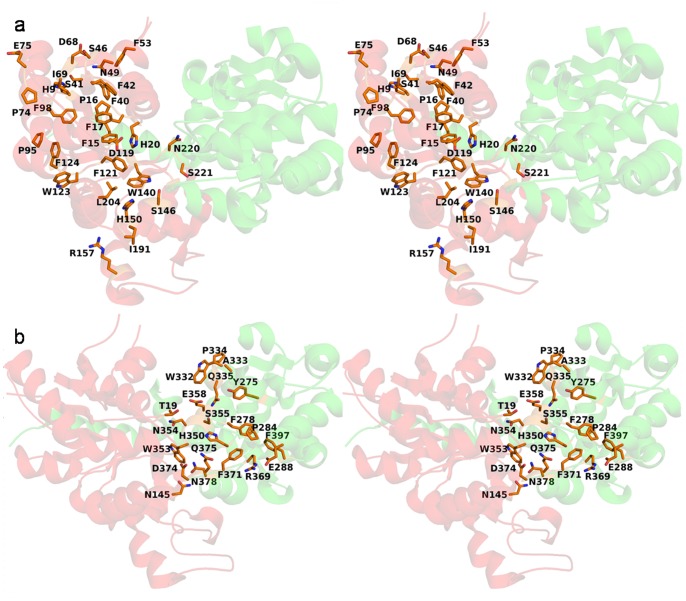
Conserved amino acids of N-terminal and C-terminal domain of UGT. a Stereo image representing cartoon drawing of UGT from *V. vinifera* with conserved amino acids of the NTD shown in stick form. **b** Stereo image representing cartoon drawing of UGT from *V. vinifera* with conserved amino acids of the CTD shown in stick form.

One key conserved residue involved in catalysis is His20, which steers the deprotonation of acceptor substrates. An important interaction near the binding site is the side chain-side chain hydrogen bonding interaction between His9 and Ser41, which in turn helps Phe42 to make a π-π interaction with Phe53. Similarly, Phe40 is surrounded by hydrophobic amino acids of the adjacent α-helix and β-sheets that provide stability to the folded structure. Pro16 present near the acceptor binding site provides rigidity to the binding site loop and helps in orienting the neighboring residues Phe15 and Phe17 to provide a hydrophobic environment to the acceptor. Similarly, the conserved Gly72 residue provides flexibility to a binding site loop. Another important interaction involves residue Pro74 that orients the side chain of Glu75, making it accessible to the solvent. Phe90 is surrounded by a series of hydrophobic and aromatic amino acids such as Phe15, Pro16, Phe17, Ile69, Phe98, etc. Side chain of Asn49 interacts with Ser46 and Asp68 of neighboring β-sheet. The interaction of Asp119 with the key catalytic residue His20 balances its acquired charge after deprotonation of the acceptor. Phe121 provides hydrophobic environment to one of the phenyl rings of the acceptor molecule. Conserved Trp123 and Phe124 are involved in π-π interaction while Pro95 forms a stacking interaction with Phe124. The indole ring of Trp140 interacts with Gly143 and Ser146, its aromatic ring providing a hydrophobic environment to the acceptor substrate. His150 being a polar amino acid provides a hydrophilic environment to the 4′-OH group of the flavonoid acceptor. Arg157 connects the other loop by making hydrogen bonds with residues Gly190 and Ile191 and also interacting with the solvent molecule. Leu204 also contributes to the hydrophobicity of the acceptor binding pocket. Conserved Asn220 and Ser221 stabilize a gamma turn that folds the polypeptide chain, bringing together and allowing interactions between regular secondary structure elements (Figure 4a).

The CTD shows a higher level of sequence conservation and binds the nucleotide sugar donor substrate. Side chain of Tyr275 interacts with the conserved Gln335 residue and helps in maintaining the structure by connecting the β-sheet with the α-helix. In addition, conserved Phe278 is involved in hydrophobic interactions with Pro284, Phe371 and Phe397. The residues Glu288 and Arg369 are involved in ionic interactions that stabilize the structure by connecting the α-helix to a coil. Pro334 present at the beginning of the α-helix helps the neighboring Gln335 to interact with Tyr275 of a proximate β-sheet. Trp332 stacks with the uridine ring of the UDP-sugar substrate. Residues Thr19, Ala333, His350, Trp353, Asn354, Ser355, Glu358, Asp374 and Gln375 also interact with the sugar donor. Asn378 interacts with Asp374 and Asn145 thus helping in maintaining the 3-D structure by connecting two secondary structural elements (Figure 4b). Conserved amino acid residues with less bulky side chains provide required flexibility to the loops surrounding the binding site. These features are conserved or semi-conserved in most of the proteins of the F3GT cluster.

### Interaction Studies using Modeled Complexes

#### Interactions between enzyme and substrates

Prior to the docking, the models and the ligands were first pre-processed to check for any problems in the structures related to missing hydrogens, side chains, improper bond orders, overlapping atoms etc. The refined sugar donor substrate was docked in the binding site cavity of the processed target by using standard precision mode of Glide. The bound ligand of template was used to mark the binding site by creating a grid around it. A total of 20 docked complexes were generated out of which the one with the highest glide score and forming all possible interactions with the conserved residues of PSPG motif was selected as the best pose (Table 2).

**Table 2:** Docking statistics (Glide score values) of sugar donor and acceptor substrates for 30 F3GT proteins.

Sr. No	Protein source	Glide score	
		Sugar donor	Acceptor
1	Populas_1	−11.56	−9.07
2	Populas_2	−11.06	−8.84
3	Aralia	−9.52	−8.94
4	Fragaria2	−11.08	−9.28
5	2C1Z_Vitis	Crystal structure with bound ligands	
6	Actinidia	−10.98	−8.02
7	Eustoma2	−10.37	−8.84
8	Lobelia	−9.81	−7.63
9	Rosa_1	−10.31	−7.83
10	Petunia_2	−9.50	−9.52
11	Litchi	−10.79	−8.83
12	Arabid7	−11.40	−7.34
13	Citrus_paradisi	−11.52	−9.02
14	Rosa_2	−11.66	−7.58
15	Garcinia	−10.64	−8.40
16	Prunus	−10.96	−9.42
17	Diospyros	−9.54	−8.71
18	Perilla_3	−10.30	−8.45
19	Forsythia	−10.92	−7.02
20	Solanum	−11.25	−7.41
21	Gentiana	−10.63	−8.34
22	3HBF Medicago	−8.40	−7.34
23	Vigna	−9.78	−7.73
24	Populas3	−10.82	−8.98
25	Rosa_3	−9.32	−9.29
26	Dianthus_1	−10.51	−8.75
27	Dianthus_2	−10.84	−8.25
28	Iris_2	−9.82	−8.28
29	3WC4_Clitoria	−10.76	−7.63
30	Hordeum	−11.67	−7.89

**Figure 5:**
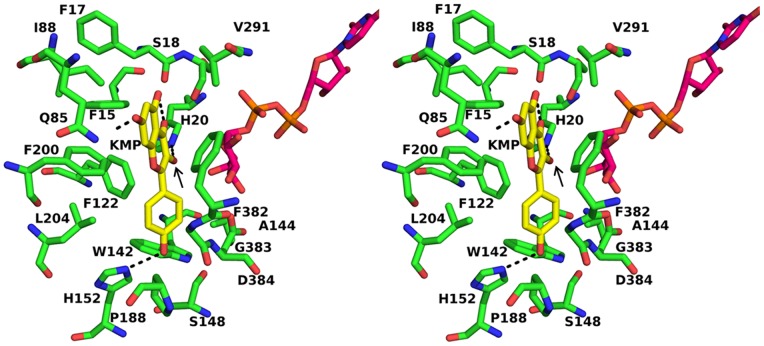
Docked complex of UGT with kaempferol and UDP-glucose. Stereo view of the docked complex of UGT from *F. ananassa* with kaempferol shown in stick form. The arrow shows the 3-OH group of kaempferol which take part in glycosylation event. UDP-glucose is also shown in stick form.

**Figure 6:**
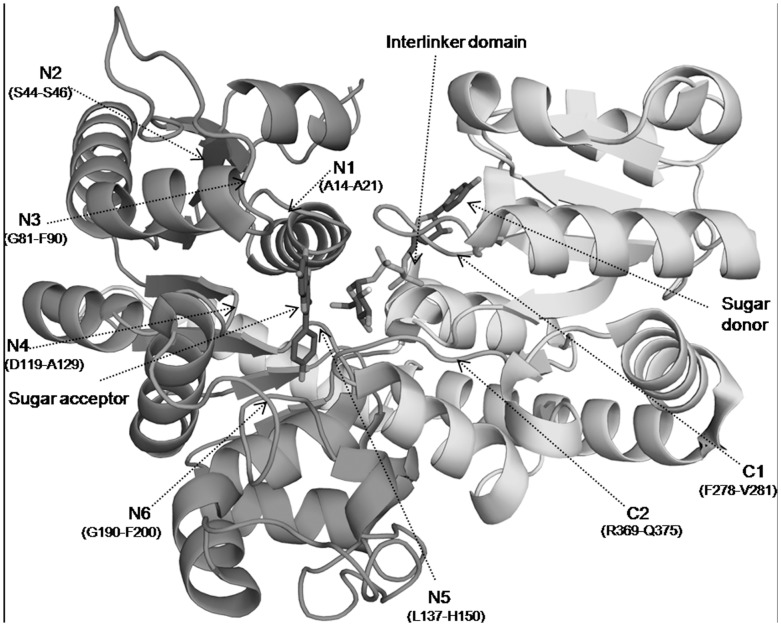
Conserved regions near the acceptor binding region. Ribbon view of UGT from *V. vinifera* with docked acceptor and sugar donor in stick form is shown. Six conserved regions from N1 to N6 at the NTD and two regions C1 and C2 at the CTD marked with an arrow play a crucial role in holding the acceptor in the binding pocket.

After the preparation of acceptor through LigPrep, kmp was docked in the acceptor binding pocket of the protein-UDP sugar complex by placing the grid around the reference ligand i.e. kmp. Standard precision mode of glide was used for docking studies. Similarly, 20 docked poses were generated and the one with a better glide score value and that interacted with the catalytic histidine was chosen as the best pose (Table 2). Docking studies have shown that the environment surrounding the acceptor is conserved or similar for all the docked structures. Some changes have been seen near the 5-OH and 4′-OH groups of kmp. In some structures 5-OH group is stabilized by the hydrophilicity provided by the side chain of Ser18 (w.r.t. PDB-2C1Z) while in some structures non-polar amino acids like glycine and alanine are present in that position. In the crystal structure 3HBF, glycine occupies this position but neighboring water molecule stabilizes the ligand molecule by forming a hydrogen bond with its 5-OH group. Amino acid Gln188 interacts with the O4′ group of kmp. In the F3GT proteins this glutamine is replaced by proline (close to N6; Figure S1), so this interaction is lost in all the docked complexes. This is eventually compensated by another interaction formed by His150 (Figure S1, Region N5) (Figure 5). The glide score value was higher for the pose in which 3-OH group of kmp interacted with the catalytic histidine residue while the other poses in which 7-OH, 4′OH and 5-OH groups of kmp interacted with the histidine had a low glide score. Docking studies aided in the identification of eight conserved regions present in the loops and helix near the acceptor binding regions (N1–N6 regions in the NTD and C1–C2 regions in the CTD) which might decide their substrate/glycosylation preference (Figure 6, Figure S1). This observation suggests that the environment topology observed here favors the binding of flavonoids to facilitate the glycosylation of 3-OH group of flavonoids.

Four protein sequences of *Fragaria ananassa* (Swiss-Prot ID-Q2V6K0), *Manihot esculenta*, *Hevea brasiliensis* and *M. truncatula* (Swiss-Prot ID-Q5IFH7) were annotated as flavonoid-3-O-glycosyltransferases in the protein sequence database but they were present outside the F3GT cluster in the dendrogram (Figure 3). Modeling and docking studies of these proteins showed that their activity might be less towards the flavonoid-3-OH group as compared to the other annotated members of F3GT. This is an additional evidence to show that the environment provided by the conserved acceptor binding site residues of F3GTs is more favorable for orienting the acceptor molecule for glycosylation at 3-OH group of the flavonoid molecule than sequences outside the F3GT cluster.

#### Docking studies with reaction products: Kaempferol-3-O-glucoside and UDP

**Figure 7:**
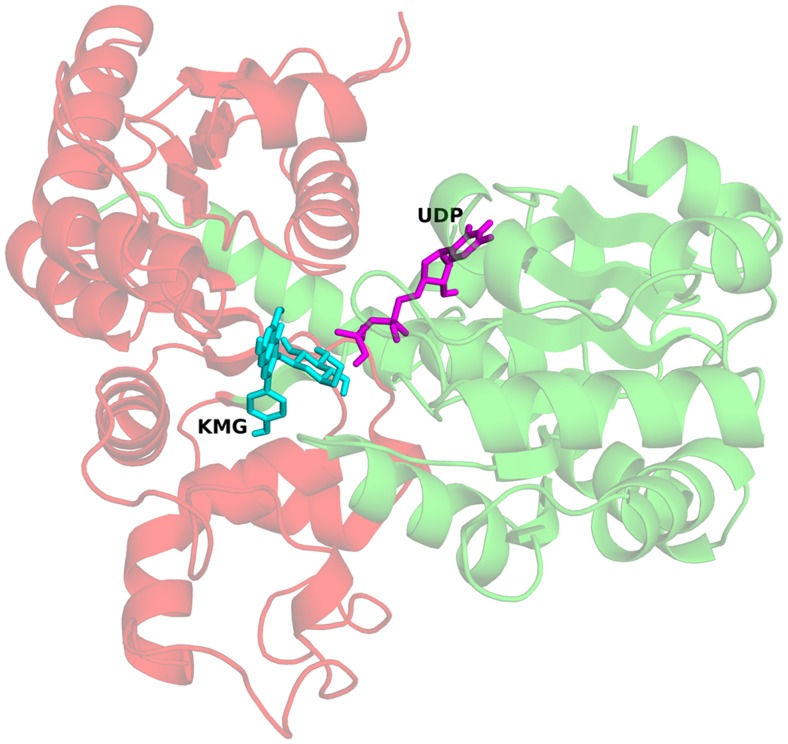
Docked complex of UGT with kaempferol-3-O-glucoside and UDP. Ribbon view of UGT from *F. ananassa* with docked Kaempferol-3-O-glucoside and UDP shown in stick form.

**Figure 8:**
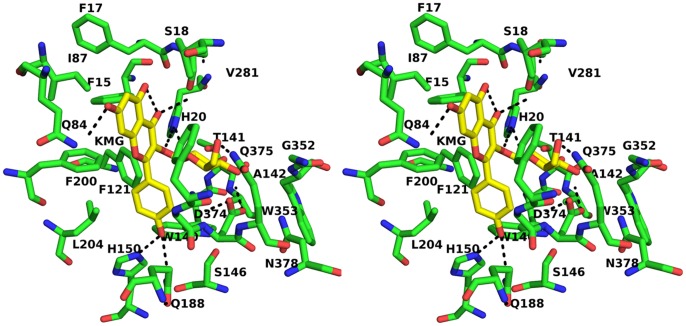
Kaempferol-3-O-glucoside interactions with the UGT. Stereo view of docked complex of UGT from *F. ananassa* with Kaempferol-3-O-glucoside shown in stick form.

The crystal structure from *V. vinifera* (PDB-ID: 2C1Z) was taken as the target molecule to dock the glycosylated flavonoid, kaempferol-3-O-glucoside (KMG) and UDP in their respective binding pockets (Figure 7). Grid was created around the reference ligands, kmp (for KMG) and U2F (for UDP). The best pose for UDP and KMG had glide scores of −9.43 and −6.20 (Figure 8). The sugar moiety of KMG interacts with the sugar specificity determining amino acids of the PSPG motif.

### Molecular Dynamics Simulations of the Docked Complexes

**Figure 9:**
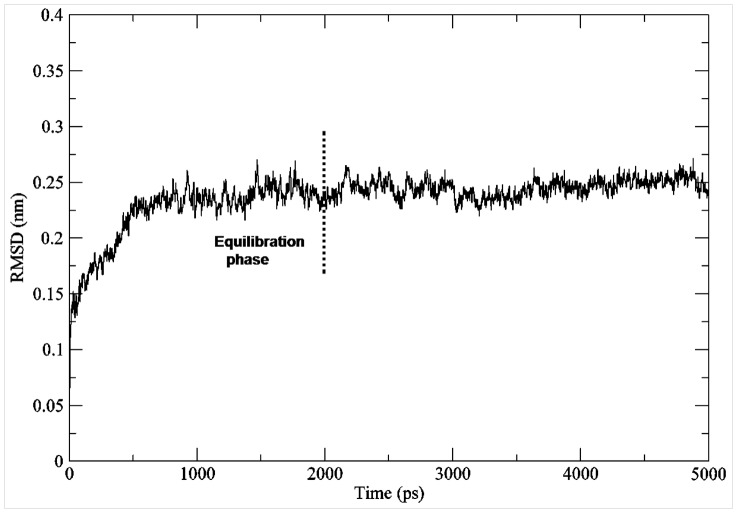
RMSD plot of *F. ananassa* UGT generated from MDS trajectory. RMSD plot of Cα atoms of generated structures of *F. ananassa* UGT with time shown in ps along X-axis and RMSD values in nm along Y-axis.

**Figure 10:**
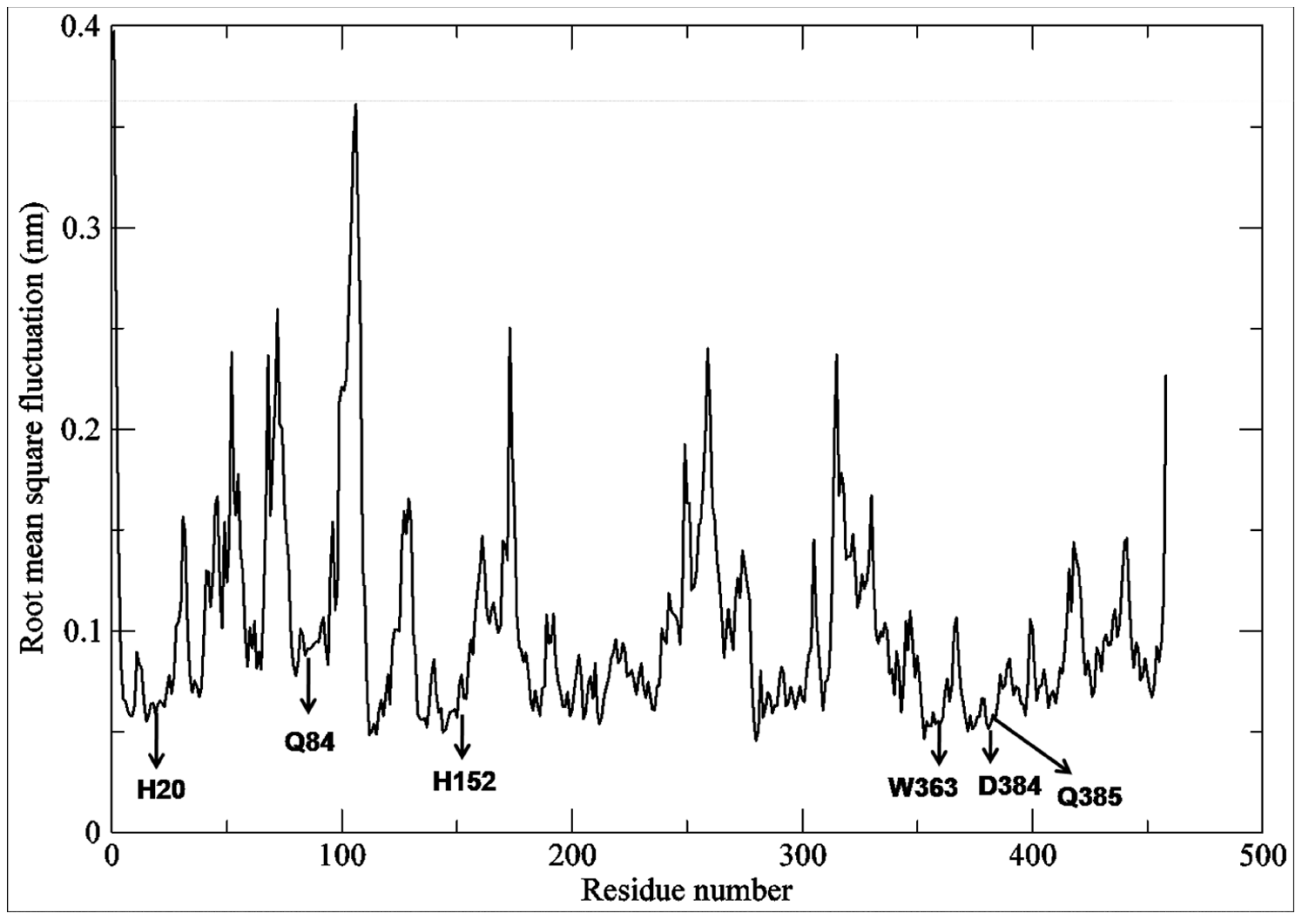
Mindist plot of kaempferol wrt the binding residues. Minimum distance plot between kaempferol and acceptor binding residues of *F. ananassa* UGT.

**Figure 11:**
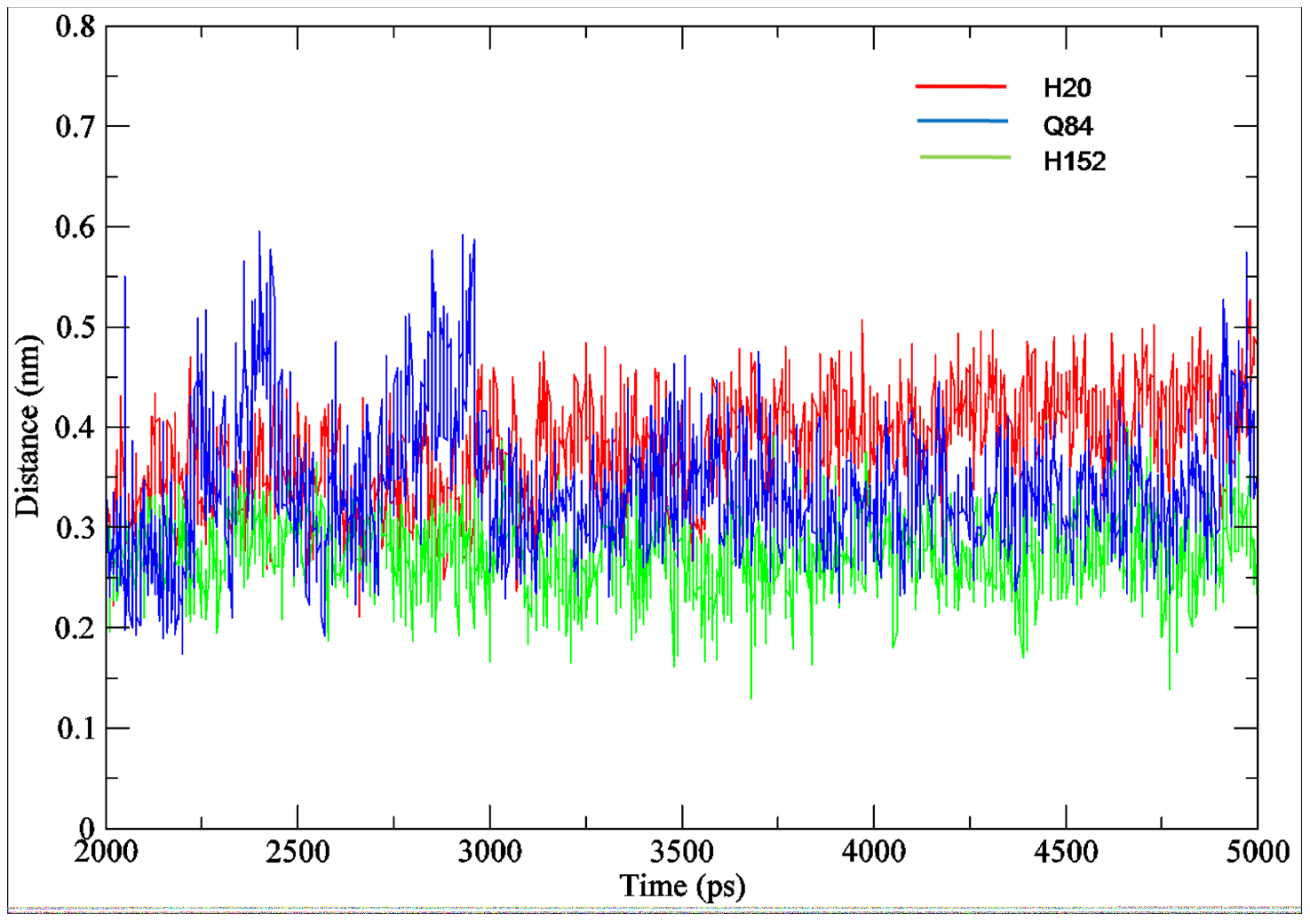
RMSF plot of *F. ananassa* UGT generated from MDS trajectory. RMSF plot *F. ananassa* UGT shows negligible fluctuation of catalytic and binding site residues of acceptor and sugar donor substrate.

Molecular dynamics simulations were performed on 31 docked complexes in order to check their stability. Their trajectories were visually inspected for the binding of ligands and it was observed that the substrates were firmly bound in their respective binding pockets (Video S1). Here we have explained the interactions and the stability patterns of the ligands for one of the F3GT members i.e. *F. ananassa* GT (Swiss-Prot ID-Q5UL10). The RMSD graph was drawn by assigning time in ps on the X-axis and RMSD values of the Cα atoms in nm on the Y-axis (Figure 9). From the graph, we can conclude that the RMSD of Cα atoms of the generated structures over the complete trajectory is stable. To check the fluctuations of kmp in binding site, the distance between kmp and three key residues H20, Q84 and H152 which form hydrogen bonds with 3-OH, 5-OH and 4′OH group of kmp was calculated using g_mindist program of GROMACS. The graph showed that kmp fluctuates in the binding pocket within a distance of only 3 Å to 5 Å with respect to three key residue considered in the analysis for the last 2 ns of the trajectory (Figure 10). The life of hydrogen bond between the catalytic H20 and D120 remained for 70% of the total simulation time after equilibration. The root mean square fluctuation (RMSF) plot showed negligible fluctuations of the catalytic and binding site residues of the acceptor and donor (Figure 11). A highly fluctuating loop region, not part of the binding site region, was observed in the plot. Molecular dynamics simulation showed that the final products also form a stable complex (Video S2).

### Experimental Evidence for F3GTs

Experimental evidence for the flavonoid 3-OH activity is known for UGTs enzymes of *Dianthus_1, Diospyros, Petunia_2, Gentiana, Iris_2, Vigna, Actinidia*, and *Perilla_3* included in the analysis [36]–[43]. Out of them, UGTs of *Dianthus_1, Diospyros and Petunia_2* were considered for analyzing their specificity against various flavonoid substrates. Few positive controls (3-OH group present: Kmp, Myricetin, Quercetin, and Fisetin) and negative control (3-OH group absent/present in different orientation: Apigenin, Naringenin, Dihyrdroquercetin (DHQ), Catechin, and Epicatechin) were selected to dock in the acceptor binding pocket of the protein. It has been observed that wherever the favorable binding pose was attained the flavonoid had its 3-OH present in close proximity of the catalytic histidine and sugar donor which is known to facilitate the nucleation during glycosylation reaction. However, the neighboring regions of the protein in the vicinity of catalytic base were not conducive for binding any other hydroxyl group of flavonoid (Figure S3, S4 and S5).

In order to further explore the importance of these crucial binding regions, flavonoid 7-O glucosyltransferase (F7GT) of *Scutellaria baicalensis* (Swiss-Prot ID: Q9SXF2), for which the flavonoid specificity was known, was selected for the study [44]. The three-dimensional structure of F7GT was modeled (Template: 2VCE; Resolution 1.9 Å, Identity: 30%) and three flavonoids namely baicalein, scutellarein, and kmp were chosen for the docking studies. The hydroxyl group is present on the C3 carbon atom of kmp but absent in other two. In spite of this difference the docked complexes showed that all the three ligands attained the favorable pose having only 7-OH closer to the catalytic histidine and sugar donor (Figure S6). Considering the flavonoid kmp, irrespective of it’s having both 7-OH and 3-OH, as per our classification F3GT favorably binds for 3-OH glycosylation and F7GT binds favoring 7-OH glycosylation. These results show how the acceptor binding site of UGTs decides the favorable binding mode of flavonoids and the nature of the glycosylation product formed.

## Conclusions

Homology modeling and docking studies of the F3GTs showed that the environment in the close vicinity of the acceptor is conserved in all the members of this group and that these amino acids favor the binding of a flavonoid such that the glycosylation of the 3-OH group is preferred over other hydroxyl groups. Eight regions in the acceptor binding site were identified as important for acceptor specificity. Alterations in these regions may affect the type of molecule to be glycosylated and change the acceptor specificity from one molecule to another or from one side group for glycosylation to another side group. These observations have been further confirmed using molecular dynamics simulations of the docked complexes. The binding site information was successfully exploited to predict UGTs with experimentally known 3-OH specificity as positive control and 7-OH specific UGTs along with different ligands as negative control. This knowledge can be implemented to design engineered GTs which can glycosylate flavonoids in a regiospecific manner. In this way, the biochemical and pharmaceutical properties of flavonoids can be altered to prepare more effective drug molecules in a desired chemical form.

## Supporting Information

Figure S1 **Multiple sequence alignment of 30 F3GT protein sequences.**
(PDF)

Figure S2 **Multiple sequence alignment between query (30F3GTs) and template (PDB ID: 2C1Z, 3HBF and 3WC4).**
(PDF)

Figure S3 **Image showing docked complexes of three positive and two negative control ligands in the acceptor binding pocket of** ***Dianthus caryophyllus*** **(** ***Dianthus_1*** **).**
(TIF)

Figure S4 **Image showing docked complexes of three positive and three negative control ligands in the acceptor binding pocket of** ***Diospyros kaki*** **.**
(TIF)

Figure S5 **Image showing docked complexes of three positive and three negative control ligands in the acceptor binding pocket of** ***Petunia hybrida*** **(** ***Petunia_2*** **).**
(TIF)

Figure S6 **Image showing docked complexes of three positive control ligands in the acceptor binding pocket of** ***Scutellaria baicalensis*** **.**
(TIF)

Table S1 Details of box size and number of water molecules added during simulation process.
(XLS)

Table S2 Percent identity matrix of 30 F3GT protein sequences.
(XLS)

Table S3 Dataset of 101 GT protein sequences taken for the phylogenetic study.
(XLS)

Table S4 Statistics of Blast results.
(XLS)

Video S1 **Multimedia file showing UGT docked with kaempferol and UDP-glucose.**
(MPG)

Video S2 **Multimedia file showing UGT docked with kaempferol-3-O-glucoside and UDP.**
(MPG)
