# Supplementary material for: In-Silico Analysis of Binding Site Features and Substrate Selectivity in Plant Flavonoid-3-O Glycosyltransferases (F3GT) through Molecular Modeling, Docking and Dynamics Simulation Studies
Source: PLoS One. 2014 Mar 25;9(3):e92636. doi: 10.1371/journal.pone.0092636 (PMC3965439; doi:10.1371/journal.pone.0092636)

Figure S1 Multiple sequence alignment of 30 F3GT protein sequences. The regions involved in acceptor binding are enclosed in rectangular boxes

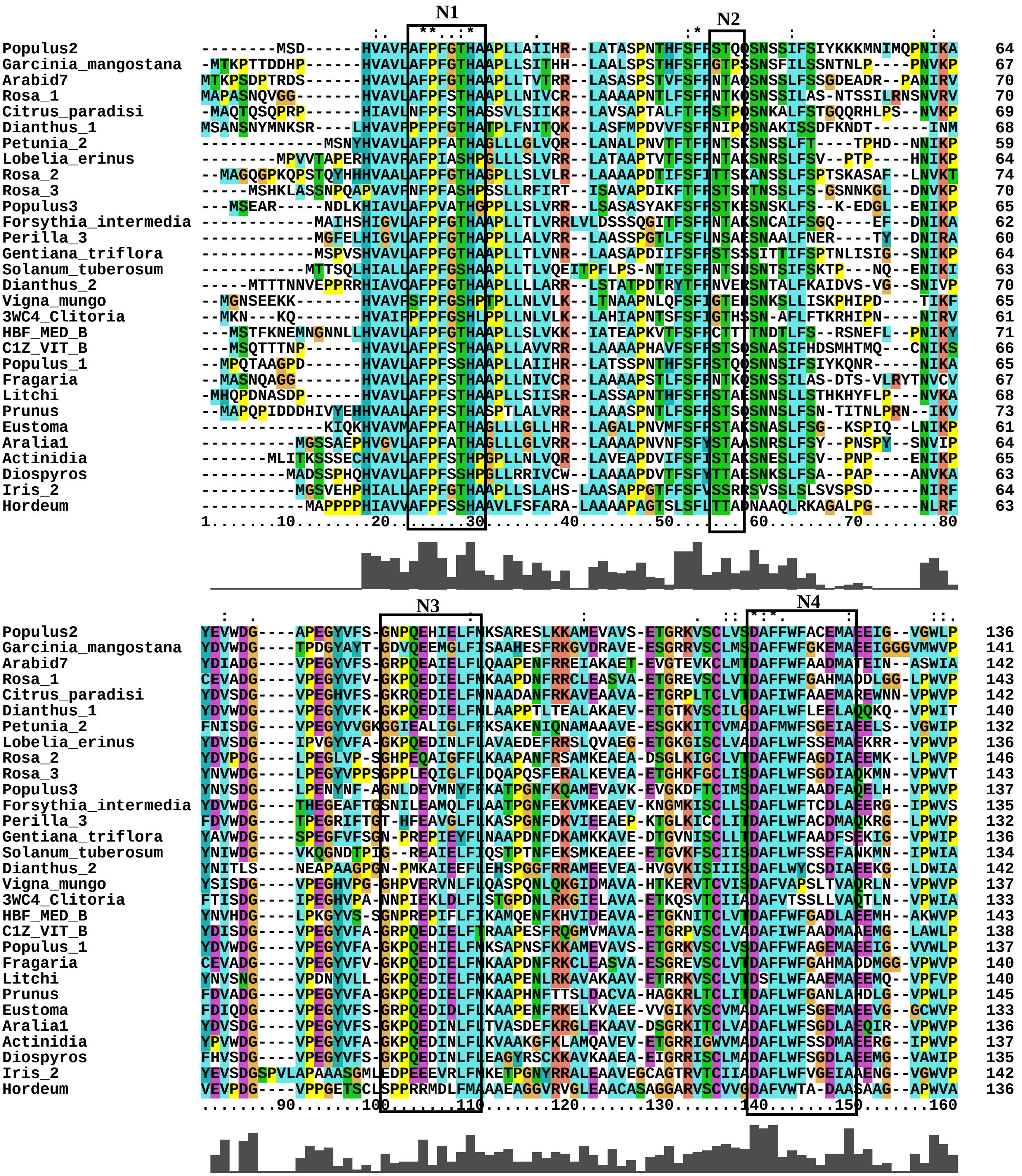

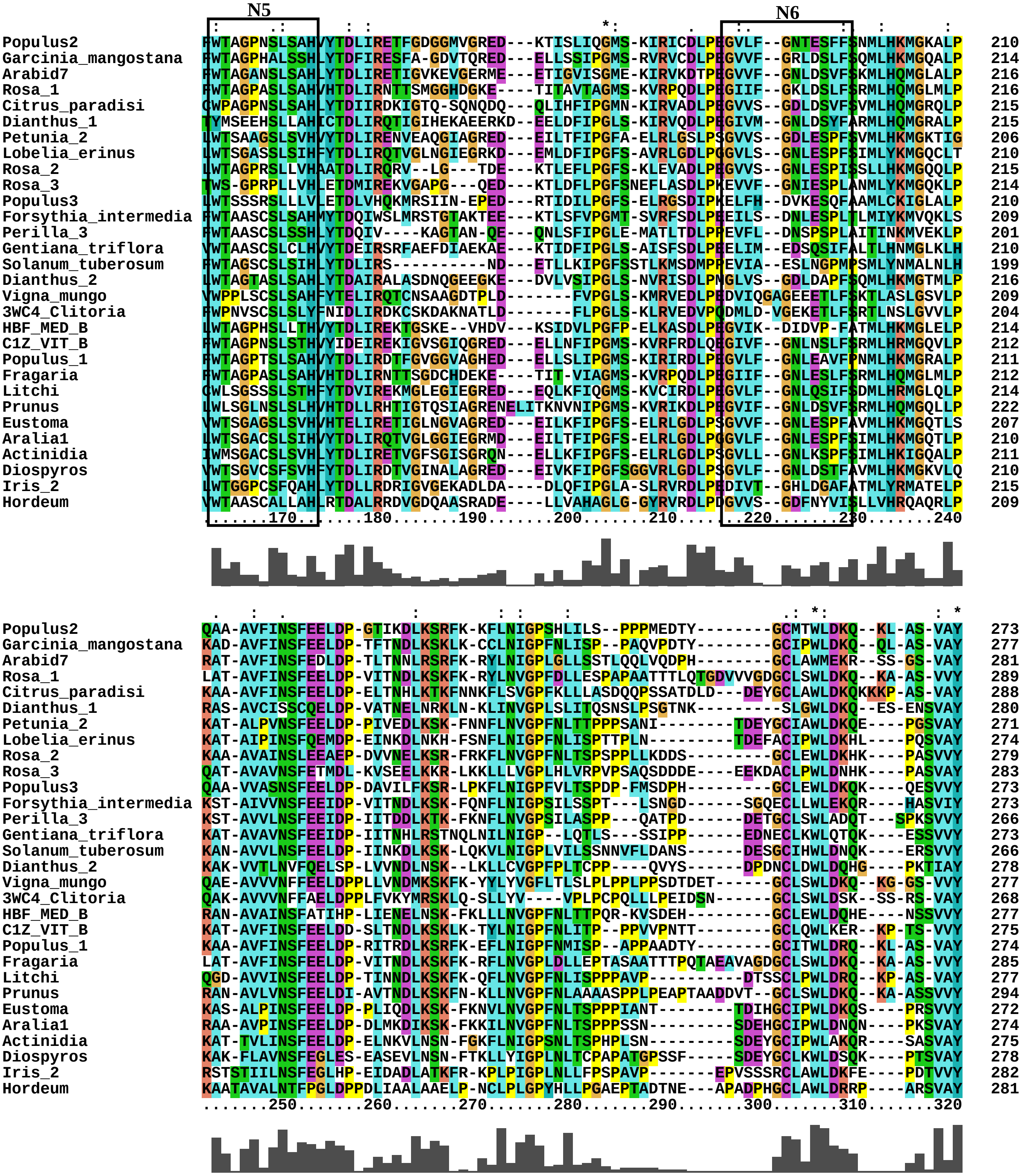
N5
N6
Populus2 FWTAGPNSLSAHVYTDLIRETFGDGGMVGRED---KTISLIQGMS-KIRICDLPEGVLF--GNTESFFSNMLHKMGKALP 210
Garcinia_mangostana FWTAGPHALSSHLYTDFIRESFA-GDVTQRED---ELLSSIPGMS-RVRVCDLPEGVVF--GRLDSLFSQMLHKMGQALP 214
Arabid7 FWTAGANSLSAHLYTDLIRETIGVKEVGERME---ETIGVISGME-KIRVKDTPEGVVF--GNLDSVFSKMLHQMGLALP 216
Rosa_1 FWTAGPASLSAHVHTDLIRNTTSMGGHDGKE----TITAVTAGMS-KVRPQDLPEGIIF--GKLDSLFSRMLHQMGLMLP 216
Citrus_paradisi CWPAGPNSLSAHLYTDIIRDKIGTQ-SQNQDQ---QLIHFIPGMN-KIRVADLPEGVVS--GDLDSVFSVMLHQMGRQLP 215
Dianthus_1 TYMSEEHSLLAHICTDLIRQTIGIHEKAEERKD--EELDFIPGLS-KIRVQDLPEGIVM--GNLDSYFARMLHQMGRALP 215
Petunia_2 LWTSAAGSLSVHVYTDLIRENVEAQGIAGRED---EILTFIPGFA-ELRLGSLPSGVVS--GDLESPFSVMLHKMGKTIG 206
Lobelia_erinus LWTSGASSLSIHFYTDLIRQTVGLNGIEGRKD---EMLDFIPGFS-AVRLGDLPGGVLS--GNLESPFSIMLYKMGQCLT 210
Rosa_2 LWTAGPRSLLVHAATDLIRQRV--LG---TDE---KTLEFLPGFS-KLEVADLPEGVVS--GNLESPISSLLHKMGQQLP 215
Rosa_3 TWS-GPRPLLVHLETDMIREKVGAPG---QED---KTLDFLPGFSNEFLASDLPKEVVF--GNIESPLANMLYKMGQKLP 214
Populus3 LWTSSSRSLLLVLETDLVHQKMRSIIN-EPED---RTIDILPGFS-ELRGSDIPKELFH--DVKESQFAAMLCKIGLALP 210
Forsythia_intermedia FWTAASCSLSAHMYTDQIWSLMRSTGTAKTEE---KTLSFVPGMT-SVRFSDLPEEILS--DNLESPLTLMIYKMVQKLS 209
Perilla_3 FWTAASCSLSSHLYTDQIV----KAGTAN-QE---QNLSFIPGLE-MATLTDLPPEVFL--DNSPSPLAITINKMVEKLP 201
Gentiana_triflora VWTAASCSLCLHVYTDEIRSRFAEFDIAEKAE---KTIDFIPGLS-AISFSDLPEELIM--EDSQSIFALTLHNMGLKLH 210
Solanum_tuberosum FWTAGSCSLSIHLYTDLIRS----------ND---ETLLKIPGFSSTLKMSDMPPEVIA--ESLNGPMPSMLYNMALNLH 199
Dianthus_2 LWTAGTASLSAHLYTDAIRALASDNQGEEGKE---DVLVSIPGLS-NVRISDLPNGLVS--GDLDAPFSQMLHKMGTMLP 216
Vigna_mungo VWPPLSCSLSAHFYTELIRQTCNSAAGDTPLD-------FVPGLS-KMRVEDLPEDVIQGAGEEETLFSKTLASLGSVLP 209
3WC4_Clitoria FWPNVSCSLSLYFNIDLIRDKCSKDAKNATLD-------FLPGLS-KLRVEDVPQDMLD-VGEKETLFSRTLNSLGVVLP 204
HBF_MED_B LWTAGPHSLLTHVYTDLIREKTGSKE--VHDV---KSIDVLPGFP-ELKASDLPEGVIK--DIDVP-FATMLHKMGLELP 214
C1Z_VIT_B FWTAGPNSLSTHVYIDEIREKIGVSGIQGRED---ELLNFIPGMS-KVRFRDLQEGIVF--GNLNSLFSRMLHRMGQVLP 212
Populus_1 FWTAGPTSLSAHVYTDLIRDTFGVGGVAGHED---ELLSLIPGMS-KIRIRDLPEGVLF--GNLEAVFPNMLHKMGRALP 211
Fragaria FWTAGPASLSAHVHTDLIRNTTSGDCHDEKE----TIT-VIAGMS-KVRPQDLPEGIIF--GNLESLFSRMLHQMGLMLP 212
Litchi CWLSGSSSLSTHFYTDVIREKMGLEGIEGRED---EQLKFIQGMS-KVCIRDLPEGVLF--GNLQSIFSDMLHRMGLQLP 214
Prunus LWLSGLNSLSLHVHTDLLRHTIGTQSIAGRENELITKNVNIPGMS-KVRIKDLPEGVIF--GNLDSVFSRMLHQMGQLLP 222
Eustoma VWTSGAGSLSVHVHTELIRETIGLNGVAGRED---EILKFIPGFS-ELRLGDLPSGVVF--GNLESPFAVMLHKMGQTLS 207
Aralia1 LWTSGACSLSIHVYTDLIRQTVGLGGIEGRMD---EILTFIPGFS-ELRLGDLPGGVLF--GNLESPFSIMLHKMGQTLP 210
Actinidia IWMSGACSLSVHLYTDLIRETVGFSGISGRQN---ELLKFIPGFS-ELRLGDLPSGVLL--GNLKSPFSIMLHKIGQALP 211
Diospyros VWTSGVCSFSVHFYTDLIRDTVGINALAGRED---EIVKFIPGFSGGVRLGDLPSGVLF--GNLDSTFAVMLHKMGKVLQ 210
Iris_2 LWTGGPCSFQAHLYTDLLRDRIGVGEKADLDA----DLQFIPGLA-SLRVRDLPEDIVT--GHLDGAFATMLYRMATELP 215
Hordeum VWTAASCALLAHLRTDALRRDVGDQAASRADE----LLVAHAGLG-GYRVRDLPDGVVS--GDFNYVISLLVHRQAQRLP 209
.......170.........180.......190.......200.......210.......220.......230.......240
Populus2 QAA-AVFINSFEELDP-GTIKDLKSRFK-KFLNIGPSHLILS--PPPMEDTY--------GCMTWLDKQ--KL-AS-VAY 273
Garcinia_mangostana KAD-AVFINSFEELDP-TFTNDLKSKLK-CCLNIGPFNLISP--PAQVPDTY--------GCIPWLDKQ--QL-AS-VAY 277
Arabid7 RAT-AVFINSFEDLDP-TLTNNLRSRFK-RYLNIGPLGLLSSTLQQLVQDPH--------GCLAWMEKR--SS-GS-VAY 281
Rosa_1 LAT-AVFINSFEELDP-VITNDLKSKFK-RYLNVGPFDLLESPAPAATTTLQTGDVVVGDGCLSWLDKQ--KA-AS-VVY 289
Citrus_paradisi KAA-AVFINSFEELDP-ELTNHLKTKFNNKFLSVGPFKLLLASDQQPSSATDLD---DEYGCLAWLDKQKKKP-AS-VAY 288
Dianthus_1 RAS-AVCISSCQELDP-VATNELNRKLN-KLINVGPLSLITQSNSLPSGTNK---------SLGWLDKQ--ES-ENSVAY 280
Petunia_2 KAT-ALPVNSFEELDP-PIVEDLKSK-FNNFLNVGPFNLTTPPPSANI--------TDEYGCIAWLDKQE----PGSVAY 271
Lobelia_erinus KAT-AIPINSFQEMDP-EINKDLNKH-FSNFLNIGPFNLISPTTPLN---------TDEFACIPWLDKHL----PQSVAY 274
Rosa_2 KAA-AVAINSLEEAEP-DVVNELKSR-FRKFLNVGPFNLTSPSPPLLKDDS---------GCLEWLDKHK----PASVVY 279
Rosa_3 QAT-AVAVNSFETMDL-KVSEELKKR-LKKLLLVGPLHLVRPVPSAQSDDDE----EEKDACLPWLDNHK----PASVAY 283
Populus3 QAA-VVASNSFEELDP-DAVILFKSR-LPKFLNIGPFVLTSPDP-FMSDPH---------GCLEWLDKQK----QESVVY 273
Forsythia_intermedia KST-AIVVNSFEEIDP-VITNDLKSK-FQNFLNIGPSILSSPT---LSNGD------SGQECLLWLEKQR----HASVIY 273
Perilla_3 KST-AVVLNSFEEIDP-IITDDLKTK-FKNFLNVGPSILASPP---QATPD------DETGCLSWLADQT---SPKSVVY 266
Gentiana_triflora KAT-AVAVNSFEEIDP-IITNHLRSTNQLNILNIGP--LQTLS---SSIPP------EDNECLKWLQTQK----ESSVVY 273
Solanum_tuberosum KAN-AVVLNSFEELDP-IINKDLKSK-LQKVLNIGPLVILSSNNVFLDANS------DESGCIHWLDNQK----ERSVVY 266
Dianthus_2 KAK-VVTLNVFQELSP-LVVNDLNSK--LKLLCVGPFPLTCPP----QVYS------DPDNCLDWLDQHG----PKTIAY 278
Vigna_mungo QAE-AVVVNFFEELDPPLLVNDMKSKFK-YYLYVGFLTLSLPLPPLPPSDTDET------GCLSWLDKQ--KG-GS-VVY 277
3WC4_Clitoria QAK-AVVVNFFAELDPPLFVKYMRSKLQ-SLLYV----VPLPCPQLLLPEIDSN------GCLSWLDSK--SS-RS-VAY 268
HBF_MED_B RAN-AVAINSFATIHP-LIENELNSK-FKLLLNVGPFNLTTPQR-KVSDEH---------GCLEWLDQHE----NSSVVY 277
C1Z_VIT_B KAT-AVFINSFEELDD-SLTNDLKSKLK-TYLNIGPFNLITP--PPVVPNTT--------GCLQWLKER--KP-TS-VVY 275
Populus_1 KAA-AVFINSFEELDP-RITRDLKSRFK-EFLNIGPFNMISP--APPAADTY--------GCITWLDRQ--KL-AS-VAY 274
Fragaria LAT-AVFINSFEELDP-VITNDLKSKFK-RFLNVGPLDLLEPTASAATTTPQTAEAVAGDGCLSWLDKQ--KA-AS-VVY 285
Litchi QGD-AVVINSFEELDP-TINNDLKSKFK-QFLNVGPFNLISPPPAVP----------DTSSCLPWLDRQ--KP-AS-VAY 277
Prunus RAN-AVLVNSFEELDI-AVTNDLKSKFN-KLLNVGPFNLAAAASPPLPEAPTAADDVT--GCLSWLDKQ--KA-ASSVVY 294
Eustoma KAS-ALPINSFEELDP-PLIQDLKSK-FKNVLNVGPFNLTSPPPIANT--------TDIHGCIPWLDKQS----PRSVVY 272
Aralia1 RAA-AVPINSFEELDP-DLMKDIKSK-FKKILNVGPFNLTSPPPSSN---------SDEHGCIPWLDNQN----PKSVAY 274
Actinidia KAT-TVLINSFEELDP-ELNKVLNSN-FGKFLNIGPSNLTSPHPLSN---------SDEYGCIPWLAKQR----SASVAY 275
Diospyros KAK-FLAVNSFEGLES-EASEVLNSN-FTKLLYIGPLNLTCPAPATGPSSF-----SDEYGCLKWLDSQK----PTSVAY 278
Iris_2 RSTSTIILNSFEGLHP-EIDADLATKFR-KPLPIGPLNLLFPSPAVP--------EPVSSSRCLAWLDKFE----PDTVVY 282
Hordeum KAATAVALNTFPGLDPPDLIAALAAELP-NCLPLGPYHLLPGAEPTADTNE---APADPHGCLAWLDRRP----ARSVAY 281
.......250.......260.......270.......280.......290.......300.......310.......320

PSPG MOTIF

C1

```
Populus2             VSFGSVTTPPPHELVALAEALETSETPFIWSLKDNSKVHLPHGFLDRTT---SQGLVVPWSPQLEVLAHRAVGVFVTHCG  350
Garcinia_mangostana  VSFGSATIPLPHELVALAEALEDRKVPFIWSLKDNAKVHLPDGFLETTK---FQGIVIPWAPQAKVLGHKAVGVFITHCG  354
Arabid7              ISFGTVMTPPPGELAAIAEGLESSKVPFVWSLKEKSLVQLPKGFLDRTR---EQGIVVPWAPQVELLKHEATGVFVTHCG  358
Rosa_1               VSFGSVTRPSPEELMALAEALEASRVPFLWSLRNNLMTPKLDEFISKAE---LNGMVVPWVPQPQVLAHGSVGAFVTHCG  366
Citrus_paradisi      VSFGTVATPSPNEIVAIAEALEANKVPFIWSLRHRSQANLPNGFLERTR---SDGIVVDWAPQVNVLAHEAVGVFVTHCG  365
Dianthus_1           VSFGSVARPDATEITALAQALEASQVKFIWSIRDNLKVHLPGGFIENTK---DKGMVVSWVPQTAVLAHKAVGVFITHFG  357
Petunia_2            IGFGTVATPPPNELKAMAEALEESKTPFLWSLKDLFKSFFPEGFLERTS---EYGKIVSWAPQVQVLSHGSVGVFINHCG  348
Lobelia_erinus       IGFGTVATPPPHELVALAEALEESGTPFLWSINENSKKHLPEGFLERTG---ENGKLVPWAPQVQVLAHGSVGVFITHGG  351
Rosa_2               ISFGSVVRPPPHELAAFAEALIESAFPFIWSFRGNPDDILPKGCDKSS----LNGKLVSWAPQVQILEHAAVGVFVTHCG  355
Rosa_3               ISFGSVGALPPMEVAALAEALEEGGFPFLWSFRGNLED-FPKGFIERT----STGRVVPWVNQVQILNHPSIGVFVTHGG  358
Populus3             ISFGSVITLPPQELAELVEALKECKLPFLWSFRGNPKEELPEEFLERTK---EKGKVVSWTPQLKVLRHKAIGVFVTHSG  350
Forsythia_intermedia ISFGTVITPQPREMAGLAEALETGEFPFLWSLRDNAMKLLPDGFLDRTS---KFGMIVSWAPQLKVLENPSVGAFITHCG  350
Perilla_3            ISFGTVITPPENELAALADALEICRFPFLWSLKDYAVKSLPDGFLDRTK---GFGKIVAWAPQQQVLAHRNVGVFVTHCG  343
Gentiana_triflora    LSFGTVINPPPNEMAALASTLESRKIPFLWSLRDEARKHLPENFIDRTS---TFGKIVSWAPQLHVLENPAIGVFVTHCG  350
Solanum_tuberosum    LSFGTVTTLPPNEIIAIAEALEDKKMTFIWSLRDNGVKILPKGFLERTK---EYGKIISWAPQLEILAHRSVGVFVTHCG  343
Dianthus_2           ISFGTVATPPPHEIRALAKALEGSDIPFVWSMSDSVRANLPESFIEKIQNEPKVGKIVSWAPQIKLLGHPSTGVFVTHCG  358
Vigna_mungo          VSFGTVVTPPPHEIVAVAEALEASGFPFLWSLKEHLKGVLPNGFLERTS---ERGKVVGWAPQTQVLGHGSVGVFVTHCG  354
3WC4_Clitoria        VCFGTVVSPPPQEVVAVAEALEESGFPFVWALKESLLSILPKGFVERTS---TRGKVVSWVPQSHVLSHGSVGVFVTHCG  345
HBF_MED_B            ISFGSVVTPPPHELTALAESLEECGFPFIWSFRGDPKEKLPKGFLERTK---TKGKIVAWAPQVEILKHSSVGVFLTHSG  354
C1Z_VIT_B            ISFGTVTTPPPAEVVALSEALEASRVPFIWSLRDKARVHLPEGFLEKTR---GYGMVVPWAPQAEVLAHEAVGAFVTHCG  352
Populus_1            LSFGSITTPPPHELVALAEALETSGVPFIWSLKDNSKVHLPNGFLDRTT---SQGLLVPWTPQMEVLAHKAVGVFITHCG  351
Fragaria             VSFGSVTRPSPEELMALAEALEASRVPFLWSLRDNLKNPQLDEFLSKGK---LNGMVVPWAPQPQVLAHGSVGAFVTHCG  362
Litchi               LGFGSVSRLPPNEIVAVAEALEASKLPFIWSLKKNLQADLPN-----RK---LNGIMVEWAPQLDVLAHNAVGVFINHGG  349
Prunus               VSFGSVARPPEKELMAMAQALEASGVPFLWPLKDSFKTPLLNELLIKA----TNGMVVPWAPQPRVLAHASVGAFVTHCG  370
Eustoma              IGFGTVATPPPPELKALAEALVDTKTPFLWSLKDNMRSHLPEGFSERSG---EIGKIVPWAPQVKILEHDSVGVFINHCG  349
Aralia1              IAFGTVATPPPNELVSLAEALEESGTPFLWSLKDNFKNHLPKGFLERNS---KSGKIVAWAPQIQVLSHDAVGVVITHCG  351
Actinidia            IGFGSVAKPKPDEVVAIAEALEASSTPFLWSLRDTSKQYLPEGFLKRTS---ELGKIVPWAPQVQVLAHSSIGVFITHCG  352
Diospyros            IGFGTVGTLAPDELVALANALEARAMPFLWSIKDSLKKHLPEKLLEKMN---RTGKMVPWTPQVQVLAHVSVGVFITHFG  355
Iris_2               VSFGTVVDLPPSELAELALGLESSGSPFLWSIKDPAKAKLPAGFLDRTRD---RGLLVPWIPQVAVLNHNAVAAFLSHCG  359
Hordeum              VSFGTNATARPDELQELAAGLEASGAPFLWSLRG-VVAAPRGFLERAP-----GLVVPWAPQVGVLRHAAVGAFVTHAG  355
                     .......330.......340.......350.......360.......370.......380.......390.......400
```

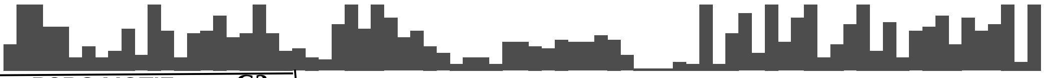

PSPG MOTIF

C2

```
Populus2             WNSLLESIAGGVPMICRPFFGDQRLNGRMIEDVWEIGLKVEDG-VFTKLEVLNSLNKILSHEG-GQKMRENIRALKQLAK  428
Garcinia_mangostana  WNSLLETIVGGVPVICRPFYGDQRLNARMIGDVWKIGVIVNGG-VLAKEAMIDCFDKILLQED-GKQMRGRIKSLKDLAL  432
Arabid7              WNSVLESVSGGVPMICRPFFGDQRLNGRAVEVVWEIGMTIING-VFTKDGFEKCLDKVLVQDD-GKKMKCNAKKLKELAY  436
Rosa_1               WNSVLESLAGGVPMICRPFFGDQKLNARMVEDEWKIGLKLEGG-VFTKNGMLKSLDILLSQKK-GNIMRDTINTFKQLAQ  444
Citrus_paradisi      WGSILESIAAGVPMICRPFFGDQRINGRMMEQVWGVGVAVDGGGICTKEGLLSSLDLILCQEK-GIKIREKVTKLKQLCQ  444
Dianthus_1           HNSIMESIASEVPMIGRPFIGEQKLNGRIVEAKWCIGLVVEGG-VFTKDGVLRSLNKILGSTQ-GEEMRRNIRDLRLMVD  435
Petunia_2            WNSVLESIAAGVPVICRPFFGDHQLNAWMVEKVWKIGVKIEGG-VFTKDGTMLALDLVLSKDKRNTELKQQIGMYKELAL  427
Lobelia_erinus       WNSVVESIGAGVPLIMRPFFGEQQLNTWMVENVWRIGVRVKGG-VFTRSGTVQALEEVLLHQ-KGKKLKEQVVVFKELAH  429
Rosa_2               WNSTLESIVGGVPMICRPFFGDQILNMRTVETVWGIGVGIEGG-VFTKNGAIKALELILRGKA-GNEMREKIKVLKNLAQ  433
Rosa_3               WKSVLESVTCGVPMICRPHFADQTLNMRSVEVVWKIGMRIEGG-VFTKSGAIKVLEQALSLEQ-GKEMRQRVGVLKQLAQ  436
Populus3             WNSVLDSIAGCVPMICRPFFGDQTVNTRTIEAVWGTGLEIEGG-RITKGGLMKALRLIMSTDE-GNKMRKKLQHLQGLAL  428
Forsythia_intermedia WNSILESISFGVPMICRPFFGDQNLNSKMVEDVWKIGVRLEGG-VFTKNGTIEALHSVMLNET-GKAIRENINKLKRKAQ  428
Perilla_3            WNSILESISSCVPLICRPFFGDQKLNSRMVQDSWKIGVRVEGG-VFTKNEAVESLKKLMATEA-GMKIRENVSLLREKAT  421
Gentiana_triflora    WNSTLESIFCRVPVIGRPFFGDQKVNARMVEDVWKIGVGVKGG-VFTEDETTRVLELVLFSDK-GKEMRQNVGRLKEKAK  428
Solanum_tuberosum    WNSILEGISYGVPMICRPFFGDQKLNSRMVESVWEIGLQIEGG-NFTKSGTISALSTFFNEEK-GKVLRKNVEGLKEKAL  421
Dianthus_2           WNSIMESISTGVPLICRPIIGDQELNQRIVEIELKFGIGIEGG-CFTQSGTTNALNQVLASEK-GEEMRKNVEELKKLAE  436
Vigna_mungo          CNSVFESMSNGVPMICRPFFGDHGLTGRMVEDVWEIGVRVEGG-VFTKDGLLKSLRLILVEEE-GNLMKKNAVKVKKTVL  432
3WC4_Clitoria        ANSVMESVSNGVPMICRPFFGDQGIAARVIQDIWEVGVIVEGK-VFTKNGFVKSLNLILVQED-GKKIRDNALKVKQIVQ  423
HBF_MED_B            WNSVLECIVGGVPMISRPFFGDQGLNTILTESVLEIGVGVDNG-VLTKESIKKALELTMSSEK-GGIMRQKIVKLKESAF  432
C1Z_VIT_B            WNSLWESVAGGVPLICRPFFGDQRLNGRMVEDVLEIGVRIEGG-VFTKSGLMSCFDQILSQEK-GKKLRENLRALRETAD  430
Populus_1            WNSLLESIAGGVPMICRPFFGDQRLNGRMVEDAWKIGLQVEDG-VFRKHGVLNSLDKVLSQDS-GEEMRENIRALQQLAK  429
Fragaria             WNSVLESVAGGVPLICRPFFGDQKLNARMVEDVWKIGLRLEGG-VFTKNGMLKSLDMLLSQDK-GTKMKNKIHTLKQLAQ  440
Litchi               WSSLMESMACGVPMIIRPFFGDQRLNARMVQDEWKIGVSVEGG-IITKRGFLRSLDLILWQEN-GKKMRENVRKFKQLAE  427
Prunus               WSSLLETIAGGVPMICRPFFGDQRVNARLVEDVLEIGATVEDG-VFTKHGMIKYFDQVLSQQR-GKKLRENINTVKLLAR  448
Eustoma              WNSVMESIAAGVPIICRPFFGDHQLNSWMVEKVWQIGLRIEGG-VFTKDATNRALEVVFSHEK-GKKIKEQVVAYKKLAL  427
Aralia1              WNSVVESIAAGVPVICRPFFGDHHINTWMVENVWKIGVRIEGG-VFTRTGTMNALEQVLLSQEKGKKLKEQITVFKELAL  430
Actinidia            WNSVLETIAGGVPMIGRPFFGDHPMNTWMVENVWKIGVRVEGG-VFTKSSTMRALELVLSHE-KGKKLKDQIGHLRELAL  430
Diospyros            WNSVLESIAAGVPLICRPLFGDHQLNGWMAEHVWRVGVKVEGG-VFTKDGTIRALDLVLLEAE-GKKLKENAEWFKEIAH  433
Iris_2               WNSVLESMTCGVPMVCRPFLGDQMLNSKVVSQVWKVGVRLHNG-PMTSTNVAEAIKTVVAGDE-GKNMRDRAAKMREKAT  437
Hordeum              WASVMEGVSSGVPMACRPFFGDQTMNARSVASVWGFGTAFD-G-PMTRGAVANAVATLLRGED-GERMRAKAQELQAMVG  432
                     .......410.......420.......430.......440.......450.......460.......470.......480
```

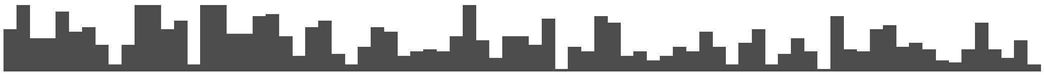

```
                      :     ^ .   :
Populus2             KAIGPN-GSSINNFIALSNLVFNTKI---    453
Garcinia_mangostana  AATAYK-GSSSDNMRELSRLVSSPCK---    457
Arabid7              EAVSSK-GRSSENFRGLLDAVVNII----    460
Rosa_1               QAVEPK-GSSTRNFESLLEVISTAN----    468
Citrus_paradisi      NAIGPG-GSSMQNLDALVDMISRSY----    468
Dianthus_1           KALSPD-GSCNTNLKHLVDMIVTSN----    459
Petunia_2            NAVGPS-GSSAENFKKLVDIITSCN----    451
Lobelia_erinus       KAVGPS-GSSTENFKTLIEIITNMEQSAT    457
Rosa_2               SAVEGD-GSSSKAFNCLVDIATNHEEV--    459
Rosa_3               ETVGPN-GSSAQDLKVW------------    452
Populus3             DAVQSS-GSSTKNFETLLEVVAK------    450
Forsythia_intermedia NAVKFD-GTSTKNFRALLELIKSPRGI--    454
Perilla_3            AAVKPE-GSSSQNFKKLLEIIGAAESS--    447
Gentiana_triflora    DAVKAN-GSSTRNFESLLAAFNKLDS---    453
Solanum_tuberosum    EAVKLDNGSSIENFKVLVELVKCHKLT--    448
Dianthus_2           ESVKQG-GSSNENFNALIELITSS-----    459
Vigna_mungo          DAAGAQ-GKAAQDFNTLVELVSRS-----    455
3WC4_Clitoria        DAVGPH-GQAAEDFNTLVEVISSS-----    446
HBF_MED_B            KAVEQN-GTSAMDFTTLIQIVTS------    454
C1Z_VIT_B            RAVGPK-GSSTENFITLVDLVSKPKDV--    456
Populus_1            KAIGPN-GSSINNFVSLSDLV--------    449
Fragaria             QAVEPK-GSSTRNFESLLEMATTN-----    463
Litchi               TAVGPQ-GSSMKNFKALVDIVSRPNDA--    453
Prunus               QSVEPK-GGSAQNFKLLLDVISRSTKV--    474
Eustoma              EAVGPN-GSSTKGFQAMVEVISTCQ----    451
Aralia1              KAVGPN-GSSTQNFKRLLEVITT------    452
Actinidia            KAVGPK-GSSSQNFNNLLEVITGHNL---    455
Diospyros            KAAGKN-GSSTRSFDSLVKSVRAQKDL--    459
Iris_2               GSVRPD-GSSVRNLNTLLEIVFAR-----    460
Hordeum              KAFEPD-GGCRKNFDEFVEIVCRV-----    455
                     .......490.......500.........
```

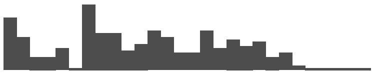

Supplement: Figure S1 — Multiple sequence alignment of 30 F3GT protein sequences. (PDF) [file pone.0092636.s001.pdf]
