# Supplementary material for: In-Silico Analysis of Binding Site Features and Substrate Selectivity in Plant Flavonoid-3-O Glycosyltransferases (F3GT) through Molecular Modeling, Docking and Dynamics Simulation Studies
Source: PLoS One. 2014 Mar 25;9(3):e92636. doi: 10.1371/journal.pone.0092636 (PMC3965439; doi:10.1371/journal.pone.0092636)
Supplement: Figure S2 — Multiple sequence alignment between query (30F3GTs) and template (PDB ID: 2C1Z, 3HBF and 3WC4). (PDF) [file pone.0092636.s002.pdf]

Figure S2 Multiple sequence alignment between query (30F3GTs) and template (PDB ID: 2C1Z, 3HBF and 3WC4) generated by ClustalX

```
                                 :.  :     *********.**..*** :*:::*. **.. ***:.*: :* ::*          *** * : **:*
Actinidia                   MLITKSSSE----CHVAVLAFPFSTHPGPLLNLVQRLAVEAPDVIFSFISTAKSNESLFS---VPNPENIKPYPVWDGVP    73
2C1Z_A|PDBID|CHAIN|SEQUENCE --MSQTTTN----PHVAVLAFPFSTHAAPLLAVVRRLAAAAPHAVFSFFSTSQSNASIFHDSMHTMQCNIKSYDISDGVP    74
3HBF_A|PDBID|CHAIN|SEQUENCE MSTFKNEMNGNNLLHVAVLAFPFGTHAAPLLSLVKKIATEAPKVTFSFFCTTTTNDTLFSRSNEFLP-NIKYYNVHDGLP    79
                            1.......10........20........30........40........50........60........70........80

                            :*** :*.*:* * ** :.   :.*:  :  . .***: :  :::***:**.:*:* *    *:*:* :*. ** .*:* *
Actinidia                   EGYVFAGKPQEDINLFLKVAAKGFKLAMQAVEVETGRRIGWVMADAFLWFSSDMAEERGIPWVPIWMSGACSLSVHLYTD   153
2C1Z_A|PDBID|CHAIN|SEQUENCE EGYVFAGRPQEDIELFTRAAPESFRQGMVMAVAETGRPVSCLVADAFIWFAADMAAEMGVAWLPFWTAGPNSLSTHVYID   154
3HBF_A|PDBID|CHAIN|SEQUENCE KGYVSSGNPREPIFLFIKAMQENFKHVIDEAVAETGKNITCLVTDAFFWFGADLAEEMHAKWVPLWTAGPHSLLTHVYTD   159
                            ........90.......100.......110.......120.......130.......140.......150.......160

                             ***. * . : . :.  :..:**:.:::  ** .*::  ::.  *: ***::*  **:*.:* ****  :.  : : *:
Actinidia                   LIRETVGFSGISGRQNELLKFIPGFSELRLGDLPSGVLLGNLKSPFSIMLHKIGQALPKATTVLINSFEELDPELNKVLN   233
2C1Z_A|PDBID|CHAIN|SEQUENCE EIREKIGVSGIQGREDELLNFIPGMSKVRFRDLQEGIVFGNLNSLFSRMLHRMGQVLPKATAVFINSFEELDDSLTNDLK   234
3HBF_A|PDBID|CHAIN|SEQUENCE LIREKTGSKEVHDVKS--IDVLPGFPELKASDLPEGVIK-DIDVPFATMLHKMGLELPRANAVAINSFATIHPLIENELN   236
                            .......170.......180.......190.......200.......210.......220.......230.......240

                            *::   **:** ** :*      .    **: ** ::. :**.**.**:*..* * *:.*::*:**  .   **:**:*. .:
Actinidia                   SNFGKFLNIGPSNLTSPHPLSNSDEYGCIPWLAKQRSASVAYIGFGSVAKPKPDEVVAIAEALEASSTPFLWSLRDTSKQ   313
2C1Z_A|PDBID|CHAIN|SEQUENCE SKLKTYLNIGPFNLITPPPVVPN-TTGCLQWLKERKPTSVVYISFGTVTTPPPAEVVALSEALEASRVPFIWSLRDKARV   313
3HBF_A|PDBID|CHAIN|SEQUENCE SKFKLLLNVGPFNLTTPQRKVSD-EHGCLEWLDQHENSSVVYISFGSVVTPPPHELTALAESLEECGFPFIWSFRGDPKE   315
                            .......250.......260.......270.......280.......290.......300.......310.......320

                             **:***::*   * :*.****.::* *.::*.*:**.****: * :.****:* ******: :*   :.*.* :*** ::
Actinidia                   YLPEGFLKRTSELGKIVPWAPQVQVLAHSSIGVFITHCGWNSVLETIAGGVPMIGRPFFGDHPMNTWMVENVWKIGVRVE   393
2C1Z_A|PDBID|CHAIN|SEQUENCE HLPEGFLEKTRGYGMVVPWAPQAEVLAHEAVGAFVTHCGWNSLWESVAGGVPLICRPFFGDQRLNGRMVEDVLEIGVRIE   393
3HBF_A|PDBID|CHAIN|SEQUENCE KLPKGFLERTKTKGKIVAWAPQVEILKHSSVGVFLTHSGWNSVLECIVGGVPMISRPFFGDQGLNTILTESVLEIGVGVD   395
                            .......330.......340.......350.......360.......370.......380.......390.......400

                            .**:**..    .::   :* ***   :::::   *:* * :**   :*:*: :*  .*:::::
Actinidia                   GGVFTKSSTMRALELVLSHEKGKKLKDQIGHLRELALKAVGPKGSSSQNFNNLLEVITG-HNL   455
2C1Z_A|PDBID|CHAIN|SEQUENCE GGVFTKSGLMSCFDQILSQEKGKKLRENLRALRETADRAVGPKGSSTENFITLVDLVSKPKDV   456
3HBF_A|PDBID|CHAIN|SEQUENCE NGVLTKESIKKALELTMSSEKGGIMRQKIVKLKESAFKAVEQNGTSAMDFTTLIQIVTS----   454
                            .......410.......420.......430.......440.......450.......460...
```

```
                                .:   .    *********.*******::.:::*: :* ..**** *: :* ::*  .       **: *:: **:
Arabid7                     MTKPSDPTRDS---HVAVLAFPFGTHAAPLLTVTRRLASASPSTVFSFFNTAQSNSSLFSSGDEADRPANIRVYDIADGV    77
2C1Z_A|PDBID|CHAIN|SEQUENCE ---MSQTTTNP---HVAVLAFPFSTHAAPLLAVVRRLAAAAPHAVFSFFSTSQSNASIFHDS-MHTMQCNIKSYDISDGV    73
3HBF_A|PDBID|CHAIN|SEQUENCE MSTFKNEMNGNNLLHVAVLAFPFGTHAAPLLSLVKKIATEAPKVTFSFFCTTTTNDTLFSRS--NEFLPNIKYYNVHDGL    78
                            1.......10........20........30........40........50........60........70........80

                            *:*** :*.*:* * ** :*  *.*:: :  * :*.*  :.**::***:**.**:* *: . *:.:****.:** :*:*
Arabid7                     PEGYVFSGRPQEAIELFLQAAPENFRREIAKAETEVGTEVKCLMTDAFFWFAADMATEINASWIAFWTAGANSLSAHLYT   157
2C1Z_A|PDBID|CHAIN|SEQUENCE PEGYVFAGRPQEDIELFTRAAPESFRQGMVMAVAETGRPVSCLVADAFIWFAADMAAEMGVAWLPFWTAGPNSLSTHVYI   153
3HBF_A|PDBID|CHAIN|SEQUENCE PKGYVSSGNPREPIFLFIKAMQENFKHVIDEAVAETGKNITCLVTDAFFWFGADLAEEMHAKWVPLWTAGPHSLLTHVYT   158
                            ........90.......100.......110.......120.......130.......140.......150.......160

                            * ***. * . :    .  :..:.*: :::  *  **::  :::  *: ***:**  **:*.** ****  :.  : *:*
Arabid7                     DLIRETIGVKEVGERMEETIGVISGMEKIRVKDTPEGVVFGNLDSVFSKMLHQMGLALPRATAVFINSFEDLDPTLTNNL   237
2C1Z_A|PDBID|CHAIN|SEQUENCE DEIREKIGVSGIQGREDELLNFIPGMSKVRFRDLQEGIVFGNLNSLFSRMLHRMGQVLPKATAVFINSFEELDDSLTNDL   233
3HBF_A|PDBID|CHAIN|SEQUENCE DLIREKTGSKEVHDVKS--IDVLPGFPELKASDLPEGVIK-DIDVPFATMLHKMGLELPRANAVAINSFATIHPLIENEL   235
                            .......170.......180.......190.......200.......210.......220.......230.......240

                            .*::*  **:**:.* :.     * :  *** *:.::.  **.*****:* **** *:.*::*.** . .**:**:: ..
Arabid7                     RSRFKRYLNIGPLGLLSSTLQQLVQDPHGCLAWMEKRSSGSVAYISFGTVMTPPPGELAAIAEGLESSKVPFVWSLKEKS   317
2C1Z_A|PDBID|CHAIN|SEQUENCE KSKLKTYLNIGPFNLITP--PPVVPNTTGCLQWLKERKPTSVVYISFGTVTTPPPAEVVALSEALEASRVPFIWSLRDKA   311
3HBF_A|PDBID|CHAIN|SEQUENCE NSKFKLLLNVGPFNLTTP--QRKVSDEHGCLEWLDQHENSSVVYISFGSVVTPPPHELTALAESLEECGFPFIWSFRGDP   313
                            .......250.......260.......270.......280.......290.......300.......310.......320

                              :**:***::*:  * :*.****.*:* *.:.*.*:**.****: *.: ****:*.******* **    .* * ***:
Arabid7                     LVQLPKGFLDRTREQGIVVPWAPQVELLKHEATGVFVTHCGWNSVLESVSGGVPMICRPFFGDQRLNGRAVEVVWEIGMT   397
2C1Z_A|PDBID|CHAIN|SEQUENCE RVHLPEGFLEKTRGYGMVVPWAPQAEVLAHEAVGAFVTHCGWNSLWESVAGGVPLICRPFFGDQRLNGRMVEDVLEIGVR   391
3HBF_A|PDBID|CHAIN|SEQUENCE KEKLPKGFLERTKTKGKIVAWAPQVEILKHSSVGVFLTHSGWNSVLECIVGGVPMISRPFFGDQGLNTILTESVLEIGVG   393
                            .......330.......340.......350.......360.......370.......380.......390.......400

                            : .**:**..: ..::  : .:.*  :: :   *:* * .**  :* *: :*  *:: * .
Arabid7                     IINGVFTKDGFEKCLDKVLVQDDGKKMKCNAKKLKELAYEAVSSKGRSSENFRGLLDAVVNII--   460
2C1Z_A|PDBID|CHAIN|SEQUENCE IEGGVFTKSGLMSCFDQILSQEKGKKLRENLRALRETADRAVGPKGSSTENFITLVDLVSKPKDV   456
3HBF_A|PDBID|CHAIN|SEQUENCE VDNGVLTKESIKKALELTMSSEKGGIMRQKIVKLKESAFKAVEQNGTSAMDFTTLIQIVTS----   454
                            .......410.......420.......430.......440.......450.......460.....
```

```
                                          .   **.******.***. **.:*:::*: **:. ***:.*: :* ::*     :    *:  *:: **:*
Aralia1                           -------MGSSAEPHVGVLAFPFATHAGLLLGLVRRLAAAAPNVNFSFYSTAASNRSLFS-YPNSPYSNVIPYDVSDGVP    72
2C1Z_A|PDBID|CHAIN|SEQUENCE       ------MSQTTTNPHVAVLAFPFSTHAAPLLAVVRRLAAAAPHAVFSFFSTSQSNASIFHDSMHTMQCNIKSYDISDGVP    74
3HBF_A|PDBID|CHAIN|SEQUENCE       MSTFKNEMNGNNLLHVAVLAFPFGTHAAPLLSLVKKIATEAPKVTFSFFCTTTTNDTLFS-RSNEFLPNIKYYNVHDGLP    79
                                  1.......10........20........30........40........50........60........70........80

                                  :*** :*.*:* * **   .   :.*:: :   *..::*: ::***:***:**..*:* ::  . *:*:**:*.  **   *** *
Aralia1                           EGYVFSGKPQEDINLFLTVASDEFKRGLEKAAVDSGRKITCLVADAFLWFSGDLAEQIRVPWVPLWTSGACSLSIHVYTD   152
2C1Z_A|PDBID|CHAIN|SEQUENCE       EGYVFAGRPQEDIELFTRAAPESFRQGMVMAVAETGRPVSCLVADAFIWFAADMAAEMGVAWLPFWTAGPNSLSTHVYID   154
3HBF_A|PDBID|CHAIN|SEQUENCE       KGYVSSGNPREPIFLFIKAMQENFKHVIDEAVAETGKNITCLVTDAFFWFGADLAEEMHAKWVPLWTAGPHSLLTHVYTD   159
                                  ........90.......100.......110.......120.......130.......140.......150.......160

                                   **:. *   :..  .  :  .:**:.::: **  *::  :::  *: ***:** **:* ** ****  :.  : ::::
Aralia1                           LIRQTVGLGGIEGRMDEILTFIPGFSELRLGDLPGGVLFGNLESPFSIMLHKMGQTLPRAAAVPINSFEELDPDLMKDIK   232
2C1Z_A|PDBID|CHAIN|SEQUENCE       EIREKIGVSGIQGREDELLNFIPGMSKVRFRDLQEGIVFGNLNSLFSRMLHRMGQVLPKATAVFINSFEELDDSLTNDLK   234
3HBF_A|PDBID|CHAIN|SEQUENCE       LIREKTGSKEVHDVKS--IDVLPGFPELKASDLPEGVIK-DIDVPFATMLHKMGLELPRANAVAINSFATIHPLIENELN   236
                                  .......170.......180.......190.......200.......210.......220.......230.......240

                                  **:*  **:***** :*      .:  **: **.::: .**.**:**:*.**** *:.:*:*:**  .  **:**::.. :
Aralia1                           SKFKKILNVGPFNLTSPPPSSNSDEHGCIPWLDNQNPKSVAYIAFGTVATPPPNELVSLAEALEESGTPFLWSLKDNFKN   312
2C1Z_A|PDBID|CHAIN|SEQUENCE       SKLKTYLNIGPFNLITPPP-VVPNTTGCLQWLKERKPTSVVYISFGTVTTPPPAEVVALSEALEASRVPFIWSLRDKARV   313
3HBF_A|PDBID|CHAIN|SEQUENCE       SKFKLLLNVGPFNLTTPQR-KVSDEHGCLEWLDQHENSSVVYISFGSVVTPPPHELTALAESLEECGFPFIWSFRGDPKE   315
                                  .......250.......260.......270.......280.......290.......300.......310.......320

                                  :**:****:.   * :*.**** ::* *.:**..:** ****: *.:..***:*.******: :*  :.*.* :*** ::
Aralia1                           HLPKGFLERNSKSGKIVAWAPQIQVLSHDAVGVVITHGGWNSVVESIAAGVPVICRPFFGDHHINTWMVENVWKIGVRIE   392
2C1Z_A|PDBID|CHAIN|SEQUENCE       HLPEGFLEKTRGYGMVVPWAPQAEVLAHEAVGAFVTHCGWNSLWESVAGGVPLICRPFFGDQRLNGRMVEDVLEIGVRIE   393
3HBF_A|PDBID|CHAIN|SEQUENCE       KLPKGFLERTKTKGKIVAWAPQVEILKHSSVGVFLTHSGWNSVLECIVGGVPMISRPFFGDQGLNTILTESVLEIGVGVD   395
                                  .......330.......340.......350.......360.......370.......380.......390.......400

                                  .**:*: .  ..::  :  .***  :::::  ::* * :**  :*:*: :*  *:::::.
Aralia1                           GGVFTRTGTMNALEQVLLSQEKGKKLKEQITVFKELALKAVGPNGSSTQNFKRLLEVITT----   452
2C1Z_A|PDBID|CHAIN|SEQUENCE       GGVFTKSGLMSCFDQILS-QEKGKKLRENLRALRETADRAVGPKGSSTENFITLVDLVSKPKDV   456
3HBF_A|PDBID|CHAIN|SEQUENCE       NGVLTKESIKKALELTMS-SEKGGIMRQKIVKLKESAFKAVEQNGTSAMDFTTLIQIVTS----   454
                                  .......410.......420.......430.......440.......450.......460....
```

```
                                         :    :    *:*** ***.***:.:*:::::*. **  . *:**.*. :* ::*    .   .  :   *:* *:: **:
Citrus_paradisi                 ----MAQTQSQPRPHIAVLNFPFSTHASSVLSIIKRLAVSAPTALFTFFSTPQSNKALFSTGQQRHLPSNVKPYDVSDGV    76
2C1Z_A|PDBID|CHAIN|SEQUENCE ----MSQTTTN--PHVAVLAFPFSTHAAPLLAVVRRLAAAAPHAVFSFFSTSQSNASIFHDS-MHTMQCNIKSYDISDGV    73
3HBF_A|PDBID|CHAIN|SEQUENCE MSTFKNEMNGNNLLHVAVLAFPFGTHAAPLLSLVKKIATEAPKVTFSFFCTTTTNDTLFSRS--NEFLPNIKYYNVHDGL    78
                            1.......10........20........30........40........50........60........70........80

                            *:*:* :*. :* * ** .*    .*:: :  ******: ::***:***:**.*::* *    . *:* *.***:** :*:*
Citrus_paradisi             PEGHVFSGKRQEDIELFMNAADANFRKAVEAAVAETGRPLTCLVTDAFIWFAAEMAREWNNVPWVPCWPAGPNSLSAHLY   156
2C1Z_A|PDBID|CHAIN|SEQUENCE PEGYVFAGRPQEDIELFTRAAPESFRQGMVMAVAETGRPVSCLVADAFIWFAADMAAEMG-VAWLPFWTAGPNSLSTHVY   152
3HBF_A|PDBID|CHAIN|SEQUENCE PKGYVSSGNPREPIFLFIKAMQENFKHVIDEAVAETGKNITCLVTDAFFWFGADLAEEMH-AKWVPLWTAGPHSLLTHVY   157
                            ........90.......100.......110.......120.......130.......140.......150.......160

                             * **:* *  .  : :: : :..:**: :::  ** **::  :::  *: ***:**  **:* ** ****  :.  : *.
Citrus_paradisi             TDIIRDKIGTQ-SQNQDQQLIHFIPGMNKIRVADLPEGVVSGDLDSVFSVMLHQMGRQLPKAAAVFINSFEELDPELTNH   235
2C1Z_A|PDBID|CHAIN|SEQUENCE IDEIREKIGVSGIQGREDELLNFIPGMSKVRFRDLQEGIVFGNLNSLFSRMLHRMGQVLPKATAVFINSFEELDDSLTND   232
3HBF_A|PDBID|CHAIN|SEQUENCE TDLIREKTGSK--EVHDVKSIDVLPGFPELKASDLPEGVIK-DIDVPFATMLHKMGLELPRANAVAINSFATIHPLIENE   234
                            .......170.......180.......190.......200.......210.......220.......230.......240

                            *::*::   *.:***:*    .    ..       *** **.:  :: :**.*:***:*.**.* *:.*::*:**   .*
Citrus_paradisi             LKTKFNNKFLSVGPFKLLLASDQQPSSATDLDDEYGCLAWLDKQKKKPASVAYVSFGTVATPSPNEIVAIAEALEANKVP   315
2C1Z_A|PDBID|CHAIN|SEQUENCE LKSKLK-TYLNIGPFNLITPPPVVPNTT-------GCLQWLKE--RKPTSVVYISFGTVTTPPPAEVVALSEALEASRVP   302
3HBF_A|PDBID|CHAIN|SEQUENCE LNSKFK-LLLNVGPFNLTTPQRKVSDEH-------GCLEWLDQ--HENSSVVYISFGSVVTPPPHELTALAESLEECGFP   304
                            .......250.......260.......270.......280.......290.......300.......310.......320

                            ****:*   .: :**:****:*:  * :* ****.::* *.:**.*:**.**.*: *.:..***:* ******* :*  :
Citrus_paradisi             FIWSLRHRSQANLPNGFLERTRSDGIVVDWAPQVNVLAHEAVGVFVTHCGWGSILESIAAGVPMIGRPFFGDQRINGRMM   395
2C1Z_A|PDBID|CHAIN|SEQUENCE FIWSLRDKARVHLPEGFLEKTRGYGMVVPWAPQAEVLAHEAVGAFVTHCGWNSLWESVAGGVPLICRPFFGDQRLNGRMV   382
3HBF_A|PDBID|CHAIN|SEQUENCE FIWSFRGDPKEKLPKGFLERTKTKGKIVAWAPQVEILKHSSVGVFLTHSGWNSVLECIVGGVPMISRPFFGDQGLNTILT   384
                            .......330.......340.......350.......360.......370.......380.......390.......400

                            *.*  :** ::.* : **..: ..::  :..***  :*:::  *:: . .*:   *:*  :: :*:::::
Citrus_paradisi             EQVWGVGVAVDGGGICTKEGLLSSLDLILCQEKGIKIREKVTKLKQLCQNAIGPGGSSMQNLDALVDMISRSY--   468
2C1Z_A|PDBID|CHAIN|SEQUENCE EDVLEIGVRIEGG-VFTKSGLMSCFDQILSQEKGKKLRENLRALRETADRAVGPKGSSTENFITLVDLVSKPKDV   456
3HBF_A|PDBID|CHAIN|SEQUENCE ESVLEIGVGVDNG-VLTKESIKKALELTMSSEKGGIMRQKIVKLKESAFKAVEQNGTSAMDFTTLIQIVTS----   454
                            .......410.......420.......430.......440.......450.......460.......470.....
```

```
                            **  .      .   ****:.***.***:**: :.:::*:  *...****   . :*  .:      .     .*: *:: **:*
Dianthus_1                  MSANSNYMN-KSRLHVAVFPFPFGTHATPLFNITQKLASFMPDVVFSFFNIPQSNAKISSDFKN---DTINMYDVWDGVP    76
2C1Z_A|PDBID|CHAIN|SEQUENCE MSQTT------TNPHVAVLAFPFSTHAAPLLAVVRRLAAAAPHAVFSFFSTSQSNASIFHDSMHTMQCNIKSYDISDGVP    74
3HBF_A|PDBID|CHAIN|SEQUENCE MSTFKNEMNGNNLLHVAVLAFPFGTHAAPLLSLVKKIATEAPKVTFSFFCTTTTNDTLFSRSNE-FLPNIKYYNVHDGLP    79
                            1.......10........20........30........40........50........60........70........80

                            :***   *.*:* * **   *    .:  .  :   *  .***   ::*::  ***:**   ::*  :   .  *:.  :  :   :**  :*:   *
Dianthus_1                  EGYVFKGKPQEDIELFMLAAPPTLTEALAKAEVETGTKVSCILGDAFLWFLEELAQQKQVPWITTYMSEEHSLLAHICTD   156
2C1Z_A|PDBID|CHAIN|SEQUENCE EGYVFAGRPQEDIELFTRAAPESFRQGMVMAVAETGRPVSCLVADAFIWFAADMAAEMGVAWLPFWTAGPNSLSTHVYID   154
3HBF_A|PDBID|CHAIN|SEQUENCE KGYVSSGNPREPIFLFIKAMQENFKHVIDEAVAETGKNITCLVTDAFFWFGADLAEEMHAKWVPLWTAGPHSLLTHVYTD   159
                            ........90.......100.......110.......120.......130.......140.......150.......160

                             **:.  *      .   :.   :::.:**:.:::   ** **::   :::   *:  ***:**   **:*.** *.*    :.     *:*
Dianthus_1                  LIRQTIGIHEKAEERKDEELDFIPGLSKIRVQDLPEGIVMGNLDSYFARMLHQMGRALPRASAVCISSCQELDPVATNEL   236
2C1Z_A|PDBID|CHAIN|SEQUENCE EIREKIGVSG-IQGREDELLNFIPGMSKVRFRDLQEGIVFGNLNSLFSRMLHRMGQVLPKATAVFINSFEELDDSLTNDL   233
3HBF_A|PDBID|CHAIN|SEQUENCE LIREKTGSKE-VHDVKS--IDVLPGFPELKASDLPEGVIK-DIDVPFATMLHKMGLELPRANAVAINSFATIHPLIENEL   235
                            .......170.......180.......190.......200.......210.......220.......230.......240

                            : *::  :*:**:.* *    :..     .* **.:::   .**.*:***:*.  *  .  *:.**:::**  .  .  ****:*..
Dianthus_1                  NRKLNKLINVGPLSLITQSNSLPSGTNKSLGWLDKQESENSVAYVSFGSVARPDATEITALAQALEASQVKFIWSIRDNL   316
2C1Z_A|PDBID|CHAIN|SEQUENCE KSKLKTYLNIGPFNLITPPPVVPN-TTGCLQWLKERKP-TSVVYISFGTVTTPPPAEVVALSEALEASRVPFIWSLRDKA   311
3HBF_A|PDBID|CHAIN|SEQUENCE NSKFKLLLNVGPFNLTTPQRKVSD-EHGCLEWLDQHEN-SSVVYISFGSVVTPPPHELTALAESLEECGFPFIWSFRGDP   313
                            .......250.......260.......270.......280.......290.......300.......310.......320

                            : :** **:*.*:   * :*.*.**.  :* *.:**.*:** * **: *.:..  **:* ***:*:* **   :.*     **:
Dianthus_1                  KVHLPGGFIENTKDKGMVVSWVPQTAVLAHKAVGVFITHFGHNSIMESIASEVPMIGRPFIGEQKLNGRIVEAKWCIGLV   396
2C1Z_A|PDBID|CHAIN|SEQUENCE RVHLPEGFLEKTRGYGMVVPWAPQAEVLAHEAVGAFVTHCGWNSLWESVAGGVPLICRPFFGDQRLNGRMVEDVLEIGVR   391
3HBF_A|PDBID|CHAIN|SEQUENCE KEKLPKGFLERTKTKGKIVAWAPQVEILKHSSVGVFLTHSGWNSVLECIVGGVPMISRPFFGDQGLNTILTESVLEIGVG   393
                            .......330.......340.......350.......360.......370.......380.......390.......400

                            ::.**:**..:  .::  :.. :*  :*.::  *:  . :*:  .*:.  ::  *:::: .
Dianthus_1                  VEGGVFTKDGVLRSLNKILGSTQGEEMRRNIRDLRLMVDKALSPDGSCNTNLKHLVDMIVTSN--   459
2C1Z_A|PDBID|CHAIN|SEQUENCE IEGGVFTKSGLMSCFDQILSQEKGKKLRENLRALRETADRAVGPKGSSTENFITLVDLVSKPKDV   456
3HBF_A|PDBID|CHAIN|SEQUENCE VDNGVLTKESIKKALELTMSSEKGGIMRQKIVKLKESAFKAVEQNGTSAMDFTTLIQIVTS----   454
                            .......410.......420.......430.......440.......450.......460.....
```

```
                            *:  ..        *:** ****.******* :.::::: :*.. ::** .  :* ::*      :  **  *::  .
Dianthus_2                  MTTTNNVEPPRR--HIAVCAFPFGTHAAPLLLLARRLSTATPDTRYTFFNVERSNTALFKAIDVSVGSNIVPYNITLSNE    78
2C1Z_A|PDBID|CHAIN|SEQUENCE MSQTTTNP------HVAVLAFPFSTHAAPLLAVVRRLAAAAPHAVFSFFSTSQSNASIFHDSMHTMQCNIKSYDISDGVP    74
3HBF_A|PDBID|CHAIN|SEQUENCE MSTFKNEMNGNNLLHVAVLAFPFGTHAAPLLSLVKKIATEAPKVTFSFFCTTTTNDTLFSRSNEFLP-NIKYYNVHDGLP    79
                            1.......10........20........30........40........50........60........70........80

                               . .*.* : *  * .    .*:: :  . *..*  :: :::***:*: :*:* *    *:.:****. ** :*:* *
Dianthus_2                  APAAGPGNPMKAIEEFLEHSPGGFRRAMEEVEAHVGVKISIIISDAFLWYCSDIAEEKGLDWIALWTAGTASLSAHLYTD   158
2C1Z_A|PDBID|CHAIN|SEQUENCE EGYVFAGRPQEDIELFTRAAPESFRQGMVMAVAETGRPVSCLVADAFIWFAADMAAEMGVAWLPFWTAGPNSLSTHVYID   154
3HBF_A|PDBID|CHAIN|SEQUENCE KGYVSSGNPREPIFLFIKAMQENFKHVIDEAVAETGKNITCLVTDAFFWFGADLAEEMHAKWVPLWTAGPHSLLTHVYTD   159
                            ........90.......100.......110.......120.......130.......140.......150.......160

                             **   . .  :.  :  :**:.:::  **  *::  :::  *: ***:**  **:*..* :*  *  :   : *:*:
Dianthus_2                  AIRALASDNQGEEGKEDVLVSIPGLSNVRISDLPMGLVSGDLDAPFSQMLHKMGTMLPKAKVVTLNVFQELSPLVVNDLN   238
2C1Z_A|PDBID|CHAIN|SEQUENCE EIREKIGVSGIQGREDELLNFIPGMSKVRFRDLQEGIVFGNLNSLFSRMLHRMGQVLPKATAVFINSFEELDDSLTNDLK   234
3HBF_A|PDBID|CHAIN|SEQUENCE LIREKTGSKEVHDVKS--IDVLPGFPELKASDLPEGVIK-DIDVPFATMLHKMGLELPRANAVAINSFATIHPLIENELN   236
                            .......170.......180.......190.......200.......210.......220.......230.......240

                            **:*  * :*** *  *    .:  .**:**.::  .::.*****:*.**** *: **:::** . .**:**: .. : :
Dianthus_2                  SKLK-LLCVGPFPLTCPPQVYSDPDNCLDWLDQHGPKTIAYISFGTVATPPPHEIRALAKALEGSDIPFVWSMSDSVRAN   317
2C1Z_A|PDBID|CHAIN|SEQUENCE SKLKTYLNIGPFNLITPPPVVPNTTGCLQWLKERKPTSVVYISFGTVTTPPPAEVVALSEALEASRVPFIWSLRDKARVH   314
3HBF_A|PDBID|CHAIN|SEQUENCE SKFKLLLNVGPFNLTTPQRKVSDEHGCLEWLDQHENSSVVYISFGSVVTPPPHELTALAESLEECGFPFIWSFRGDPKEK   316
                            .......250.......260.......270.......280.......290.......300.......310.......320

                            **:.*:*: :     * :*.**** ::* * :.*.*:**.****: *.:  ***:*.**::*** **  :.*  *::*:
Dianthus_2                  LPESFIEKIQNEPKVGKIVSWAPQIKLLGHPSTGVFVTHCGWNSIMESISTGVPLICRPIIGDQELNQRIVEIELKFGIG   397
2C1Z_A|PDBID|CHAIN|SEQUENCE LPEGFLEKTRG---YGMVVPWAPQAEVLAHEAVGAFVTHCGWNSLWESVAGGVPLICRPFFGDQRLNGRMVEDVLEIGVR   391
3HBF_A|PDBID|CHAIN|SEQUENCE LPKGFLERTKT---KGKIVAWAPQVEILKHSSVGVFLTHSGWNSVLECIVGGVPMISRPFFGDQGLNTILTESVLEIGVG   393
                            .......330.......340.......350.......360.......370.......380.......390.......400

                            ::.* :*:..  ..::  ::.***  :*:::  *:: * .:*   *:*  :* :*:::::.
Dianthus_2                  IEGGCFTQSGTTNALNQVLASEKGEEMRKNVEELKKLAEESVKQGGSSNENFNALIELITSS---   459
2C1Z_A|PDBID|CHAIN|SEQUENCE IEGGVFTKSGLMSCFDQILSQEKGKKLRENLRALRETADRAVGPKGSSTENFITLVDLVSKPKDV   456
3HBF_A|PDBID|CHAIN|SEQUENCE VDNGVLTKESIKKALELTMSSEKGGIMRQKIVKLKESAFKAVEQNGTSAMDFTTLIQIVTS----   454
                            .......410.......420.......430.......440.......450.......460.....
```

```
                                    : .   *********.:*.. *  :*  :*: **...***: *: :* ::*  .      *:* :.: **:*
Diospyros                   ------MADSSPHQHVAVLAFPFSSHPGLLRRIVCWLAAAAPDVTFSFYTTAESNKSLFSAPAP---ANVKAFHVSDGVP    71
2C1Z_A|PDBID|CHAIN|SEQUENCE ------MSQTTTNPHVAVLAFPFSTHAAPLLAVVRRLAAAAPHAVFSFFSTSQSNASIFHDSMHTMQCNIKSYDISDGVP    74
3HBF_A|PDBID|CHAIN|SEQUENCE MSTFKNEMNGNNLLHVAVLAFPFGTHAAPLLSLVKKIATEAPKVTFSFFCTTTTNDTLFSRSNE-FLPNIKYYNVHDGLP    79
                            1.......10........20........30........40........50........60........70........80

                            :*** :*.*:* * ** .*  .. :: :  * ** *: ::**::***:**..*:* ** . *:*.**:*  *: .*.* *
Diospyros                   EGYVFSGKPQEDINLFLEAGYRSCKKAVKAAEAEIGRRISCLMADAFLWFSGDLAEEMGVAWIPVWTSGVCSFSVHFYTD    151
2C1Z_A|PDBID|CHAIN|SEQUENCE EGYVFAGRPQEDIELFTRAAPESFRQGMVMAVAETGRPVSCLVADAFIWFAADMAAEMGVAWLPFWTAGPNSLSTHVYID    154
3HBF_A|PDBID|CHAIN|SEQUENCE KGYVSSGNPREPIFLFIKAMQENFKHVIDEAVAETGKNITCLVTDAFFWFGADLAEEMHAKWVPLWTAGPHSLLTHVYTD    159
                            ........90.......100.......110.......120.......130.......140.......150.......160

                             **:. * . : . :.  :..:**:.  ::  ** .*::  :::  *: ***:**  * :*. : :***  :.    : *
Diospyros                   LIRDTVGINALAGREDEIVKFIPGFSGGVRLGDLPSGVLFGNLDSTFAVMLHKMGKVLQKAKFLAVNSFEGLESEASEVL    231
2C1Z_A|PDBID|CHAIN|SEQUENCE EIREKIGVSGIQGREDELLNFIPGMS-KVRFRDLQEGIVFGNLNSLFSRMLHRMGQVLPKATAVFINSFEELDDSLTNDL    233
3HBF_A|PDBID|CHAIN|SEQUENCE LIREKTGSKEVHDVKS--IDVLPGFP-ELKASDLPEGVIK-DIDVPFATMLHKMGLELPRANAVAINSFATIHPLIENEL    235
                            .......170.......180.......190.......200.......210.......220.......230.......240

                            :*::.  * :**:**  *   .      :  ***:**..:: :**.**.**:* * .* *:.**:::**   .**:**::
Diospyros                   NSNFTKLLYIGPLNLTCPAPATGPSSFSDEYGCLKWLDSQKPTSVAYIGFGTVGTLAPDELVALANALEARAMPFLWSIK    311
2C1Z_A|PDBID|CHAIN|SEQUENCE KSKLKTYLNIGPFNLITPPPVVP-----NTTGCLQWLKERKPTSVVYISFGTVTTPPPAEVVALSEALEASRVPFIWSLR    308
3HBF_A|PDBID|CHAIN|SEQUENCE NSKFKLLLNVGPFNLTTPQRKVS-----DEHGCLEWLDQHENSSVVYISFGSVVTPPPHELTALAESLEECGFPFIWSFR    310
                            .......250.......260.......270.......280.......290.......300.......310.......320

                            .. : :**: :**: .  * :*.*:**.::* * :**.*:** ****: *.:..***:* **:***: **  :.* * .:
Diospyros                   DSLKKHLPEKLLEKMNRTGKMVPWTPQVQVLAHVSVGVFITHFGWNSVLESIAAGVPLIGRPLFGDHQLNGWMAEHVWRV    391
2C1Z_A|PDBID|CHAIN|SEQUENCE DKARVHLPEGFLEKTRGYGMVVPWAPQAEVLAHEAVGAFVTHCGWNSLWESVAGGVPLICRPFFGDQRLNGRMVEDVLEI    388
3HBF_A|PDBID|CHAIN|SEQUENCE GDPKEKLPKGFLERTKTKGKIVAWAPQVEILKHSSVGVFLTHSGWNSVLECIVGGVPMISRPFFGDQGLNTILTESVLEI    390
                            .......330.......340.......350.......360.......370.......380.......390.......400

                            ** ::.**:**..   .::  : . :*  ::::   ::* * :*.  :*:*: .* :*:. *
Diospyros                   GVKVEGGVFTKDGTIRALDLVLLEAEGKKLKENAEWFKEIAHKAAGKNGSSTRSFDSLVKSVRAQKDL    459
2C1Z_A|PDBID|CHAIN|SEQUENCE GVRIEGGVFTKSGLMSCFDQILSQEKGKKLRENLRALRETADRAVGPKGSSTENFITLVDLVSKPKDV    456
3HBF_A|PDBID|CHAIN|SEQUENCE GVGVDNGVLTKESIKKALELTMSSEKGGIMRQKIVKLKESAFKAVEQNGTSAMDFTTLIQIVTS----    454
                            .......410.......420.......430.......440.......450.......460........
```

```
                                .  :          ****:****.***. **.:::::*   *:. ****.*: :* ::*       :  *** ::: **:*
Eustoma                     ----KIQK------HVAVMAFPFATHAGLLLGLLHRLAGALPNVMFSFFSTAKSNASLFSG-KSPIQLNIKPFDIQDGVP     69
2C1Z_A|PDBID|CHAIN|SEQUENCE MSQTTTNP------HVAVLAFPFSTHAAPLLAVVRRLAAAAPHAVFSFFSTSQSNASIFHDSMHTMQCNIKSYDISDGVP     74
3HBF_A|PDBID|CHAIN|SEQUENCE MSTFKNEMNGNNLLHVAVLAFPFGTHAAPLLSLVKKIATEAPKVTFSFFCTTTTNDTLFSR-SNEFLPNIKYYNVHDGLP     79
                            1.......10........20........30........40........50........60........70........80

                            :*** :*.*:* * ** :*  *.*:: :  *   .*  ::*:::***:**..::* *:   *:*.**:*. ** .**: :
Eustoma                     EGYVFSGRPQEDIDLFLKAAPENFRKELKVAEEVVGIKVSCVMADAFLWFSGEMAEEVGGCWVPVWTSGAGSLSVHVHTE    149
2C1Z_A|PDBID|CHAIN|SEQUENCE EGYVFAGRPQEDIELFTRAAPESFRQGMVMAVAETGRPVSCLVADAFIWFAADMAAEMGVAWLPFWTAGPNSLSTHVYID    154
3HBF_A|PDBID|CHAIN|SEQUENCE KGYVSSGNPREPIFLFIKAMQENFKHVIDEAVAETGKNITCLVTDAFFWFGADLAEEMHAKWVPLWTAGPHSLLTHVYTD    159
                            ........90.......100.......110.......120.......130.......140.......150.......160

                             ***. *  . :  . :.  :..:**:.::: ** .*::  :::  *: ***:**  *.:*.*: ****  :.  : ::*:
Eustoma                     LIRETIGLNGVAGREDEILKFIPGFSELRLGDLPSGVVFGNLESPFAVMLHKMGQTLSKASALPINSFEELDPPLIQDLK    229
2C1Z_A|PDBID|CHAIN|SEQUENCE EIREKIGVSGIQGREDELLNFIPGMSKVRFRDLQEGIVFGNLNSLFSRMLHRMGQVLPKATAVFINSFEELDDSLTNDLK    234
3HBF_A|PDBID|CHAIN|SEQUENCE LIREKTGSKEVHDVKS--IDVLPGFPELKASDLPEGVIK-DIDVPFATMLHKMGLELPRANAVAINSFATIHPLIENELN    236
                            .......170.......180.......190.......200.......210.......220.......230.......240

                            **:*  **:***** :*    .        **: **.::.  *****.**:*.**** *: **:*:*     **:**::.. :
Eustoma                     SKFKNVLNVGPFNLTSPPPIANTTDIHGCIPWLDKQSPRSVVYIGFGTVATPPPPELKALAEALVDTKTPFLWSLKDNMR    309
2C1Z_A|PDBID|CHAIN|SEQUENCE SKLKTYLNIGPFNLITPPPVVPNT--TGCLQWLKERKPTSVVYISFGTVTTPPPAEVVALSEALEASRVPFIWSLRDKAR    312
3HBF_A|PDBID|CHAIN|SEQUENCE SKFKLLLNVGPFNLTTPQRKVSDE--HGCLEWLDQHENSSVVYISFGSVVTPPPHELTALAESLEECGFPFIWSFRGDPK    314
                            .......250.......260.......270.......280.......290.......300.......310.......320

                             :**:** *::   * :*.****.::* *.:**.*:.*.****: *.:..***:*.******: **   :.*.* :**: :
Eustoma                     SHLPEGFSERSGEIGKIVPWAPQVKILEHDSVGVFINHCGWNSVMESIAAGVPIICRPFFGDHQLNSWMVEKVWQIGLRI    389
2C1Z_A|PDBID|CHAIN|SEQUENCE VHLPEGFLEKTRGYGMVVPWAPQAEVLAHEAVGAFVTHCGWNSLWESVAGGVPLICRPFFGDQRLNGRMVEDVLEIGVRI    392
3HBF_A|PDBID|CHAIN|SEQUENCE EKLPKGFLERTKTKGKIVAWAPQVEILKHSSVGVFLTHSGWNSVLECIVGGVPMISRPFFGDQGLNTILTESVLEIGVGV    394
                            .......330.......340.......350.......360.......370.......380.......390.......400

                            :.**:**..   .::  :* ***  :::::   :: * .**  :*:*: .* :::::::.
Eustoma                     EGGVFTKDATNRALEVVFSHEKGKKIKEQVVAYKKLALEAVGPNGSSTKGFQAMVEVISTCQ--   451
2C1Z_A|PDBID|CHAIN|SEQUENCE EGGVFTKSGLMSCFDQILSQEKGKKLRENLRALRETADRAVGPKGSSTENFITLVDLVSKPKDV   456
3HBF_A|PDBID|CHAIN|SEQUENCE DNGVLTKESIKKALELTMSSEKGGIMRQKIVKLKESAFKAVEQNGTSAMDFTTLIQIVTS----   454
                            .......410.......420.......430.......440.......450.......460....
```

```
                                      :   *:.******.*******::*:::.  :.    .**** *: :* ::*         *** *:: **
Forsythia_intermedia         -------MAIH--SHIGVLAFPFGTHAAPLLTLVRRLVLDSSSQGITFSFFNTAKSNCAIFSG---QEFDNIKAYDVWDG    68
2C1Z_A|PDBID|CHAIN|SEQUENCE  ----MSQTTTN--PHVAVLAFPFSTHAAPLLAVVRRLAAAAP--HAVFSFFSTSQSNASIFHDSMHTMQCNIKSYDISDG    72
3HBF_A|PDBID|CHAIN|SEQUENCE  MSTFKNEMNGNNLLHVAVLAFPFGTHAAPLLSLVKKIATEAP--KVTFSFFCTTTTNDTLFSRS-NEFLPNIKYYNVHDG    77
                             1.......10........20........30........40........50........60........70........80

                               :* . :*    * : **  *   .*.: :  * .:.*  ::**::***:** .*:* *    *:.:***.. ** :*:
Forsythia_intermedia         THEGEAFTGSNILEAMQLFLAATPGNFEKVMKEAEVKNGMKISCLLSDAFLWFTCDLAEERGIPWVSFWTAASCSLSAHM   148
2C1Z_A|PDBID|CHAIN|SEQUENCE  VPEGYVFAGR-PQEDIELFTRAAPESFRQGMVMAVAETGRPVSCLVADAFIWFAADMAAEMGVAWLPFWTAGPNSLSTHV   151
3HBF_A|PDBID|CHAIN|SEQUENCE  LPKGYVSSGN-PREPIFLFIKAMQENFKHVIDEAVAETGKNITCLVTDAFFWFGADLAEEMHAKWVPLWTAGPHSLLTHV   156
                             ........90.......100.......110.......120.......130.......140.......150.......160

                             * * * .    .     : : :..:**:..::  ** * :: . ::  :: *:::*   *.::.*: :***  :.  : *
Forsythia_intermedia         YTDQIWSLMRSTGTAKTEEKTLSFVPGMTSVRFSDLPEEILSDNLESPLTLMIYKMVQKLSKSTAIVVNSFEEIDPVITN   228
2C1Z_A|PDBID|CHAIN|SEQUENCE  YIDEIREKIGVSGIQGREDELLNFIPGMSKVRFRDLQEGIVFGNLNSLFSRMLHRMGQVLPKATAVFINSFEELDDSLTN   231
3HBF_A|PDBID|CHAIN|SEQUENCE  YTDLIREKTGSK--EVHDVKSIDVLPGFPELKASDLPEGVIKD-IDVPFATMLHKMGLELPRANAVAINSFATIHPLIEN   233
                             .......170.......180.......190.......200.......210.......220.......230.......240

                             :*:**::  **:**  : :.     .  *  ** **.::. :**:*****:* ** * *:..*:*:**   .**:**:*.
Forsythia_intermedia         DLKSKFQNFLNIGPS-ILSSPTLSNGDSGQECLLWLEKQRHASVIYISFGTVITPQPREMAGLAEALETGEFPFLWSLRD   307
2C1Z_A|PDBID|CHAIN|SEQUENCE  DLKSKLKTYLNIGPFNLITPPPVVPNTTG--CLQWLKERKPTSVVYISFGTVTTPPPAEVVALSEALEASRVPFIWSLRD   309
3HBF_A|PDBID|CHAIN|SEQUENCE  ELNSKFKLLLNVGPFNLTTPQRKVSDEHG--CLEWLDQHENSSVVYISFGSVVTPPPHELTALAESLEECGFPFIWSFRG   311
                             .......250.......260.......270.......280.......290.......300.......310.......320

                             ..   **.***::*   * :*.**** ::* : :**.*:**.****: *.:  ***:*.******* **  :.*.* :**
Forsythia_intermedia         NAMKLLPDGFLDRTSKFGMIVSWAPQLKVLENPSVGAFITHCGWNSILESISFGVPMICRPFFGDQNLNSKMVEDVWKIG   387
2C1Z_A|PDBID|CHAIN|SEQUENCE  KARVHLPEGFLEKTRGYGMVVPWAPQAEVLAHEAVGAFVTHCGWNSLWESVAGGVPLICRPFFGDQRLNGRMVEDVLEIG   389
3HBF_A|PDBID|CHAIN|SEQUENCE  DPKEKLPKGFLERTKTKGKIVAWAPQVEILKHSSVGVFLTHSGWNSVLECIVGGVPMISRPFFGDQGLNTILTESVLEIG   391
                             .......330.......340.......350.......360.......370.......380.......390.......400

                             * ::.**:**..  ..:.  : .*.*  :*:::  *:..* .**  .*:*: :* :*::::..
Forsythia_intermedia         VRLEGGVFTKNGTIEALHSVMLNETGKAIRENINKLKRKAQNAVKFDGTSTKNFRALLELIKSPRGI   454
2C1Z_A|PDBID|CHAIN|SEQUENCE  VRIEGGVFTKSGLMSCFDQILSQEKGKKLRENLRALRETADRAVGPKGSSTENFITLVDLVSKPKDV   456
3HBF_A|PDBID|CHAIN|SEQUENCE  VGVDNGVLTKESIKKALELTMSSEKGGIMRQKIVKLKESAFKAVEQNGTSAMDFTTLIQIVTS----   454
                             .......410.......420.......430.......440.......450.......460.......
```

```
                            *:  ..  .     *********.******* :* ::*: ** . **** *. :* :::  .   :  . :   :: **:
Fragaria                    MA--SNQAGG----HVAVLAFPFSTHAAPLLNIVCRLAAAAPSTLFSFFNTKQSNSSILASDTSVLRYTNVCVCEVADGV   74
2C1Z_A|PDBID|CHAIN|SEQUENCE MS--QTTTNP----HVAVLAFPFSTHAAPLLAVVRRLAAAAPHAVFSFFSTSQSNASIFHDSMHTMQCN-IKSYDISDGV   73
3HBF_A|PDBID|CHAIN|SEQUENCE MSTFKNEMNGNNLLHVAVLAFPFGTHAAPLLSLVKKIATEAPKVTFSFFCTTTTNDTLFSRS-NEFLPN-IKYYNVHDGL   78
                            1.......10........20........30........40........50........60........70........80

                            *:***  *.*:* * ** :*  :.*:: :  :***:*: ::***:***:**.*.:* :*  . *:*:***** ** :**:
Fragaria                    PEGYVFVGKPQEDIELFMKAAPDNFRKCLEASVAESGREVSCLVTDAFFWFGAHMADDMGGVPWVPFWTAGPASLSAHVH  154
2C1Z_A|PDBID|CHAIN|SEQUENCE PEGYVFAGRPQEDIELFTRAAPESFRQGMVMAVAETGRPVSCLVADAFIWFAADMAAEMG-VAWLPFWTAGPNSLSTHVY  152
3HBF_A|PDBID|CHAIN|SEQUENCE PKGYVSSGNPREPIFLFIKAMQENFKHVIDEAVAETGKNITCLVTDAFFWFGADLAEEMH-AKWVPLWTAGPHSLLTHVY  157
                            ........90.......100.......110.......120.......130.......140.......150.......160

                             * **:.   ..    : : : .:.*:.:::  ** **::  :::  *: ***:**  ** *.** ****  :.  : *:
Fragaria                    TDLIRNTT--SGDCHDEKETITVIAGMSKVRPQDLPEGIIFGNLESLFSRMLHQMGLMLPLATAVFINSFEELDPVITND  232
2C1Z_A|PDBID|CHAIN|SEQUENCE IDEIREKIGVSGIQGREDELLNFIPGMSKVRFRDLQEGIVFGNLNSLFSRMLHRMGQVLPKATAVFINSFEELDDSLTND  232
3HBF_A|PDBID|CHAIN|SEQUENCE TDLIREKT--GSKEVHDVKSIDVLPGFPELKASDLPEGVIK-DIDVPFATMLHKMGLELPRANAVAINSFATIHPLIENE  234
                            .......170.......180.......190.......200.......210.......220.......230.......240

                            *:**:*  **:**::*  *   ..             ***.**.::: :****:***:*. *.* *: **:*:** . .*
Fragaria                    LKSKFKRFLNVGPLDLLEPTASAATTTPQTAEAVAGDGCLSWLDKQKAASVVYVSFGSVTRPSPEELMALAEALEASRVP  312
2C1Z_A|PDBID|CHAIN|SEQUENCE LKSKLKTYLNIGPFNLITPPPVVPNTT----------GCLQWLKERKPTSVVYISFGTVTTPPPAEVVALSEALEASRVP  302
3HBF_A|PDBID|CHAIN|SEQUENCE LNSKFKLLLNVGPFNLTTPQRKVSDEH----------GCLEWLDQHENSSVVYISFGSVVTPPPHELTALAESLEECGFP  304
                            .......250.......260.......270.......280.......290.......300.......310.......320

                            *:**:*.. :    . **.: :  * :*.**** ::* * :**.*:**.****: *.:.****:*.******* **  :.
Fragaria                    FLWSLRDNLKNPQLDEFLSKGKLNGMVVPWAPQPQVLAHGSVGAFVTHCGWNSVLESVAGGVPLICRPFFGDQKLNARMV  392
2C1Z_A|PDBID|CHAIN|SEQUENCE FIWSLRDKARVHLPEGFLEKTRGYGMVVPWAPQAEVLAHEAVGAFVTHCGWNSLWESVAGGVPLICRPFFGDQRLNGRMV  382
3HBF_A|PDBID|CHAIN|SEQUENCE FIWSFRGDPKEKLPKGFLERTKTKGKIVAWAPQVEILKHSSVGVFLTHSGWNSVLECIVGGVPMISRPFFGDQGLNTILT  384
                            .......330.......340.......350.......360.......370.......380.......390.......400

                            *.* :**: ::.**:**..: ..::  :*.:**  :::::  *:: * :**  :*:*: :* :*:::.:.
Fragaria                    EDVWKIGLRLEGGVFTKNGMLKSLDMLLSQDKGTKMKNKIHTLKQLAQQAVEPKGSSTRNFESLLEMATTN---   463
2C1Z_A|PDBID|CHAIN|SEQUENCE EDVLEIGVRIEGGVFTKSGLMSCFDQILSQEKGKKLRENLRALRETADRAVGPKGSSTENFITLVDLVSKPKDV   456
3HBF_A|PDBID|CHAIN|SEQUENCE ESVLEIGVGVDNGVLTKESIKKALELTMSSEKGGIMRQKIVKLKESAFKAVEQNGTSAMDFTTLIQIVTS----   454
                            .......410.......420.......430.......440.......450.......460.......470....
```

```
                                 .: . :   *********.*******::.:::*: :*  . **** *. :*  ::  . . :  *:* *:: ** *
Garcinia_mangostana          ----MTKPTTDDHPHVAVLAFPFGTHAAPLLSITHHLAALSPSTHFSFFGTPSSNSFILSSNTN-LPPNVKPYDVWDGTP    75
2C1Z_A|PDBID|CHAIN|SEQUENCE  ----MSQTTTN--PHVAVLAFPFSTHAAPLLAVVRRLAAAAPHAVFSFFSTSQSNASIFHDSMHTMQCNIKSYDISDGVP    74
3HBF_A|PDBID|CHAIN|SEQUENCE  MSTFKNEMNGNNLLHVAVLAFPFGTHAAPLLSLVKKIATEAPKVTFSFFCTTTTNDTLFSRSNE-FLPNIKYYNVHDGLP    79
                             1.......10........20........30........40........50........60........70........80

                             .**. :*  :* : **  *  *.*:: :  ** *:*: ::**::***:**. ::* *:   . *:*:*****::* :*:*
Garcinia_mangostana          DGYAYTGDVQEEMGLFISAAHESFRKGVDRAVEESGRRVSCLMSDAFFWFGKEMAEEIGGGVMWVPFWTAGPHALSSHLY    155
2C1Z_A|PDBID|CHAIN|SEQUENCE  EGYVFAGRPQEDIELFTRAAPESFRQGMVMAVAETGRPVSCLVADAFIWFAADMAAEMG--VAWLPFWTAGPNSLSTHVY    152
3HBF_A|PDBID|CHAIN|SEQUENCE  KGYVSSGNPREPIFLFIKAMQENFKHVIDEAVAETGKNITCLVTDAFFWFGADLAEEMH--AKWVPLWTAGPHSLLTHVY    157
                             ........90.......100.......110.......120.......130.......140.......150.......160

                              * ***. . .  :  : : :. :**:..::  ** **::   ::  *: ***:**  **:* ** ****  :.  : *:
Garcinia_mangostana          TDFIRESFAGDVTQ-REDELLSSIPGMSRVRVCDLPEGVVFGRLDSLFSQMLHKMGQALPKADAVFINSFEELDPTFTND    234
2C1Z_A|PDBID|CHAIN|SEQUENCE  IDEIREKIGVSGIQGREDELLNFIPGMSKVRFRDLQEGIVFGNLNSLFSRMLHRMGQVLPKATAVFINSFEELDDSLTND    232
3HBF_A|PDBID|CHAIN|SEQUENCE  TDLIREKTGSKEVH--DVKSIDVLPGFPELKASDLPEGVIK-DIDVPFATMLHKMGLELPRANAVAINSFATIHPLIENE    234
                             .......170.......180.......190.......200.......210.......220.......230.......240

                             *:**:*  **:***** :*   *.:  **: **.::: :**.*:***:.. * * *:.**:*:**   .*****:....:
Garcinia_mangostana          LKSKLKCCLNIGPFNLISPPAQVPDTYGCIPWLDKQQLASVAYVSFGSATIPLPHELVALAEALEDRKVPFIWSLKDNAK    314
2C1Z_A|PDBID|CHAIN|SEQUENCE  LKSKLKTYLNIGPFNLITPPPVVPNTTGCLQWLKERKPTSVVYISFGTVTTPPPAEVVALSEALEASRVPFIWSLRDKAR    312
3HBF_A|PDBID|CHAIN|SEQUENCE  LNSKFKLLLNVGPFNLTTPQRKVSDEHGCLEWLDQHENSSVVYISFGSVVTPPPHELTALAESLEECGFPFIWSFRGDPK    314
                             .......250.......260.......270.......280.......290.......300.......310.......320

                              :**.**** *:  * ::.****.::* *.:**.*:**.****: * :.****:*.***:*** **  :  .* :*** :
Garcinia_mangostana          VHLPDGFLETTKFQGIVIPWAPQAKVLGHKAVGVFITHCGWNSLLETIVGGVPVICRPFYGDQRLNARMIGDVWKIGVIV    394
2C1Z_A|PDBID|CHAIN|SEQUENCE  VHLPEGFLEKTRGYGMVVPWAPQAEVLAHEAVGAFVTHCGWNSLWESVAGGVPLICRPFFGDQRLNGRMVEDVLEIGVRI    392
3HBF_A|PDBID|CHAIN|SEQUENCE  EKLPKGFLERTKTKGKIVAWAPQVEILKHSSVGVFLTHSGWNSVLECIVGGVPMISRPFFGDQGLNTILTESVLEIGVGV    394
                             .......330.......340.......350.......360.......370.......380.......390.......400

                             :.**::*..: ..:: : .*.*  :* .: *:: *  *. :*:*: ::  * :*:.
Garcinia_mangostana          NGGVLAKEAMIDCFDKILLQEDGKQMRGRIKSLKDLALAATAYKGSSSDNMRELSRLVSSPCK-    457
2C1Z_A|PDBID|CHAIN|SEQUENCE  EGGVFTKSGLMSCFDQILSQEKGKKLRENLRALRETADRAVGPKGSSTENFITLVDLVSKPKDV    456
3HBF_A|PDBID|CHAIN|SEQUENCE  DNGVLTKESIKKALELTMSSEKGGIMRQKIVKLKESAFKAVEQNGTSAMDFTTLIQIVTS----    454
                             .......410.......420.......430.......440.......450.......460....
```

```
                            **  .         *********.*******::*.::*: **.  ****.*: :  ::*  :    :  *** * : **
Gentiana_triflora           MSPVS---------HVAVLAFPFGTHAAPLLTLVNRLAASAPDIIFSFFSTSSSITTIFSPTNLISIGSNIKPYAVWDGS    71
2C1Z_A|PDBID|CHAIN|SEQUENCE MSQTTTNP------HVAVLAFPFSTHAAPLLAVVRRLAAAAPHAVFSFFSTSQSNASIFHDS-MHTMQCNIKSYDISDGV    73
3HBF_A|PDBID|CHAIN|SEQUENCE MSTFKNEMNGNNLLHVAVLAFPFGTHAAPLLSLVKKIATEAPKVTFSFFCTTTTNDTLFSRS--NEFLPNIKYYNVHDGL    78
                            1.......10........20........30........40........50........60........70........80

                            *:*:* :*.*:* *  * .*  :.* : :  ** :**  ::**::***:**.**:: :: . *:*.***.. **  ***
Gentiana_triflora           PEGFVFSGNPREPIEYFLNAAPDNFDKAMKKAVEDTGVNISCLLTDAFLWFAADFSEKIGVPWIPVWTAASCSLCLHVYT   151
2C1Z_A|PDBID|CHAIN|SEQUENCE PEGYVFAGRPQEDIELFTRAAPESFRQGMVMAVAETGRPVSCLVADAFIWFAADMAAEMGVAWLPFWTAGPNSLSTHVYI   153
3HBF_A|PDBID|CHAIN|SEQUENCE PKGYVSSGNPREPIFLFIKAMQENFKHVIDEAVAETGKNITCLVTDAFFWFGADLAEEMHAKWVPLWTAGPHSLLTHVYT   158
                            ........90.......100.......110.......120.......130.......140.......150.......160

                            * **.: .    :     : :::**:. :   ** * ::  : :  *:  **.**  * :*.** :***  :.  : *.*
Gentiana_triflora           DEIRSRFAEFDIAEKAEKTIDFIPGLSAISFSDLPEELIMEDSQSIFALTLHNMGLKLHKATAVAVNSFEEIDPIITNHL   231
2C1Z_A|PDBID|CHAIN|SEQUENCE DEIREKIGVSGIQGREDELLNFIPGMSKVRFRDLQEGIVFGNLNSLFSRMLHRMGQVLPKATAVFINSFEELDDSLTNDL   233
3HBF_A|PDBID|CHAIN|SEQUENCE DLIREKTGSKEVHD--VKSIDVLPGFPELKASDLPEGVIK-DIDVPFATMLHKMGLELPRANAVAINSFATIHPLIENEL   235
                            .......170.......180.......190.......200.......210.......220.......230.......240

                            .*. :   **:**::  :.    .:   **:**. :: :****:***:* .*** *:.**:.:**   .**:**:*...:
Gentiana_triflora           RSTNQLNILNIGPLQTLSSS-IPPEDNECLKWLQTQKESSVVYLSFGTVINPPPNEMAALASTLESRKIPFLWSLRDEAR   310
2C1Z_A|PDBID|CHAIN|SEQUENCE KSKLKT-YLNIGPFNLITPPPVVPNTTGCLQWLKERKPTSVVYISFGTVTTPPPAEVVALSEALEASRVPFIWSLRDKAR   312
3HBF_A|PDBID|CHAIN|SEQUENCE NSKFKL-LLNVGPFNLTTPQRKVSDEHGCLEWLDQHENSSVVYISFGSVVTPPPHELTALAESLEECGFPFIWSFRGDPK   314
                            .......250.......260.......270.......280.......290.......300.......310.......320

                             :**:.*:::*    * :*.**** .:* : ::*.*:**.****  *.:   **:* ******* :*  :.*.* :*** :
Gentiana_triflora           KHLPENFIDRTSTFGKIVSWAPQLHVLENPAIGVFVTHCGWNSTLESIFCRVPVIGRPFFGDQKVNARMVEDVWKIGVGV   390
2C1Z_A|PDBID|CHAIN|SEQUENCE VHLPEGFLEKTRGYGMVVPWAPQAEVLAHEAVGAFVTHCGWNSLWESVAGGVPLICRPFFGDQRLNGRMVEDVLEIGVRI   392
3HBF_A|PDBID|CHAIN|SEQUENCE EKLPKGFLERTKTKGKIVAWAPQVEILKHSSVGVFLTHSGWNSVLECIVGGVPMISRPFFGDQGLNTILTESVLEIGVGV   394
                            .......330.......340.......350.......360.......370.......380.......390.......400

                            ..**:*:.     ::  : .:**  :*:::  *:*.*  **  :*:*: :* :*:  ...
Gentiana_triflora           KGGVFTEDETTRVLELVLFSDKGKEMRQNVGRLKEKAKDAVKANGSSTRNFESLLAAFNKLDS-   453
2C1Z_A|PDBID|CHAIN|SEQUENCE EGGVFTKSGLMSCFDQILSQEKGKKLRENLRALRETADRAVGPKGSSTENFITLVDLVSKPKDV   456
3HBF_A|PDBID|CHAIN|SEQUENCE DNGVLTKESIKKALELTMSSEKGGIMRQKIVKLKESAFKAVEQNGTSAMDFTTLIQIVTS----   454
                            .......410.......420.......430.......440.......450.......460....
```

```
                                     .         *:*:*****.*******:.:.: :*: **   .*  * ::   . : :  *       **: *:: ** *      :.       *.* : ** :     .::: :  **    :*  ::*:::***:** .::* *   . *:*
Iris_2                      -MGSVEHP------HIALLAFPFGTHAAPLLSLAHSLAASAPPGTFFSFVSSRRSVSSLSLSVSPSD-NIRFYEVSDGSPVLAPAAASGMLEDPEEEVRLFMKETPGNYRRALEAAVEGCAGTRVTCIIADAFLWFVGEIAAENGVGWVP   142
2C1Z_A|PDBID|CHAIN|SEQUENCE MSQTTTNP------HVAVLAFPFSTHAAPLLAVVRRLAAAAPHAVFSFFSTSQSNASIFHDSMHTMQCNIKSYDISDGVPEGYVFAGR-----PQEDIELFTRAAPESFRQGMVMAVA-ETGRPVSCLVADAFIWFAADMAAEMGVAWLP   138
3HBF_A|PDBID|CHAIN|SEQUENCE MSTFKNEMNGNNLLHVAVLAFPFGTHAAPLLSLVKKIATEAPKVTFSFFCTTTTNDTLFSRSNEFLP-NIKYYNVHDGLPKGYVSSGN-----PREPIFLFIKAMQENFKHVIDEAVA-ETGKNITCLVTDAFFWFGADLAEEMHAKWVP   143
                            1.......10........20........30........40........50........60........70........80........90.......100.......110.......120.......130.......140.......150

                            :**.** *: :*:* * :*:: *      . .   ::.:**:..::  ** *.::  .::  *: **::*.  **::. :: :***  :.  :  :* :*::  * :**:**  *   *.:        ** **.: :  :***:***:*.
Iris_2                      LWTGGPCSFQAHLYTDLLRDRIGVGEKADLDAD-LQFIPGLASLRVRDLPEDIVTGHLDGAFATMLYRMATELPRSTSTIILNSFEGLHPEIDADLATKFRKPLPIGPLNLLFPSPAVPEPVSSSRCLAWLDKFEPDTVVYVSFGTVVDL   291
2C1Z_A|PDBID|CHAIN|SEQUENCE FWTAGPNSLSTHVYIDEIREKIGVSGIQGREDELLNFIPGMSKVRFRDLQEGIVFGNLNSLFSRMLHRMGQVLPKAT-AVFINSFEELDDSLTNDLKSKLKTYLNIGPFNLITPPPVVPN---TTGCLQWLKERKPTSVVYISFGTVTTP   284
3HBF_A|PDBID|CHAIN|SEQUENCE LWTAGPHSLLTHVYTDLIREKTGSKEVHDVKS--IDVLPGFPELKASDLPEGVIK-DIDVPFATMLHKMGLELPRAN-AVAINSFATIHPLIENELNSKFKLLLNVGPFNLTTPQRKVSD---EHGCLEWLDQHENSSVVYISFGSVVTP   286
                            .......160.......170.......180.......190.......200.......210.......220.......230.......240.......250.......260.......270.......280.......290.......300

                            ** *:. *: .** .  **:**::. .: :** ***::*:  * :*.* **. :* *.:*..*::*.****: *.:. ***::.***:*** **   :...* ::** :..* :*. .: ..:.  :: ::*  :*:.    ::*.*  :*
Iris_2                      PPSELAELALGLESSGSPFLWSIKDPAKAKLPAGFLDRTRDRGLLVPWIPQVAVLNHNAVAAFLSHCGWNSVLESMTCGVPMVCRPFLGDQMLNSKVVSQVWKVGVRLHNGPMTSTNVAEAIKTVVAGDEGKNMRDRAAKMREKATGSVR   441
2C1Z_A|PDBID|CHAIN|SEQUENCE PPAEVVALSEALEASRVPFIWSLRDKARVHLPEGFLEKTRGYGMVVPWAPQAEVLAHEAVGAFVTHCGWNSLWESVAGGVPLICRPFFGDQRLNGRMVEDVLEIGVRIEGGVFTKSGLMSCFDQILSQEKGKKLRENLRALRETADRAVG   434
3HBF_A|PDBID|CHAIN|SEQUENCE PPHELTALAESLEECGFPFIWSFRGDPKEKLPKGFLERTKTKGKIVAWAPQVEILKHSSVGVFLTHSGWNSVLECIVGGVPMISRPFFGDQGLNTILTESVLEIGVGVDNGVLTKESIKKALELTMSSEKGGIMRQKIVKLKESAFKAVE   436
                            .......310.......320.......330.......340.......350.......360.......370.......380.......390.......400.......410.......420.......430.......440.......450

                             .*:*. :: **:::*
Iris_2                      PDGSSVRNLNTLLEIVFAR---   460
2C1Z_A|PDBID|CHAIN|SEQUENCE PKGSSTENFITLVDLVSKPKDV   456
3HBF_A|PDBID|CHAIN|SEQUENCE QNGTSAMDFTTLIQIVTS----   454
                            .......460.......470..
```

```
                                      :    .   *********.*******::: ::*: **:. ****.*: :* :::     . :  *:* *:: :*:*
Litchi                             ----MHQPDNASDPHVAVLAFPFSTHAAPLLSIISRLASSAPNTHFSFFSTAESNNSLLSTHKHYFLPNVKAYNVSNGVP    76
2C1Z_A|PDBID|CHAIN|SEQUENCE        ----MSQT--TTNPHVAVLAFPFSTHAAPLLAVVRRLAAAAPHAVFSFFSTSQSNASIFHDSMHTMQCNIKSYDISDGVP    74
3HBF_A|PDBID|CHAIN|SEQUENCE        MSTFKNEMNGNNLLHVAVLAFPFGTHAAPLLSLVKKIATEAPKVTFSFFCTTTTNDTLFSRSNE-FLPNIKYYNVHDGLP    79
                                   1.......10........20........30........40........50........60........70........80

                                   ..**  *.*:* * ** :*  *.::: :  *..** : ::***:*:*:**.*::* **  . ::* * :*.  ** **.* *
Litchi                             DNYVLLGKPQEDIELFMKAAPENLRKAVAKAAVETRRKVSCLVTDSFLWFAAEMAEEMQVPFVPCWLSGSSSLSTHFYTD   156
2C1Z_A|PDBID|CHAIN|SEQUENCE        EGYVFAGRPQEDIELFTRAAPESFRQGMVMAVAETGRPVSCLVADAFIWFAADMAAEMGVAWLPFWTAGPNSLSTHVYID   154
3HBF_A|PDBID|CHAIN|SEQUENCE        KGYVSSGNPREPIFLFIKAMQENFKHVIDEAVAETGKNITCLVTDAFFWFGADLAEEMHAKWVPLWTAGPHSLLTHVYTD   159
                                   ........90.......100.......110.......120.......130.......140.......150.......160

                                    **** * . :.. :.  :..: *:.::   ** **::  :::  *: ***:**  **:. ** ****  :.  : *:*:
Litchi                             VIREKMGLEGIEGREDEQLKFIQGMSKVCIRDLPEGVLFGNLQSIFSDMLHRMGLQLPQGDAVVINSFEELDPTINNDLK   236
2C1Z_A|PDBID|CHAIN|SEQUENCE        EIREKIGVSGIQGREDELLNFIPGMSKVRFRDLQEGIVFGNLNSLFSRMLHRMGQVLPKATAVFINSFEELDDSLTNDLK   234
3HBF_A|PDBID|CHAIN|SEQUENCE        LIREKTGSKEVHDVKS--IDVLPGFPELKASDLPEGVIK-DIDVPFATMLHKMGLELPRANAVAINSFATIHPLIENELN   236
                                   .......170.......180.......190.......200.......210.......220.......230.......240

                                   **:*  **:***** :*   *.:  .** **..:: :**.*:.**:*   ** *:.*::*:** . .*****:: . : .
Litchi                             SKFKQFLNVGPFNLISPPPAVPDTSSCLPWLDRQKPASVAYLGFGSVSRLPPNEIVAVAEALEASKLPFIWSLKKNLQAD   316
2C1Z_A|PDBID|CHAIN|SEQUENCE        SKLKTYLNIGPFNLITPPPVVPNTTGCLQWLKERKPTSVVYISFGTVTTPPPAEVVALSEALEASRVPFIWSLRDKARVH   314
3HBF_A|PDBID|CHAIN|SEQUENCE        SKFKLLLNVGPFNLTTPQRKVSDEHGCLEWLDQHENSSVVYISFGSVVTPPPHELTALAESLEECGFPFIWSFRGDPKEK   316
                                   .......250.......260.......270.......280.......290.......300.......310.......320

                                   **:  *:      * :* **** ::* *.:**.*:.* **.*: *.:.  ***:* ******* **  :.:.  :*** ::.
Litchi                             LPNRKLN-----GIMVEWAPQLDVLAHNAVGVFINHGGWSSLMESMACGVPMIIRPFFGDQRLNARMVQDEWKIGVSVEG   391
2C1Z_A|PDBID|CHAIN|SEQUENCE        LPEGFLEKTRGYGMVVPWAPQAEVLAHEAVGAFVTHCGWNSLWESVAGGVPLICRPFFGDQRLNGRMVEDVLEIGVRIEG   394
3HBF_A|PDBID|CHAIN|SEQUENCE        LPKGFLERTKTKGKIVAWAPQVEILKHSSVGVFLTHSGWNSVLECIVGGVPMISRPFFGDQGLNTILTESVLEIGVGVDN   396
                                   .......330.......340.......350.......360.......370.......380.......390.......400

                                   *::** .:  .::  : .*:*  :*:::  ::: *  **  :*:*  :* :*:::*:
Litchi                             GIITKRGFLRSLDLILWQENGKKMRENVRKFKQLAETAVGPQGSSMKNFKALVDIVSRPNDA   453
2C1Z_A|PDBID|CHAIN|SEQUENCE        GVFTKSGLMSCFDQILSQEKGKKLRENLRALRETADRAVGPKGSSTENFITLVDLVSKPKDV   456
3HBF_A|PDBID|CHAIN|SEQUENCE        GVLTKESIKKALELTMSSEKGGIMRQKIVKLKESAFKAVEQNGTSAMDFTTLIQIVTS----   454
                                   .......410.......420.......430.......440.......450.......460..
```

```
                                 *.  .          ****:***:.:*.. **::*:::*: **  ..**** *: :* ::*          *** *:: **:*
Lobelia_erinus                   MPVVT-----APERHVAVFAFPIASHPGLLLSLVRRLATAAPTVTFSFFNTAKSNRSLFS---VPTPHNIKPYDVSDGIP    72
2C1Z_A|PDBID|CHAIN|SEQUENCE      MSQTT-----TNP-HVAVLAFPFSTHAAPLLAVVRRLAAAAPHAVFSFFSTSQSNASIFHDSMHTMQCNIKSYDISDGVP    74
3HBF_A|PDBID|CHAIN|SEQUENCE      MSTFKNEMNGNNLLHVAVLAFPFGTHAAPLLSLVKKIATEAPKVTFSFFCTTTTNDTLFSRS-NEFLPNIKYYNVHDGLP    79
                                 1.......10........20........30........40........50........60........70........80

                                  *** :*.*:* * **   .   :.*:: :   *  .***: ::***:***:**.:::* :   . *:*:**:*. **   *.* *
Lobelia_erinus                   VGYVFAGKPQEDINLFLAVAEDEFRRSLQVAEGETGKGISCLVADAFLWFSSEMAEKRRVPWVPLWTSGASSLSIHFYTD   152
2C1Z_A|PDBID|CHAIN|SEQUENCE      EGYVFAGRPQEDIELFTRAAPESFRQGMVMAVAETGRPVSCLVADAFIWFAADMAAEMGVAWLPFWTAGPNSLSTHVYID   154
3HBF_A|PDBID|CHAIN|SEQUENCE      KGYVSSGNPREPIFLFIKAMQENFKHVIDEAVAETGKNITCLVTDAFFWFGADLAEEMHAKWVPLWTAGPHSLLTHVYTD   159
                                 ........90.......100.......110.......120.......130.......140.......150.......160

                                  **:. * . :.. :.  :::.:**:. ::   **   *::   :::   *: **::**   *.:*.*: ****   :.   : ::*:
Lobelia_erinus                   LIRQTVGLNGIEGRKDEMLDFIPGFSAVRLGDLPGGVLSGNLESPFSIMLYKMGQCLTKATAIPINSFQEMDPEINKDLN   232
2C1Z_A|PDBID|CHAIN|SEQUENCE      EIREKIGVSGIQGREDELLNFIPGMSKVRFRDLQEGIVFGNLNSLFSRMLHRMGQVLPKATAVFINSFEELDDSLTNDLK   234
3HBF_A|PDBID|CHAIN|SEQUENCE      LIREKTGSKEVHDVKS--IDVLPGFPELKASDLPEGVIK-DIDVPFATMLHKMGLELPRANAVAINSFATIHPLIENELN   236
                                 .......170.......180.......190.......200.......210.......220.......230.......240

                                 .::.   **:***** :*      .:   .*: **.::    **.**.**:*.**** *:.**:*:**  .   **:**:.  ..:
Lobelia_erinus                   KHFSNFLNIGPFNLISPTTPLNTDEFACIPWLDKHLPQSVAYIGFGTVATPPPHELVALAEALEESGTPFLWSINENSKK   312
2C1Z_A|PDBID|CHAIN|SEQUENCE      SKLKTYLNIGPFNLITPP-PVVPNTTGCLQWLKERKPTSVVYISFGTVTTPPPAEVVALSEALEASRVPFIWSLRDKARV   313
3HBF_A|PDBID|CHAIN|SEQUENCE      SKFKLLLNVGPFNLTTPQ-RKVSDEHGCLEWLDQHENSSVVYISFGSVVTPPPHELTALAESLEECGFPFIWSFRGDPKE   315
                                 .......250.......260.......270.......280.......290.......300.......310.......320

                                 :**:****:*    * :*.****.::* * :**.*:** ****: *.:  .***:* *****:* **   :.*.* .*** :.
Lobelia_erinus                   HLPEGFLERTGENGKLVPWAPQVQVLAHGSVGVFITHGGWNSVVESIGAGVPLIMRPFFGEQQLNTWMVENVWRIGVRVK   392
2C1Z_A|PDBID|CHAIN|SEQUENCE      HLPEGFLEKTRGYGMVVPWAPQAEVLAHEAVGAFVTHCGWNSLWESVAGGVPLICRPFFGDQRLNGRMVEDVLEIGVRIE   393
3HBF_A|PDBID|CHAIN|SEQUENCE      KLPKGFLERTKTKGKIVAWAPQVEILKHSSVGVFLTHSGWNSVLECIVGGVPMISRPFFGDQGLNTILTESVLEIGVGVD   395
                                 .......330.......340.......350.......360.......370.......380.......390.......400

                                 .**:*:..   ..::   :   :**   :::::   ::* * :**   .*:*: :* **:::::.
Lobelia_erinus                   GGVFTRSGTVQALEEVLLHQKGKKLKEQVVVFKELAHKAVGPSGSSTENFKTLIEIITNMEQSAT   457
2C1Z_A|PDBID|CHAIN|SEQUENCE      GGVFTKSGLMSCFDQILSQEKGKKLRENLRALRETADRAVGPKGSSTENFITLVDLVSKPKDV--   456
3HBF_A|PDBID|CHAIN|SEQUENCE      NGVLTKESIKKALELTMSSEKGGIMRQKIVKLKESAFKAVEQNGTSAMDFTTLIQIVTS------   454
                                 .......410.......420.......430.......440.......450.......460.....
```

```
                                          :   *:.******.***.***::*:::*: :*  . ***: :: :* ::*        **: ::: ** *
Perilla_3                        -------MGFE--LHIGVLAFPFGTHAPPLLALVRRLAASSPGTLFSFLNSAESNAALFNERTYD---NIRAFDVWDGTP    68
2C1Z_A|PDBID|CHAIN|SEQUENCE      ----MSQTTTN--PHVAVLAFPFSTHAAPLLAVVRRLAAAAPHAVFSFFSTSQSNASIFHDSMHTMQCNIKSYDISDGVP    74
3HBF_A|PDBID|CHAIN|SEQUENCE      MSTFKNEMNGNNLLHVAVLAFPFGTHAAPLLSLVKKIATEAPKVTFSFFCTTTTNDTLFS-RSNEFLPNIKYYNVHDGLP    79
                                 1.......10........20........30........40........50........60........70........80

                                 :* : :*   * : ** :*   .* : :  * .:**  : **::***:**..*:* :    *:*:***.. ** :*:* *
Perilla_3                        EGRIFTGTHFEAVGLFLKASPGNFDKVIEEAEPKTGLKICCLITDAFLWFACDMAQKRGLPWVPFWTAASCSLSSHLYTD   148
2C1Z_A|PDBID|CHAIN|SEQUENCE      EGYVFAGRPQEDIELFTRAAPESFRQGMVMAVAETGRPVSCLVADAFIWFAADMAAEMGVAWLPFWTAGPNSLSTHVYID   154
3HBF_A|PDBID|CHAIN|SEQUENCE      KGYVSSGNPREPIFLFIKAMQENFKHVIDEAVAETGKNITCLVTDAFFWFGADLAEEMHAKWVPLWTAGPHSLLTHVYTD   159
                                 ........90.......100.......110.......120.......130.......140.......150.......160

                                  * :        .: : :..:**:      **   :. .     ::  :::*   **::.** :***  :.  : ::*:
Perilla_3                        QIVKAG-----TANQEQNLSFIPGLEMATLTDLPPEVFLDNSPSPLAITINKMVEKLPKSTAVVLNSFEEIDPIITDDLK   223
2C1Z_A|PDBID|CHAIN|SEQUENCE      EIREKIGVSGIQGREDELLNFIPGMSKVRFRDLQEGIVFGNLNSLFSRMLHRMGQVLPKATAVFINSFEELDDSLTNDLK   234
3HBF_A|PDBID|CHAIN|SEQUENCE      LIREKTGSK--EVHDVKSIDVLPGFPELKASDLPEGVIKD-IDVPFATMLHKMGLELPRANAVAINSFATIHPLIENELN   236
                                 .......170.......180.......190.......200.......210.......220.......230.......240

                                 :*:*  **:**  * :*    ..:   ***.** ::    .********:* ***  *:.**:::** . .**:**::. .
Perilla_3                        TKFKNFLNVGPSILASPPQATPDDETGCLSWLADQTSPKSVVYISFGTVITPPENELAALADALEICRFPFLWSLKDYAV   303
2C1Z_A|PDBID|CHAIN|SEQUENCE      SKLKTYLNIGPFNLITPPPVVPN-TTGCLQWLKERK-PTSVVYISFGTVTTPPPAEVVALSEALEASRVPFIWSLRDKAR   312
3HBF_A|PDBID|CHAIN|SEQUENCE      SKFKLLLNVGPFNLTTPQRKVSD-EHGCLEWLDQHE-NSSVVYISFGSVVTPPPHELTALAESLEECGFPFIWSFRGDPK   314
                                 .......250.......260.......270.......280.......290.......300.......310.......320

                                   **.***::*:  * :*.**** ::* *  **.*:**.****: *.: . **:*.******* **   :.:.  :*** :
Perilla_3                        KSLPDGFLDRTKGFGKIVAWAPQQQVLAHRNVGVFVTHCGWNSILESISSCVPLICRPFFGDQKLNSRMVQDSWKIGVRV   383
2C1Z_A|PDBID|CHAIN|SEQUENCE      VHLPEGFLEKTRGYGMVVPWAPQAEVLAHEAVGAFVTHCGWNSLWESVAGGVPLICRPFFGDQRLNGRMVEDVLEIGVRI   392
3HBF_A|PDBID|CHAIN|SEQUENCE      EKLPKGFLERTKTKGKIVAWAPQVEILKHSSVGVFLTHSGWNSVLECIVGGVPMISRPFFGDQGLNTILTESVLEIGVGV   394
                                 .......330.......340.......350.......360.......370.......380.......390.......400

                                 :.**:**.   ..:.  :: * *  :*:::  *:*.*  **  :*:*: :* .*::::
Perilla_3                        EGGVFTKNEAVESLKKLMATEAGMKIRENVSLLREKATAAVKPEGSSSQNFKKLLEIIGAAESS   447
2C1Z_A|PDBID|CHAIN|SEQUENCE      EGGVFTKSGLMSCFDQILSQEKGKKLRENLRALRETADRAVGPKGSSTENFITLVDLVSKPKDV   456
3HBF_A|PDBID|CHAIN|SEQUENCE      DNGVLTKESIKKALELTMSSEKGGIMRQKIVKLKESAFKAVEQNGTSAMDFTTLIQIVTS----   454
                                 .......410.......420.......430.......440.......450.......460....
```

```
                                      *   *********.***. **.:*:::*   *:..*:** *: :* ::*   . .   *** ::: **:*
Petunia_2                   --------MSN--YHVAVLAFPFATHAGLLLGLVQRLANALPNVTFTFFNTSKSNSSLFTTPHDN---NIKPFNISDGVP    67
2C1Z_A|PDBID|CHAIN|SEQUENCE ----MSQTTTN--PHVAVLAFPFSTHAAPLLAVVRRLAAAAPHAVFSFFSTSQSNASIFHDSMHTMQCNIKSYDISDGVP    74
3HBF_A|PDBID|CHAIN|SEQUENCE MSTFKNEMNGNNLLHVAVLAFPFGTHAAPLLSLVKKIATEAPKVTFSFFCTTTTNDTLFSRSNEFLP-NIKYYNVHDGLP    79
                            1.......10........20........30........40........50........60........70........80

                            :*** . *  .  * ** ::  *.::: :  ** *:*: ::*:::***:**..::* *: . *:*:**:.. ** .***
Petunia_2                   EGYVVGKGGIEALIGLFFKSAKENIQNAMAAAVEESGKKITCVMADAFMWFSGEIAEELSVGWIPLWTSAAGSLSVHVYT    147
2C1Z_A|PDBID|CHAIN|SEQUENCE EGYVFA-GRPQEDIELFTRAAPESFRQGMVMAVAETGRPVSCLVADAFIWFAADMAAEMGVAWLPFWTAGPNSLSTHVYI    153
3HBF_A|PDBID|CHAIN|SEQUENCE KGYVSS-GNPREPIFLFIKAMQENFKHVIDEAVAETGKNITCLVTDAFFWFGADLAEEMHAKWVPLWTAGPHSLLTHVYT    158
                            ........90.......100.......110.......120.......130.......140.......150.......160

                            * ***:   . : . :.  : .:**:.:::  .* .*::  :::  *: ***:**  : :*.*: :***  :.  : ::*
Petunia_2                   DLIRENVEAQGIAGREDEILTFIPGFAELRLGSLPSGVVSGDLESPFSVMLHKMGKTIGKATALPVNSFEELDPPIVEDL    227
2C1Z_A|PDBID|CHAIN|SEQUENCE DEIREKIGVSGIQGREDELLNFIPGMSKVRFRDLQEGIVFGNLNSLFSRMLHRMGQVLPKATAVFINSFEELDDSLTNDL    233
3HBF_A|PDBID|CHAIN|SEQUENCE DLIREKTGSKEVHDVKS--IDVLPGFPELKASDLPEGVIK-DIDVPFATMLHKMGLELPRANAVAINSFATIHPLIENEL    235
                            .......170.......180.......190.......200.......210.......220.......230.......240

                            :**::  **:***** **     :.:  **: **.:::  **.**.**:*.**** *: *::*:** .  **:**::.
Petunia_2                   KSKFNNFLNVGPFNLTTPPPSANITDEYGCIAWLDKQEPGSVAYIGFGTVATPPPNELKAMAEALEESKTPFLWSLKDLF    307
2C1Z_A|PDBID|CHAIN|SEQUENCE KSKLKTYLNIGPFNLITPPP--VVPNTTGCLQWLKERKPTSVVYISFGTVTTPPPAEVVALSEALEASRVPFIWSLRDKA    311
3HBF_A|PDBID|CHAIN|SEQUENCE NSKFKLLLNVGPFNLTTPQR--KVSDEHGCLEWLDQHENSSVVYISFGSVVTPPPHELTALAESLEECGFPFIWSFRGDP    313
                            .......250.......260.......270.......280.......290.......300.......310.......320

                            :  :*:****:*   * :*.****.::* * :**.*:.*.****: *.:..***:*.******: **  :.*.* :***
Petunia_2                   KSFFPEGFLERTSEYGKIVSWAPQVQVLSHGSVGVFINHCGWNSVLESIAAGVPVICRPFFGDHQLNAWMVEKVWKIGVK    387
2C1Z_A|PDBID|CHAIN|SEQUENCE RVHLPEGFLEKTRGYGMVVPWAPQAEVLAHEAVGAFVTHCGWNSLWESVAGGVPLICRPFFGDQRLNGRMVEDVLEIGVR    391
3HBF_A|PDBID|CHAIN|SEQUENCE KEKLPKGFLERTKTKGKIVAWAPQVEILKHSSVGVFLTHSGWNSVLECIVGGVPMISRPFFGDQGLNTILTESVLEIGVG    393
                            .......330.......340.......350.......360.......370.......380.......390.......400

                            ::.**:**..   .::  :*.:* .  :::::   :* * .**  .*:*: :* .*:::::.
Petunia_2                   IEGGVFTKDGTMLALDLVLSKDKRNTELKQQIGMYKELALNAVGPSGSSAENFKKLVDIITSCN--    451
2C1Z_A|PDBID|CHAIN|SEQUENCE IEGGVFTKSGLMSCFDQILSQEK-GKKLRENLRALRETADRAVGPKGSSTENFITLVDLVSKPKDV    456
3HBF_A|PDBID|CHAIN|SEQUENCE VDNGVLTKESIKKALELTMSSEK-GGIMRQKIVKLKESAFKAVEQNGTSAMDFTTLIQIVTS----    454
                            .......410.......420.......430.......440.......450.......460......
```

```
                                    .     ****:****.*******:::::*: :*:. ****.*  :*  ::*       : :  *** *:: *
Populus2                    -------MSD----HVAVFAFPFGTHAAPLLAIIHRLATASPNTHFSFFSTQQSNSSIFSIYKKKMNIMQPNIKAYEVWD    69
2C1Z_A|PDBID|CHAIN|SEQUENCE --MSQTTTNP----HVAVLAFPFSTHAAPLLAVVRRLAAAAPHAVFSFFSTSQSNASIF---HDSMHTMQCNIKSYDISD    71
3HBF_A|PDBID|CHAIN|SEQUENCE MSTFKNEMNGNNLLHVAVLAFPFGTHAAPLLSLVKKIATEAPKVTFSFFCTTTTNDTLFS----RSNEFLPNIKYYNVHD    76
                            1.......10........20........30........40........50........60........70........80

                            * *:*** :*.*:* * ** ::  *.::: :  **:***: ::***:***:**..::* *: . *:*:*****:** :**
Populus2                    GAPEGYVFSGNPQEHIELFMKSARESLKKAMEVAVSETGRKVSCLVSDAFFWFACEMAEEIGVGWLPFWTAGPNSLSAHV   149
2C1Z_A|PDBID|CHAIN|SEQUENCE GVPEGYVFAGRPQEDIELFTRAAPESFRQGMVMAVAETGRPVSCLVADAFIWFAADMAAEMGVAWLPFWTAGPNSLSTHV   151
3HBF_A|PDBID|CHAIN|SEQUENCE GLPKGYVSSGNPREPIFLFIKAMQENFKHVIDEAVAETGKNITCLVTDAFFWFGADLAEEMHAKWVPLWTAGPHSLLTHV   156
                            ........90.......100.......110.......120.......130.......140.......150.......160

                            * * ***. *   :   : : :..: *:.:::  ** **::  : :  *: ***:**  **:* ** ****  :.    :
Populus2                    YTDLIRETFGDGGMVGREDKTISLIQGMSKIRICDLPEGVLFGNTESFFSNMLHKMGKALPQAAAVFINSFEELDPGTIK   229
2C1Z_A|PDBID|CHAIN|SEQUENCE YIDEIREKIGVSGIQGREDELLNFIPGMSKVRFRDLQEGIVFGNLNSLFSRMLHRMGQVLPKATAVFINSFEELDDSLTN   231
3HBF_A|PDBID|CHAIN|SEQUENCE YTDLIREKTGSKEVH--DVKSIDVLPGFPELKASDLPEGVIK-DIDVPFATMLHKMGLELPRANAVAINSFATIHPLIEN   233
                            .......170.......180.......190.......200.......210.......220.......230.......240

                            :*:*::*  **:** :*   .    : :  **: ** .::: :** *:***:*.**** *:.**:*:**  .  *****::...
Populus2                    DLKSRFKKFLNIGPSHLILSPPPMEDTYGCMTWLDKQKLASVAYVSFGSVTTPPPHELVALAEALETSETPFIWSLKDNS   309
2C1Z_A|PDBID|CHAIN|SEQUENCE DLKSKLKTYLNIGPFNLITPPPVVPNTTGCLQWLKERKPTSVVYISFGTVTTPPPAEVVALSEALEASRVPFIWSLRDKA   311
3HBF_A|PDBID|CHAIN|SEQUENCE ELNSKFKLLLNVGPFNLTTPQRKVSDEHGCLEWLDQHENSSVVYISFGSVVTPPPHELTALAESLEECGFPFIWSFRGDP   313
                            .......250.......260.......270.......280.......290.......300.......310.......320

                            :  :**.***::*    *  :*.*:** *:* *  :**.*:**.****: * .:.****:*.******* **   : *.* ***:
Populus2                    KVHLPHGFLDRTTSQGLVVPWSPQLEVLAHRAVGVFVTHCGWNSLLESIAGGVPMICRPFFGDQRLNGRMIEDVWEIGLK   389
2C1Z_A|PDBID|CHAIN|SEQUENCE RVHLPEGFLEKTRGYGMVVPWAPQAEVLAHEAVGAFVTHCGWNSLWESVAGGVPLICRPFFGDQRLNGRMVEDVLEIGVR   391
3HBF_A|PDBID|CHAIN|SEQUENCE KEKLPKGFLERTKTKGKIVAWAPQVEILKHSSVGVFLTHSGWNSVLECIVGGVPMISRPFFGDQGLNTILTESVLEIGVG   393
                            .......330.......340.......350.......360.......370.......380.......390.......400

                            ::.**:**  :  ..::   :* * *   :*:::   *:: * :*:   :*:*   :* :* ::* .
Populus2                    VEDGVFTKLEVLNSLNKILSHEGGQKMRENIRALKQLAKKAIGPNGSSINNFIALSNLVFNTKI-   453
2C1Z_A|PDBID|CHAIN|SEQUENCE IEGGVFTKSGLMSCFDQILSQEKGKKLRENLRALRETADRAVGPKGSSTENFITLVDLVSKPKDV   456
3HBF_A|PDBID|CHAIN|SEQUENCE VDNGVLTKESIKKALELTMSSEKGGIMRQKIVKLKESAFKAVEQNGTSAMDFTTLIQIVTS----   454
                            .......410.......420.......430.......440.......450.......460.....
```

```
                            **            *:******..**..***::*::::: *. . ****.*. :* .:*    . :  *** *:: **:*
Populus3                    MSEAR-----NDLKHIAVLAFPVATHGPPLLSLVRRLSASASYAKFSFFSTKESNSKLFSK-EDGLE-NIKPYNVSDGLP    73
2C1Z_A|PDBID|CHAIN|SEQUENCE MSQTT------TNPHVAVLAFPFSTHAAPLLAVVRRLAAAAPHAVFSFFSTSQSNASIFHDSMHTMQCNIKSYDISDGVP    74
3HBF_A|PDBID|CHAIN|SEQUENCE MSTFKNEMNGNNLLHVAVLAFPFGTHAAPLLSLVKKIATEAPKVTFSFFCTTTTNDTLFSRSNEFLP-NIKYYNVHDGLP    79
                            1.......10........20........30........40........50........60........70........80

                            :.*  :*.  * :  * :*   .*:: :  ** *.*: .:*:::***:**.**:* *: . *:*:**:...**   :  *
Populus3                    ENYNFAGNLDEVMNYFFKATPGNFKQAMEVAVKEVGKDFTCIMSDAFLWFAADFAQELHVPWVPLWTSSSRSLLLVLETD   153
2C1Z_A|PDBID|CHAIN|SEQUENCE EGYVFAGRPQEDIELFTRAAPESFRQGMVMAVAETGRPVSCLVADAFIWFAADMAAEMGVAWLPFWTAGPNSLSTHVYID   154
3HBF_A|PDBID|CHAIN|SEQUENCE KGYVSSGNPREPIFLFIKAMQENFKHVIDEAVAETGKNITCLVTDAFFWFGADLAEEMHAKWVPLWTAGPHSLLTHVYTD   159
                            ........90.......100.......110.......120.......130.......140.......150.......160

                             :::*   *     : . ::.:**:.:::  *: : :. .   . *: ** ::*  **:* .*  ***  :.      ::
Populus3                    LVHQKMR-SIINEPEDRTIDILPGFSELRGSDIPKELFHDVKESQFAAMLCKIGLALPQAAVVASNSFEELDPDAVILFK   232
2C1Z_A|PDBID|CHAIN|SEQUENCE EIREKIGVSGIQGREDELLNFIPGMSKVRFRDLQEGIVFGNLNSLFSRMLHRMGQVLPKATAVFINSFEELDDSLTNDLK   234
3HBF_A|PDBID|CHAIN|SEQUENCE LIREKTG-SKE-VHDVKSIDVLPGFPELKASDLPEGVIKDIDVP-FATMLHKMGLELPRANAVAINSFATIHPLIENELN   236
                            .......170.......180.......190.......200.......210.......220.......230.......240

                            *::   **:*** * :*   :.:  ***:**.:::  ********:* * ** *:. * *:*: . .**:**:*...: .
Populus3                    SRLPKFLNIGPFVLTSPDPFMSDPHGCLEWLDKQKQESVVYISFGSVITLPPQELAELVEALKECKLPFLWSFRGNPKEE   312
2C1Z_A|PDBID|CHAIN|SEQUENCE SKLKTYLNIGPFNLITPPPVVPNTTGCLQWLKERKPTSVVYISFGTVTTPPPAEVVALSEALEASRVPFIWSLRDKARVH   314
3HBF_A|PDBID|CHAIN|SEQUENCE SKFKLLLNVGPFNLTTPQRKVSDEHGCLEWLDQHENSSVVYISFGSVVTPPPHELTALAESLEECGFPFIWSFRGDPKEK   316
                            .......250.......260.......270.......280.......290.......300.......310.......320

                            **: ***:*:  * :*.*:** ::* *.::*.*:**.****: :.:.* **:*.******* :*    * *   *: ::.
Populus3                    LPEEFLERTKEKGKVVSWTPQLKVLRHKAIGVFVTHSGWNSVLDSIAGCVPMICRPFFGDQTVNTRTIEAVWGTGLEIEG   392
2C1Z_A|PDBID|CHAIN|SEQUENCE LPEGFLEKTRGYGMVVPWAPQAEVLAHEAVGAFVTHCGWNSLWESVAGGVPLICRPFFGDQRLNGRMVEDVLEIGVRIEG   394
3HBF_A|PDBID|CHAIN|SEQUENCE LPKGFLERTKTKGKIVAWAPQVEILKHSSVGVFLTHSGWNSVLECIVGGVPMISRPFFGDQGLNTILTESVLEIGVGVDN   396
                            .......330.......340.......350.......360.......370.......380.......390.......400

                            * :** .: ..:   :* ::*  :*:::  *:  *  **  .*:*: :* **:::*:.
Populus3                    GRITKGGLMKALRLIMSTDEGNKMRKKLQHLQGLALDAVQSSGSSTKNFETLLEVVAK----   450
2C1Z_A|PDBID|CHAIN|SEQUENCE GVFTKSGLMSCFDQILSQEKGKKLRENLRALRETADRAVGPKGSSTENFITLVDLVSKPKDV   456
3HBF_A|PDBID|CHAIN|SEQUENCE GVLTKESIKKALELTMSSEKGGIMRQKIVKLKESAFKAVEQNGTSAMDFTTLIQIVTS----   454
                            .......410.......420.......430.......440.......450.......460..
```

```
                            *.      .  .  *********.:******::::::*: :*:. ****.*  :* ::*  *      *** *:: **:*
Populus_1                   MP-----QTAAGPDHVAVLAFPFSSHAAPLLAIIHRLATSSPNTHFSFFSTQQSNNSIF--SIYKQNRNIKAYDVWDGVP    73
2C1Z_A|PDBID|CHAIN|SEQUENCE MS-----QTTTNP-HVAVLAFPFSTHAAPLLAVVRRLAAAAPHAVFSFFSTSQSNASIFHDSMHTMQCNIKSYDISDGVP    74
3HBF_A|PDBID|CHAIN|SEQUENCE MSTFKNEMNGNNLLHVAVLAFPFGTHAAPLLSLVKKIATEAPKVTFSFFCTTTTNDTLFSRSNEFLP-NIKYYNVHDGLP    79
                            1.......10........20........30........40........50........60........70........80

                            :*** :*.*:* * ** ::  :.*:: :  **:***: ::***:***:**..::* *: . *:*:***** ** :*** *
Populus_1                   EGYVFAGKPQEHIELFMKSAPNSFKKAMEVAVSETGRKVSCLVSDAFFWFAGEMAEEIGVVWLPFWTAGPTSLSAHVYTD   153
2C1Z_A|PDBID|CHAIN|SEQUENCE EGYVFAGRPQEDIELFTRAAPESFRQGMVMAVAETGRPVSCLVADAFIWFAADMAAEMGVAWLPFWTAGPNSLSTHVYID   154
3HBF_A|PDBID|CHAIN|SEQUENCE KGYVSSGNPREPIFLFIKAMQENFKHVIDEAVAETGKNITCLVTDAFFWFGADLAEEMHAKWVPLWTAGPHSLLTHVYTD   159
                            ........90.......100.......110.......120.......130.......140.......150.......160

                             **:. *   : . :.  :..:**:.:::  ** **::  :::  *. ***:**  **:* ** ****  :.  : .:*:
Populus_1                   LIRDTFGVGGVAGHEDELLSLIPGMSKIRIRDLPEGVLFGNLEAVFPNMLHKMGRALPKAAAVFINSFEELDPRITRDLK   233
2C1Z_A|PDBID|CHAIN|SEQUENCE EIREKIGVSGIQGREDELLNFIPGMSKVRFRDLQEGIVFGNLNSLFSRMLHRMGQVLPKATAVFINSFEELDDSLTNDLK   234
3HBF_A|PDBID|CHAIN|SEQUENCE LIREKTGSKEVHDVKS--IDVLPGFPELKASDLPEGVIK-DIDVPFATMLHKMGLELPRANAVAINSFATIHPLIENELN   236
                            .......170.......180.......190.......200.......210.......220.......230.......240

                            *::*  **:****: :*   ..:  **: **..:: :**.*:***::.**** *:.**:*:** . .*****::...: :
Populus_1                   SRFKEFLNIGPFNMISPAPPAADTYGCITWLDRQKLASVAYLSFGSITTPPPHELVALAEALETSGVPFIWSLKDNSKVH   313
2C1Z_A|PDBID|CHAIN|SEQUENCE SKLKTYLNIGPFNLITPPPVVPNTTGCLQWLKERKPTSVVYISFGTVTTPPPAEVVALSEALEASRVPFIWSLRDKARVH   314
3HBF_A|PDBID|CHAIN|SEQUENCE SKFKLLLNVGPFNLTTPQRKVSDEHGCLEWLDQHENSSVVYISFGSVVTPPPHELTALAESLEECGFPFIWSFRGDPKEK   316
                            .......250.......260.......270.......280.......290.......300.......310.......320

                            **:***::*   * :*.*:** *:* *.:**.*:**.****: *.:.****:*.******* **  :.*..  :**: ::.
Populus_1                   LPNGFLDRTTSQGLLVPWTPQMEVLAHKAVGVFITHCGWNSLLESIAGGVPMICRPFFGDQRLNGRMVEDAWKIGLQVED   393
2C1Z_A|PDBID|CHAIN|SEQUENCE LPEGFLEKTRGYGMVVPWAPQAEVLAHEAVGAFVTHCGWNSLWESVAGGVPLICRPFFGDQRLNGRMVEDVLEIGVRIEG   394
3HBF_A|PDBID|CHAIN|SEQUENCE LPKGFLERTKTKGKIVAWAPQVEILKHSSVGVFLTHSGWNSVLECIVGGVPMISRPFFGDQGLNTILTESVLEIGVGVDN   396
                            .......330.......340.......350.......360.......370.......380.......390.......400

                            **: * .: ..::  :*.:.*  :*:::  *:: * :*:  :*:*  :* :* ::*
Populus_1                   GVFRKHGVLNSLDKVLSQDSGEEMRENIRALQQLAKKAIGPNGSSINNFVSLSDLV------   449
2C1Z_A|PDBID|CHAIN|SEQUENCE GVFTKSGLMSCFDQILSQEKGKKLRENLRALRETADRAVGPKGSSTENFITLVDLVSKPKDV   456
3HBF_A|PDBID|CHAIN|SEQUENCE GVLTKESIKKALELTMSSEKGGIMRQKIVKLKESAFKAVEQNGTSAMDFTTLIQIVTS----   454
                            .......410.......420.......430.......440.......450.......460..
```

```
                            *:    :        ***.*****.***:* *::*:::*: :*:. ****.*: :* ::*  :   :  *** ::: **:
Prunus                      MAPQPIDDDHIVYEHHVAALAFPFSTHASPTLALVRRLAAASPNTLFSFFSTSQSNNSLFSNTITNLPRNIKVFDVADGV    80
2C1Z_A|PDBID|CHAIN|SEQUENCE MSQTTTN-------PHVAVLAFPFSTHAAPLLAVVRRLAAAAPHAVFSFFSTSQSNASIFHDSMHTMQCNIKSYDISDGV    73
3HBF_A|PDBID|CHAIN|SEQUENCE MSTFKNEMNG-NNLLHVAVLAFPFGTHAAPLLSLVKKIATEAPKVTFSFFCTTTTNDTLFSRSNEFLP-NIKYYNVHDGL    78
                            1.......10........20........30........40........50........60........70........80

                            *:*** :*.*:* * ** :*  ..*   :  .**.:*: ::**::***:**.*::* :: . *:*:* :* :**  **:
Prunus                      PEGYVFAGKPQEDIELFMKAAPHNFTTSLDACVAHAGKRLTCLITDAFLWFGANLAHDLGVPWLPLWLSGLNSLSLHVHT   160
2C1Z_A|PDBID|CHAIN|SEQUENCE PEGYVFAGRPQEDIELFTRAAPESFRQGMVMAVAETGRPVSCLVADAFIWFAADMAAEMGVAWLPFWTAGPNSLSTHVYI   153
3HBF_A|PDBID|CHAIN|SEQUENCE PKGYVSSGNPREPIFLFIKAMQENFKHVIDEAVAETGKNITCLVTDAFFWFGADLAEEMHAKWVPLWTAGPHSLLTHVYT   158
                            ........90.......100.......110.......120.......130.......140.......150.......160

                            * :*.. * . : . :.  :     :**:.:::  ** **::  :::  *: ***:**  **:*.** :***  :.  :
Prunus                      DLLRHTIGTQSIAGRENELITKNVNIPGMSKVRIKDLPEGVIFGNLDSVFSRMLHQMGQLLPRANAVLVNSFEELDIAVT   240
2C1Z_A|PDBID|CHAIN|SEQUENCE DEIREKIGVSGIQGREDELLN---FIPGMSKVRFRDLQEGIVFGNLNSLFSRMLHRMGQVLPKATAVFINSFEELDDSLT   230
3HBF_A|PDBID|CHAIN|SEQUENCE DLIREKTGSKEVHDVKS--ID---VLPGFPELKASDLPEGVIK-DIDVPFATMLHKMGLELPRANAVAINSFATIHPLIE   232
                            .......170.......180.......190.......200.......210.......220.......230.......240

                            *:*:**::  **:***** :.           . :  ***.**.::: :* ***:***:*. **  *: *::::** . .
Prunus                      NDLKSKFNKLLNVGPFNLAAAASPPLPEAPTAADDVTGCLSWLDKQKAASSVVYVSFGSVARPPEKELMAMAQALEASGV   320
2C1Z_A|PDBID|CHAIN|SEQUENCE NDLKSKLKTYLNIGPFNLITP--------PPVVPNTTGCLQWLKERKPTS-VVYISFGTVTTPPPAEVVALSEALEASRV   301
3HBF_A|PDBID|CHAIN|SEQUENCE NELNSKFKLLLNVGPFNLTTP--------QRKVSDEHGCLEWLDQHENSS-VVYISFGSVVTPPPHELTALAESLEECGF   303
                            .......250.......260.......270.......280.......290.......300.......310.......320

                            **:*.::.. :  * : :* ::   * :*.**** .:* * :**.*:**.**.*: * :.****:*.******* :*  :
Prunus                      PFLWPLKDSFKTPLLNELLIKAT-NGMVVPWAPQPRVLAHASVGAFVTHCGWSSLLETIAGGVPMICRPFFGDQRVNARL   399
2C1Z_A|PDBID|CHAIN|SEQUENCE PFIWSLRDKARVHLPEGFLEKTRGYGMVVPWAPQAEVLAHEAVGAFVTHCGWNSLWESVAGGVPLICRPFFGDQRLNGRM   381
3HBF_A|PDBID|CHAIN|SEQUENCE PFIWSFRGDPKEKLPKGFLERTKTKGKIVAWAPQVEILKHSSVGVFLTHSGWNSVLECIVGGVPMISRPFFGDQGLNTIL   383
                            .......330.......340.......350.......360.......370.......380.......390.......400

                            .*.*****. ::.**:** .: . ::  :*.::*  :*:::  ::  * ::*  :* *: :*  *:::::
Prunus                      VEDVLEIGATVEDGVFTKHGMIKYFDQVLSQQRGKKLRENINTVKLLARQSVEPKGGSAQNFKLLLDVISRSTKV   474
2C1Z_A|PDBID|CHAIN|SEQUENCE VEDVLEIGVRIEGGVFTKSGLMSCFDQILSQEKGKKLRENLRALRETADRAVGPKGSSTENFITLVDLVSKPKDV   456
3HBF_A|PDBID|CHAIN|SEQUENCE TESVLEIGVGVDNGVLTKESIKKALELTMSSEKGGIMRQKIVKLKESAFKAVEQNGTSAMDFTTLIQIVTS----   454
                            .......410.......420.......430.......440.......450.......460.......470.....
```

```
                            *:  ..:       *********.******* :* ::*: **:. **** *. :* :::      :  *  ::  :: **
Rosa_1                      MAPASNQVGG----HVAVLAFPFSTHAAPLLNIVCRLAAAAPNTLFSFFNTKQSNSSILASNTSSILRNSNVRVCEVADG    76
2C1Z_A|PDBID|CHAIN|SEQUENCE MSQTTTNP------HVAVLAFPFSTHAAPLLAVVRRLAAAAPHAVFSFFSTSQSNASIFHDSMHTMQCN--IKSYDISDG    72
3HBF_A|PDBID|CHAIN|SEQUENCE MSTFKNEMNGNNLLHVAVLAFPFGTHAAPLLSLVKKIATEAPKVTFSFFCTTTTNDTLFSR-SNEFLPN--IKYYNVHDG    77
                            1.......10........20........30........40........50........60........70........80

                            :*:***  *.*:* * ** :*  :.*:: :  :*****: ::***:***:**.*.:* ::    *:*:***** ** :**
Rosa_1                      VPEGYVFVGKPQEDIELFMKAAPDNFRRCLEASVAETGREVSCLVTDAFFWFGAHMADDLGGLPWVPFWTAGPASLSAHV   156
2C1Z_A|PDBID|CHAIN|SEQUENCE VPEGYVFAGRPQEDIELFTRAAPESFRQGMVMAVAETGRPVSCLVADAFIWFAADMAAEMG-VAWLPFWTAGPNSLSTHV   151
3HBF_A|PDBID|CHAIN|SEQUENCE LPKGYVSSGNPREPIFLFIKAMQENFKHVIDEAVAETGKNITCLVTDAFFWFGADLAEEMH-AKWVPLWTAGPHSLLTHV   156
                            ........90.......100.......110.......120.......130.......140.......150.......160

                            : * **:. .    : ::     . .*:.:::  ** **::  .::  *: ***:**  ** *.** ****  :.  : *
Rosa_1                      HTDLIRNTTSMGGHDGKE-TITAVTAGMSKVRPQDLPEGIIFGKLDSLFSRMLHQMGLMLPLATAVFINSFEELDPVITN   235
2C1Z_A|PDBID|CHAIN|SEQUENCE YIDEIREKIGVSGIQGREDELLNFIPGMSKVRFRDLQEGIVFGNLNSLFSRMLHRMGQVLPKATAVFINSFEELDDSLTN   231
3HBF_A|PDBID|CHAIN|SEQUENCE YTDLIREKTGSK--EVHDVKSIDVLPGFPELKASDLPEGVIK-DIDVPFATMLHKMGLELPRANAVAINSFATIHPLIEN   233
                            .......170.......180.......190.......200.......210.......220.......230.......240

                            :*:**:*  **:***:*  .   ..             ***.**.::: :****:***:*. *.* *: **:*:** . .
Rosa_1                      DLKSKFKRYLNVGPFDLLESPAPAATTTLQTGDVVVGDGCLSWLDKQKAASVVYVSFGSVTRPSPEELMALAEALEASRV   315
2C1Z_A|PDBID|CHAIN|SEQUENCE DLKSKLKTYLNIGPFNLITPPPVVPNTT----------GCLQWLKERKPTSVVYISFGTVTTPPPAEVVALSEALEASRV   301
3HBF_A|PDBID|CHAIN|SEQUENCE ELNSKFKLLLNVGPFNLTTPQRKVSDEH----------GCLEWLDQHENSSVVYISFGSVVTPPPHELTALAESLEECGF   303
                            .......250.......260.......270.......280.......290.......300.......310.......320

                            **:**:*..      . *:.::.  * :*.*.** ::* * :**.*:**.****: *.:.****:*.******* **  :
Rosa_1                      PFLWSLRNNLMTPKLDEFISKAELNGMVVPWVPQPQVLAHGSVGAFVTHCGWNSVLESLAGGVPMICRPFFGDQKLNARM   395
2C1Z_A|PDBID|CHAIN|SEQUENCE PFIWSLRDKARVHLPEGFLEKTRGYGMVVPWAPQAEVLAHEAVGAFVTHCGWNSLWESVAGGVPLICRPFFGDQRLNGRM   381
3HBF_A|PDBID|CHAIN|SEQUENCE PFIWSFRGDPKEKLPKGFLERTKTKGKIVAWAPQVEILKHSSVGVFLTHSGWNSVLECIVGGVPMISRPFFGDQGLNTIL   383
                            .......330.......340.......350.......360.......370.......380.......390.......400

                            .*.  :**: ::.**:**..: ..::  :*.:**  :*:.:  ::: * :**  :*:*: :* :*:::::.
Rosa_1                      VEDEWKIGLKLEGGVFTKNGMLKSLDILLSQKKGNIMRDTINTFKQLAQQAVEPKGSSTRNFESLLEVISTAN--   468
2C1Z_A|PDBID|CHAIN|SEQUENCE VEDVLEIGVRIEGGVFTKSGLMSCFDQILSQEKGKKLRENLRALRETADRAVGPKGSSTENFITLVDLVSKPKDV   456
3HBF_A|PDBID|CHAIN|SEQUENCE TESVLEIGVGVDNGVLTKESIKKALELTMSSEKGGIMRQKIVKLKESAFKAVEQNGTSAMDFTTLIQIVTS----   454
                            .......410.......420.......430.......440.......450.......460.......470.....
```

```
                               .   .:       ***.*****.***.***::* ::*: **.. ***: *: :* ::*         :  *:* *:: *
Rosa_2                      MAGQGPKQPSTQYHHHVAALAFPFGTHAGPLLSLVLRLAAAAPDTIFSFITTSKANSSLFSPTSKASAFL-NVKTYDVPD    79
2C1Z_A|PDBID|CHAIN|SEQUENCE -MSQTTTNP------HVAVLAFPFSTHAAPLLAVVRRLAAAAPHAVFSFFSTSQSNASIFHD--SMHTMQCNIKSYDISD    71
3HBF_A|PDBID|CHAIN|SEQUENCE -MSTFKNEMNGNNLLHVAVLAFPFGTHAAPLLSLVKKIATEAPKVTFSFFCTTTTNDTLFS---RSNEFLPNIKYYNVHD    76
                            1.......10........20........30........40........50........60........70........80

                            *:*:* * :*.*.: * :* :*    .*:  :  * *::*  : ***:***:**..*:* **   *:*:*****.** .*.
Rosa_2                      GLPEGLVPSGHPEQAIGFFLKAAPANFRSAMKEAEADSGLKIGCLVTDAFFWFAGDIAEEMKLPWVPLWTAGPRSLLVHA   159
2C1Z_A|PDBID|CHAIN|SEQUENCE GVPEGYVFAGRPQEDIELFTRAAPESFRQGMVMAVAETGRPVSCLVADAFIWFAADMAAEMGVAWLPFWTAGPNSLSTHV   151
3HBF_A|PDBID|CHAIN|SEQUENCE GLPKGYVSSGNPREPIFLFIKAMQENFKHVIDEAVAETGKNITCLVTDAFFWFGADLAEEMHAKWVPLWTAGPHSLLTHV   156
                            ........90.......100.......110.......120.......130.......140.......150.......160

                              * **::         : : :::.:**:.::.  ** **::  :::  :: :**:**  **:* ** ***:    .  : *
Rosa_2                      ATDLIRQRVLG-----TDEKTLEFLPGFSKLEVADLPEGVVSGNLESPISSLLHKMGQQLPKAAAVAINSLEEAEPDVVN   234
2C1Z_A|PDBID|CHAIN|SEQUENCE YIDEIREKIGVSGIQGREDELLNFIPGMSKVRFRDLQEGIVFGNLNSLFSRMLHRMGQVLPKATAVFINSFEELDDSLTN   231
3HBF_A|PDBID|CHAIN|SEQUENCE YTDLIREKTGSK--EVHDVKSIDVLPGFPELKASDLPEGVIK-DIDVPFATMLHKMGLELPRANAVAINSFATIHPLIEN   233
                            .......170.......180.......190.......200.......210.......220.......230.......240

                            :*:*:::  **:***** :*     : :  ***:**.::: :********:*. *** *:.*::*:*   . .*****:*..
Rosa_2                      ELKSRFRKFLNVGPFNLTSPSPPLLKDDSGCLEWLDKHKPASVVYISFGSVVRPPPHELAAFAEALIESAFPFIWSFRGN   314
2C1Z_A|PDBID|CHAIN|SEQUENCE DLKSKLKTYLNIGPFNLITPPP-VVPNTTGCLQWLKERKPTSVVYISFGTVTTPPPAEVVALSEALEASRVPFIWSLRDK   310
3HBF_A|PDBID|CHAIN|SEQUENCE ELNSKFKLLLNVGPFNLTTPQR-KVSDEHGCLEWLDQHENSSVVYISFGSVVTPPPHELTALAESLEECGFPFIWSFRGD   312
                            .......250.......260.......270.......280.......290.......300.......310.......320

                            .    **:*  :::   * :*.****.::* * :**.*:**.****  *.:.****:* ******* **    .* *  ***
Rosa_2                      PDDILPKG-CDKSSLNGKLVSWAPQVQILEHAAVGVFVTHCGWNSTLESIVGGVPMIGRPFFGDQILNMRTVETVWGIGV   393
2C1Z_A|PDBID|CHAIN|SEQUENCE ARVHLPEGFLEKTRGYGMVVPWAPQAEVLAHEAVGAFVTHCGWNSLWESVAGGVPLICRPFFGDQRLNGRMVEDVLEIGV   390
3HBF_A|PDBID|CHAIN|SEQUENCE PKEKLPKGFLERTKTKGKIVAWAPQVEILKHSSVGVFLTHSGWNSVLECIVGGVPMISRPFFGDQGLNTILTESVLEIGV   392
                            .......330.......340.......350.......360.......370.......380.......390.......400

                              ::.**:** .   ..::  :   : *  :*:::  *:: *  **  .*:*:  *  *:::.:.
Rosa_2                      GIEGGVLTKHGAIKALELILRGKAGNEMREKIKVLKNLAQSAVEGDGSSSKAFNCLVDIATNHEEV   459
2C1Z_A|PDBID|CHAIN|SEQUENCE RIEGGVFTKSGLMSCFDQILSQEKGKKLRENLRALRETADRAVGPKGSSTENFITLVDLVSKPKDV   456
3HBF_A|PDBID|CHAIN|SEQUENCE GVDNGVLTKESIKKALELTMSSEKGGIMRQKIVKLKESAFKAVEQNGTSAMDFTTLIQIVTS----   454
                            .......410.......420.......430.......440.......450.......460......
```

```
                            **       . *    ***: ***.:*.:.** .:: ::: **.   *:**.*: :* ::*   *       *:* *:: **:*
Rosa_3                      MS--HKLASSNPQAPVAVFNFPFASHPSSLLRFIRTISAVAPDIKFTFFSTSRTNSSLFSGSNNKGLDNVKPYNVWDGLP    78
2C1Z_A|PDBID|CHAIN|SEQUENCE MS----QTTTNP--HVAVLAFPFSTHAAPLLAVVRRLAAAAPHAVFSFFSTSQSNASIFHDSMHTMQCNIKSYDISDGVP    74
3HBF_A|PDBID|CHAIN|SEQUENCE MSTFKNEMNGNNLLHVAVLAFPFGTHAAPLLSLVKKIATEAPKVTFSFFCTTTTNDTLFSRSN-EFLPNIKYYNVHDGLP    79
                            1.......10........20........30........40........50........60........70........80

                            :***  :* * * * **      :.*.: :  . ****: . **::***:**..*:* :* . *:. *: **..* .*:
Rosa_3                      EGYVPPSGPPLEQIGLFLDQAPQSFERALKEVEAETGHKFGCLISDAFLWFSGDIAQKMNVPWVTTWS-GPRPLLVHLET   157
2C1Z_A|PDBID|CHAIN|SEQUENCE EGYVF-AGRPQEDIELFTRAAPESFRQGMVMAVAETGRPVSCLVADAFIWFAADMAAEMGVAWLPFWTAGPNSLSTHVYI   153
3HBF_A|PDBID|CHAIN|SEQUENCE KGYVS-SGNPREPIFLFIKAMQENFKHVIDEAVAETGKNITCLVTDAFFWFGADLAEEMHAKWVPLWTAGPHSLLTHVYT   158
                            ........90.......100.......110.......120.......130.......140.......150.......160

                            * **** *     ..    : :::.:**:. :.    **     *: :::   :: **::**  **:*.** :***   :.   : ::
Rosa_3                      DMIREKVGAPGQED---KTLDFLPGFSNEFLASDLPKEVVFGNIESPLANMLYKMGQKLPQATAVAVNSFETMDLKVSEE   234
2C1Z_A|PDBID|CHAIN|SEQUENCE DEIREKIGVSGIQGREDELLNFIPGMS-KVRFRDLQEGIVFGNLNSLFSRMLHRMGQVLPKATAVFINSFEELDDSLTND   232
3HBF_A|PDBID|CHAIN|SEQUENCE DLIREKTGSKEVHDV--KSIDVLPGFP-ELKASDLP-EGVIKDIDVPFATMLHKMGLELPRANAVAINSFATIHPLIENE   234
                            .......170.......180.......190.......200.......210.......220.......230.......240

                            *:.::*  * :**::*  *     .         :  .** **.::: :**.*****:* : ** *:.**:*:**    .**:**
Rosa_3                      LKKRLKKLLLVGPLHLVRPVPSAQSDDDEEEKDACLPWLDNHKPASVAYISFGSVGALPPMEVAALAEALEEGGFPFLWS   314
2C1Z_A|PDBID|CHAIN|SEQUENCE LKSKLKTYLNIGPFNLITPPPVVP------NTTGCLQWLKERKPTSVVYISFGTVTTPPPAEVVALSEALEASRVPFIWS   306
3HBF_A|PDBID|CHAIN|SEQUENCE LNSKFKLLLNVGPFNLTTPQRKVS------DEHGCLEWLDQHENSSVVYISFGSVVTPPPHELTALAESLEECGFPFIWS   308
                            .......250.......260.......270.......280.......290.......300.......310.......320

                            :*..    .:*:**:*:*   * :*.*. *.::* * ::*.*:** **:*: *.:.  ***:* **.*.** **    .* *
Rosa_3                      FRGN-LEDFPKGFIERTST-GRVVPWVNQVQILNHPSIGVFVTHGGWKSVLESVTCGVPMIGRPHFADQTLNMRSVEVVW   392
2C1Z_A|PDBID|CHAIN|SEQUENCE LRDKARVHLPEGFLEKTRGYGMVVPWAPQAEVLAHEAVGAFVTHCGWNSLWESVAGGVPLICRPFFGDQRLNGRMVEDVL   386
3HBF_A|PDBID|CHAIN|SEQUENCE FRGDPKEKLPKGFLERTKTKGKIVAWAPQVEILKHSSVGVFLTHSGWNSVLECIVGGVPMISRPFFGDQGLNTILTESVL   388
                            .......330.......340.......350.......360.......370.......380.......390.......400

                            :**: ::.**:**..  . ::  :* *:*  :*:.:  *:: *  .:*  :*:*: :: .
Rosa_3                      KIGMRIEGGVFTKSGAIKVLEQALSLEQGKEMRQRVGVLKQLAQETVGPNGSSAQDLKVW----------   452
2C1Z_A|PDBID|CHAIN|SEQUENCE EIGVRIEGGVFTKSGLMSCFDQILSQEKGKKLRENLRALRETADRAVGPKGSSTENFITLVDLVSKPKDV   456
3HBF_A|PDBID|CHAIN|SEQUENCE EIGVGVDNGVLTKESIKKALELTMSSEKGGIMRQKIVKLKESAFKAVEQNGTSAMDFTTLIQIVTS----   454
                            .......410.......420.......430.......440.......450.......460.......470
```

```
                                          ..   *:*:****** .:******::*:.::.  * :.  **** *: :* ::*  : .    *** *:: **:
Solanum_tuberosum            --------MTTSQLHIALLAFPFGSHAAPLLTLVQEITPFLPSNTIFSFFNTSNSNTSIFSKTPN--QENIKIYNIWDGV    70
2C1Z_A|PDBID|CHAIN|SEQUENCE  ------MSQTTTNPHVAVLAFPFSTHAAPLLAVVRRLAAAAP-HAVFSFFSTSQSNASIFHDSMHTMQCNIKSYDISDGV    73
3HBF_A|PDBID|CHAIN|SEQUENCE  MSTFKNEMNGNNLLHVAVLAFPFGTHAAPLLSLVKKIATEAP-KVTFSFFCTTTTNDTLFSRSNE-FLPNIKYYNVHDGL    78
                             1.......10........20........30........40........50........60........70........80

                              :*   .   *.   * * ** ::    .*.: :   *   ***   .:*:::***:**.:::* :*    *:.:****. **   *:*
Solanum_tuberosum            KQGNDTPIGR--EAIELFIQSTPTNFEKSMKEAEEETGVKFSCIISDAFLWFSSEFANKMNIPWIAFWTAGSCSLSIHLY    148
2C1Z_A|PDBID|CHAIN|SEQUENCE  PEG-YVFAGRPQEDIELFTRAAPESFRQGMVMAVAETGRPVSCLVADAFIWFAADMAAEMGVAWLPFWTAGPNSLSTHVY    152
3HBF_A|PDBID|CHAIN|SEQUENCE  PKG-YVSSGNPREPIFLFIKAMQENFKHVIDEAVAETGKNITCLVTDAFFWFGADLAEEMHAKWVPLWTAGPHSLLTHVY    157
                             ........90.......100.......110.......120.......130.......140.......150.......160

                              * **.           : : :  :**:.   ::   *:    ::    ::   :.  **:.*.   * :*.** :***   :.   : :
Solanum_tuberosum            TDLIRS----------NDETLLKIPGFSSTLKMSDMPPEVIAESLNGPMPSMLYNMALNLHKANAVVLNSFEELDPIINK    218
2C1Z_A|PDBID|CHAIN|SEQUENCE  IDEIREKIGVSGIQGREDELLNFIPGMS-KVRFRDLQEGIVFGNLNSLFSRMLHRMGQVLPKATAVFINSFEELDDSLTN    231
3HBF_A|PDBID|CHAIN|SEQUENCE  TDLIREKTGSK--EVHDVKSIDVLPGFP-ELKASDLPEGVIKD-IDVPFATMLHKMGLELPRANAVAINSFATIHPLIEN    233
                             .......170.......180.......190.......200.......210.......220.......230.......240

                             :*:**::   **:**: : :.    . :      **:.**.:::  ****:***:*.* ** *: *::*:**    ..****:
Solanum_tuberosum            DLKSKLQKVLNIGPLVILSSNNVFLDANSDESGCIHWLDNQKERSVVYLSFGTVTTLPPNEIIAIAEALEDKKMTFIWSL    298
2C1Z_A|PDBID|CHAIN|SEQUENCE  DLKSKLKTYLNIGPFNLITPPPVVPNT----TGCLQWLKERKPTSVVYISFGTVTTPPPAEVVALSEALEASRVPFIWSL    307
3HBF_A|PDBID|CHAIN|SEQUENCE  ELNSKFKLLLNVGPFNLTTPQRKVSDE----HGCLEWLDQHENSSVVYISFGSVVTPPPHELTALAESLEECGFPFIWSF    309
                             .......250.......260.......270.......280.......290.......300.......310.......320

                             *..     **:****:*:   *  ::.**** *:* * :**.*:**.****: *  :   ***:*.******* **    :.*.* *
Solanum_tuberosum            RDNGVKILPKGFLERTKEYGKIISWAPQLEILAHRSVGVFVTHCGWNSILEGISYGVPMICRPFFGDQKLNSRMVESVWE    378
2C1Z_A|PDBID|CHAIN|SEQUENCE  RDKARVHLPEGFLEKTRGYGMVVPWAPQAEVLAHEAVGAFVTHCGWNSLWESVAGGVPLICRPFFGDQRLNGRMVEDVLE    387
3HBF_A|PDBID|CHAIN|SEQUENCE  RGDPKEKLPKGFLERTKTKGKIVAWAPQVEILKHSSVGVFLTHSGWNSVLECIVGGVPMISRPFFGDQGLNTILTESVLE    389
                             .......330.......340.......350.......360.......370.......380.......390.......400

                             **: ::.* :**..  ..:.   :..***   :*:::   *:*.* .**    :*:*   :* .*:::*.
Solanum_tuberosum            IGLQIEGGNFTKSGTISALSTFFNEEKGKVLRKNVEGLKEKALEAVKLDNGSSIENFKVLVELVKCHKLT    448
2C1Z_A|PDBID|CHAIN|SEQUENCE  IGVRIEGGVFTKSGLMSCFDQILSQEKGKKLRENLRALRETADRAVGP-KGSSTENFITLVDLVSKPKDV    456
3HBF_A|PDBID|CHAIN|SEQUENCE  IGVGVDNGVLTKESIKKALELTMSSEKGGIMRQKIVKLKESAFKAVEQ-NGTSAMDFTTLIQIVTS----    454
                             .......410.......420.......430.......440.......450.......460.......470
```

```
                            *  :    : ***::.***.:*  .*** :* :*:  **:  ***:.*  ** :::   . * :   .*: : ****:***:* .
Vigna_mungo                 MGNSEEKKHVAVFSFPFGSHPTPLLNLVLKLTNAAPNLQFSFIGTEHSNKSLLISKPH-IPDTIKFYSISDGVPEGHVPG    79
3WC4_A|PDBID|CHAIN|SEQUENCE MKN---KQHVAIFPFPFGSHLPPLLNLVLKLAHIAPNTSFSFIGTHSSN-AFLFTKRH-IPNNIRVFTISDGIPEGHVPA    75
2C1Z_A|PDBID|CHAIN|SEQUENCE MSQTTTNPHVAVLAFPFSTHAAPLLAVVRRLAAAAPHAVFSFFSTSQSNASIFHDSMHTMQCNIKSYDISDGVPEGYVFA    80
                            1.......10........20........30........40........50........60........70........80

                            ..* * ::**  :.*:.:::*: :***.* . *:*:::***:  :  :*  :.*.*:..*.  . *** :.  : **:.
Vigna_mungo                 GHPVERVNLFLQASPQNLQKGIDMAVAHTKERVTCVISDAFVAPSLTVAQRLNVPWVPVWPPLSCSLSAHFYTELIRQTC   159
3WC4_A|PDBID|CHAIN|SEQUENCE NNPIEKLDLFLSTGPDNLRKGIELAVAETKQSVTCIIADAFVTSSLLVAQTLNVPWIAFWPNVSCSLSLYFNIDLIRDKC   155
2C1Z_A|PDBID|CHAIN|SEQUENCE GRPQEDIELFTRAAPESFRQGMVMAVAETGRPVSCLVADAFIWFAADMAAEMGVAWLPFWTAGPNSLSTHVYIDEIREKI   160
                            ........90.......100.......110.......120.......130.......140.......150.......160

                            .      :  *:*:**:**:*..*: :.::   *: ::***: *  :* ***:* **.:* * *** . :.: ::**:
Vigna_mungo                 NSA----AGDTPLDFVPGLSKMRVEDLPEDVIQGAGEEETLFSKTLASLGSVLPQAEAVVVNFFEELDPPLLVNDMKSKF   235
3WC4_A|PDBID|CHAIN|SEQUENCE SKD----AKNATLDFLPGLSKLRVEDVPQDMLD-VGEKETLFSRTLNSLGVVLPQAKAVVVNFFAELDPPLFVKYMRSKL   230
2C1Z_A|PDBID|CHAIN|SEQUENCE GVSGIQGREDELLNFIPGMSKVRFRDLQEGIVF--GNLNSLFSRMLHRMGQVLPKATAVFINSFEELDDS-LTNDLKSKL   237
                            .......170.......180.......190.......200.......210.......220.......230.......240

                            :  * :    . *  *     .  :  .***.**..:.   **.*:.****.:*** *:**::**** *  .**:*:*::
Vigna_mungo                 KYYLYVGFLTLSLPLPPLPPSDTDETGCLSWLDKQKGGSVVYVSFGTVVTPPPHEIVAVAEALEASGFPFLWSLKEHLKG   315
3WC4_A|PDBID|CHAIN|SEQUENCE QSLLYV----VPLPCPQLLLPEIDSNGCLSWLDSKSSRSVAYVCFGTVVSPPPQEVVAVAEALEESGFPFVWALKESLLS   306
2C1Z_A|PDBID|CHAIN|SEQUENCE KTYLNIG--PFNLITPPPVVP--NTTGCLQWLKERKPTSVVYISFGTVTTPPPAEVVALSEALEASRVPFIWSLRDKARV   313
                            .......250.......260.......270.......280.......290.......300.......310.......320

                             **:**:*:*   * ** *.**:.**.* :**.****** **: **::.***:********: : .*:::*: *:** :*
Vigna_mungo                 VLPNGFLERTSERGKVVGWAPQTQVLGHGSVGVFVTHCGCNSVFESMSNGVPMICRPFFGDHGLTGRMVEDVWEIGVRVE   395
3WC4_A|PDBID|CHAIN|SEQUENCE ILPKGFVERTSTRGKVVSWVPQSHVLSHGSVGVFVTHCGANSVMESVSNGVPMICRPFFGDQGIAARVIQDIWEVGVIVE   386
2C1Z_A|PDBID|CHAIN|SEQUENCE HLPEGFLEKTRGYGMVVPWAPQAEVLAHEAVGAFVTHCGWNSLWESVAGGVPLICRPFFGDQRLNGRMVEDVLEIGVRIE   393
                            .......330.......340.......350.......360.......370.......380.......390.......400

                            * ****.*::..:  ** :*.*: ::.*   ::: .  *.*.:*.:::* ***:::* .
Vigna_mungo                 GGVFTKDGLLKSLRLILVEEEGNLMKKNAVKVKKTVLDAAGAQGKAAQDFNTLVELVSRS---   455
3WC4_A|PDBID|CHAIN|SEQUENCE GKVFTKNGFVKSLNLILVQEDGKKIRDNALKVKQIVQDAVGPHGQAAEDFNTLVEVISSS---   446
2C1Z_A|PDBID|CHAIN|SEQUENCE GGVFTKSGLMSCFDQILSQEKGKKLRENLRALRETADRAVGPKGSSTENFITLVDLVSKPKDV   456
                            .......410.......420.......430.......440.......450.......460...
```
