# Supplementary figures and images for: In-Silico Analysis of Binding Site Features and Substrate Selectivity in Plant Flavonoid-3-O Glycosyltransferases (F3GT) through Molecular Modeling, Docking and Dynamics Simulation Studies
Source: PLoS One. 2014 Mar 25;9(3):e92636. doi: 10.1371/journal.pone.0092636 (PMC3965439; doi:10.1371/journal.pone.0092636)

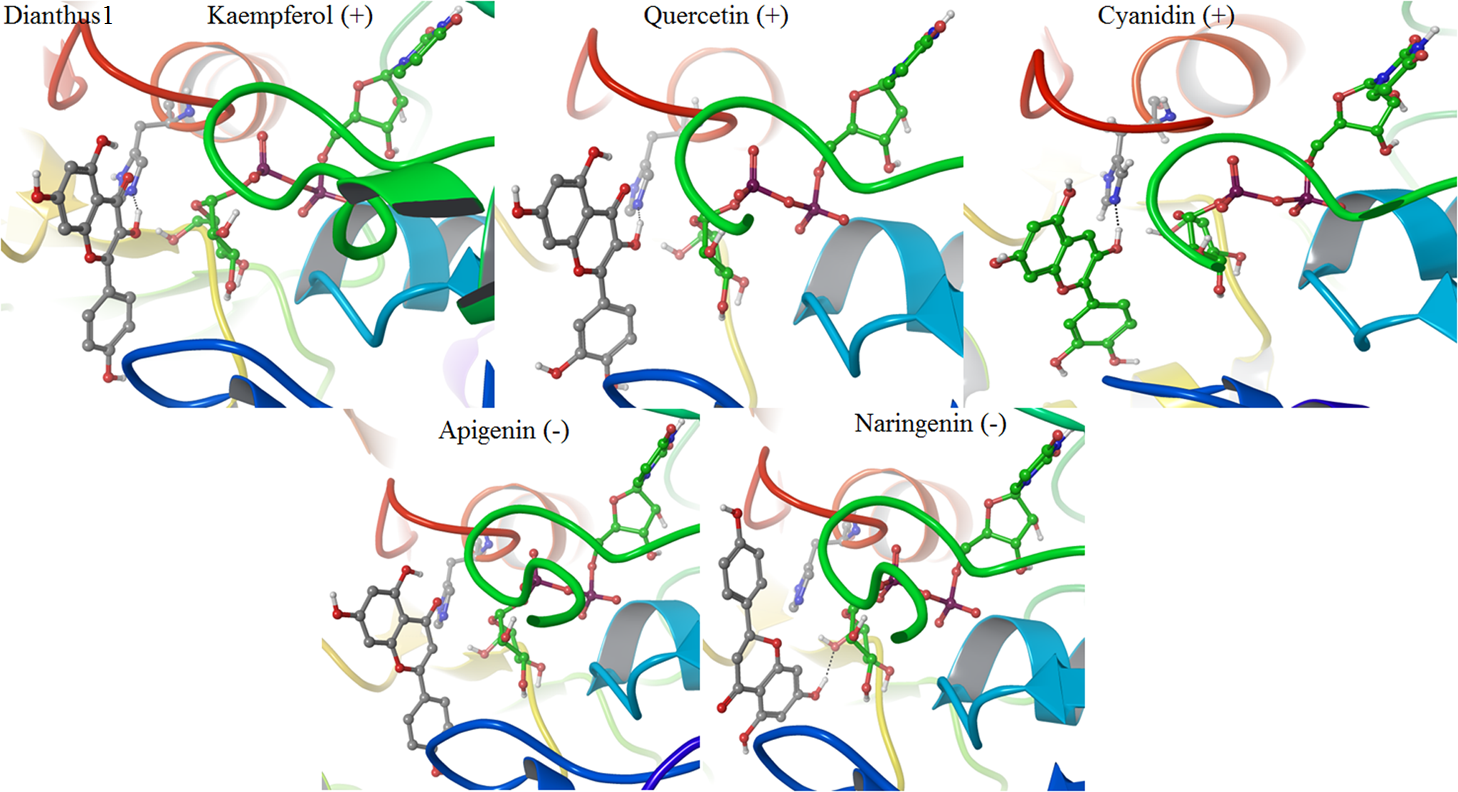

Supplement: Figure S3 — Image showing docked complexes of three positive and two negative control ligands in the acceptor binding pocket of Dianthus caryophyllus ( Dianthus_1 ). (TIF) [file pone.0092636.s003.tif]

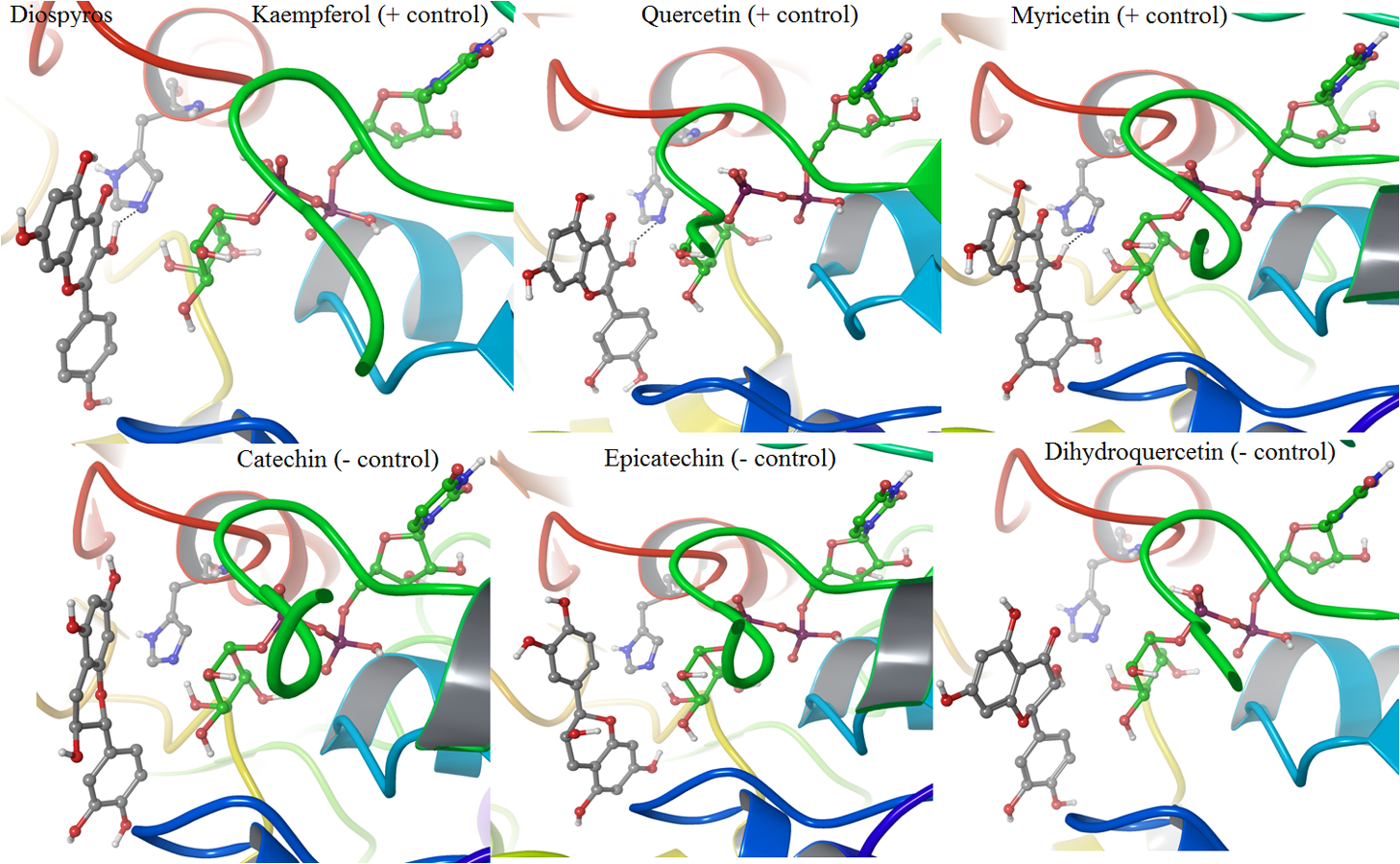

Supplement: Figure S4 — Image showing docked complexes of three positive and three negative control ligands in the acceptor binding pocket of Diospyros kaki . (TIF) [file pone.0092636.s004.tif]

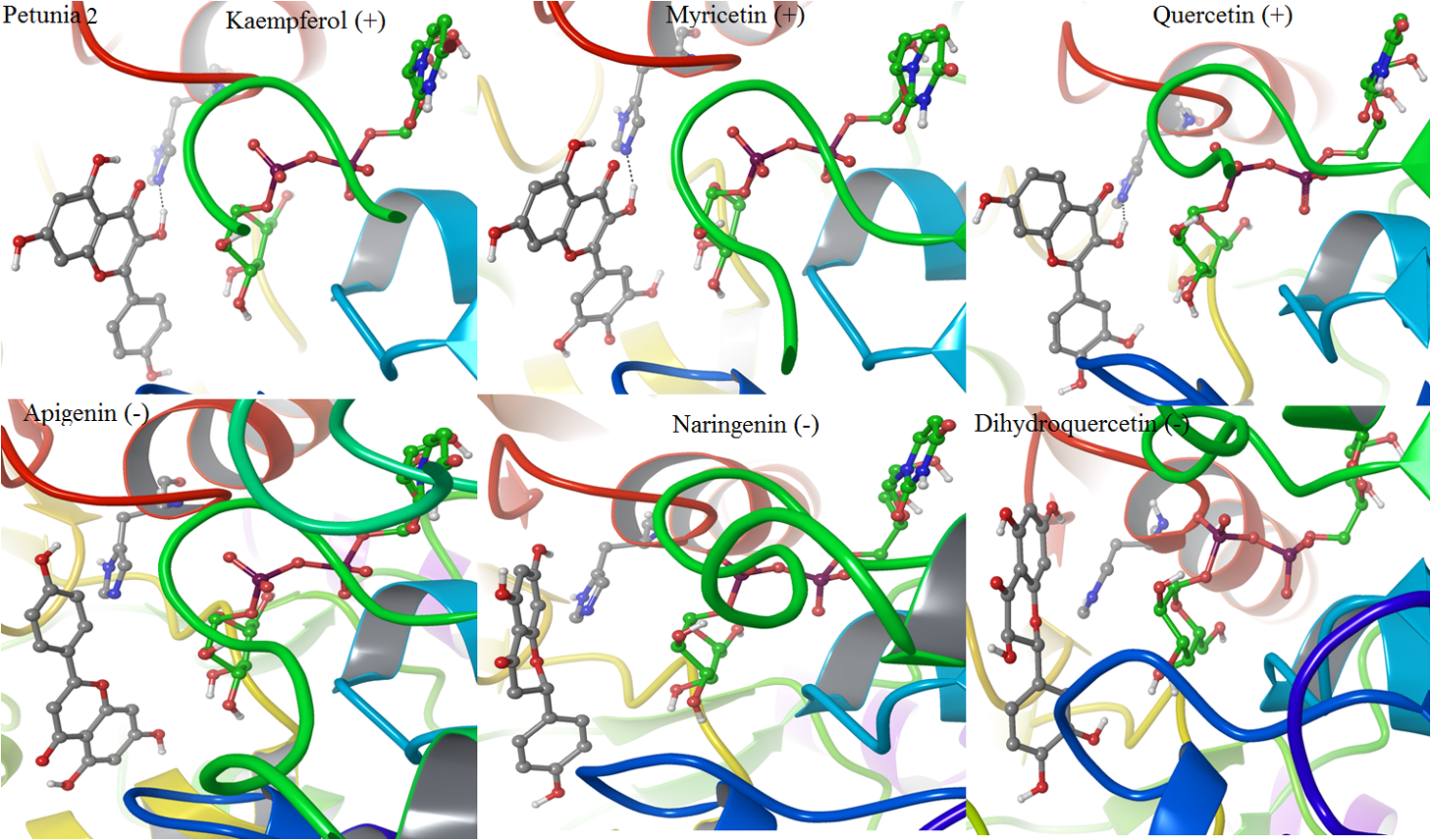

Supplement: Figure S5 — Image showing docked complexes of three positive and three negative control ligands in the acceptor binding pocket of Petunia hybrida ( Petunia_2 ). (TIF) [file pone.0092636.s005.tif]

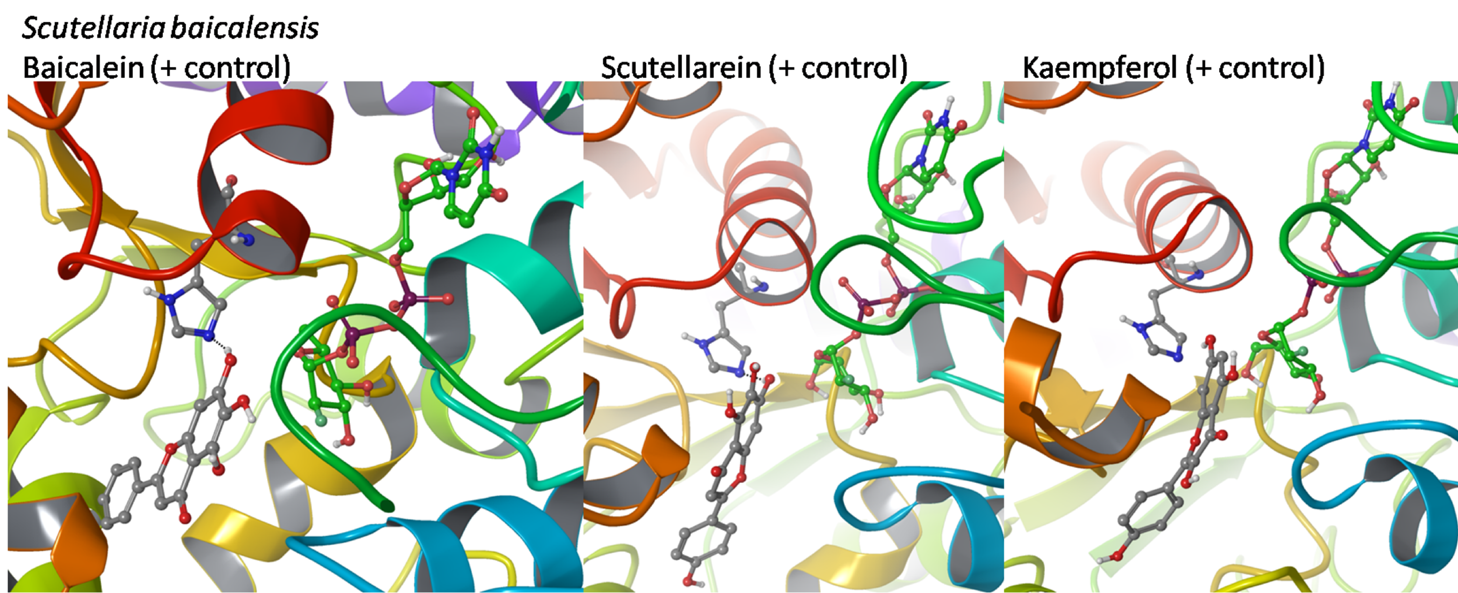

Supplement: Figure S6 — Image showing docked complexes of three positive control ligands in the acceptor binding pocket of Scutellaria baicalensis . (TIF) [file pone.0092636.s006.tif]
